# Effects of SlowMo, a Blended Digital Therapy Targeting Reasoning, on Paranoia Among People With Psychosis: A Randomized Clinical Trial

**DOI:** 10.1001/jamapsychiatry.2021.0326

**Published:** 2021-04-07

**Authors:** Philippa Garety, Thomas Ward, Richard Emsley, Kathryn Greenwood, Daniel Freeman, David Fowler, Elizabeth Kuipers, Paul Bebbington, Mar Rus-Calafell, Alison McGourty, Catarina Sacadura, Nicola Collett, Kirsty James, Amy Hardy

**Affiliations:** 1 Department of Psychology, Institute of Psychiatry, Psychology and Neuroscience, King’s College London, London, United Kingdom; 2 South London and Maudsley NHS (National Health Service) Foundation Trust, London, United Kingdom; 3 Department of Biostatistics and Health Informatics, Institute of Psychiatry, Psychology and Neuroscience, King’s College London, London, United Kingdom; 4 University of Sussex School of Psychology, Brighton, United Kingdom; 5 Sussex Partnership NHS Foundation Trust, Worthing, United Kingdom; 6 Department of Psychiatry, Oxford University, Oxford, United Kingdom; 7 Oxford Health NHS Foundation Trust, Oxford, United Kingdom; 8 Division of Psychiatry, University College London, London, United Kingdom; 9 Mental Health Research and Treatment Center, Faculty of Psychology, Ruhr-Universität Bochum, Bochum, Germany; 10 Oxford Institute of Clinical Psychology Training and Research, Oxford University, Oxford, United Kingdom

## Abstract

**Importance:**

Persistent paranoia is common among patients with psychosis. Cognitive-behavioral therapy for psychosis can be effective. However, challenges in engagement and effectiveness remain.

**Objective:**

To investigate the effects on paranoia and mechanisms of action of SlowMo, a digitally supported reasoning intervention, plus usual care compared with usual care only.

**Design, Setting, and Participants:**

This parallel-arm, assessor-blinded, randomized clinical trial recruited participants at UK community health services from May 1, 2017, to May 14, 2019. Eligible participants consisted of a referral sample with schizophrenia-spectrum psychosis and distressing, persistent (≥3 months) paranoia.

**Interventions:**

Individuals were randomized 1:1 to SlowMo, consisting of 8 digitally supported face-to-face sessions and a mobile app, plus usual care (n = 181) and usual care only (n = 181).

**Main Outcomes and Measures:**

The primary outcome was paranoia, measured by the Green et al Paranoid Thoughts Scale (GPTS) total score at 24 weeks. Secondary outcomes included GPTS total score at 12 weeks and GPTS Part A (reference) and Part B (persecutory) scores, the Psychotic Symptom Rating Scales (PSYRATS Delusion subscale), reasoning (belief flexibility, possibility of being mistaken [Maudsley Assessment of Delusions, rated 0%-100%]), and jumping to conclusions (Beads Task).

**Results:**

A total of 361 participants were included in intention-to-treat analysis, of whom 252 (69.8%) were male and 249 (69.0%) were White; the mean (SD) age was 42.6 (11.6) years. At 24 weeks, 332 participants (92.0%) provided primary outcome data. Of 181 participants in the SlowMo group, 145 (80.1%) completed therapy. SlowMo plus usual care was not associated with greater reductions than usual care in GPTS total score at 24 weeks (Cohen *d*, 0.20; 95% CI, −0.02 to 0.40; *P* = .06). There were significant effects on secondary paranoia outcomes at 12 weeks, including GPTS total score (Cohen *d*, 0.30; 95% CI, 0.09-0.51; *P* = .005), Part A score (Cohen *d*, 0.22; 95% CI, 0.06-0.39; *P* = .009), and Part B score (Cohen *d*, 0.32; 95% CI, 0.08-0.56; *P* = .009), and at 24 weeks, including Part B score (Cohen *d*, 0.25; 95% CI, 0.01-0.49; *P* = .04) but not Part A score (Cohen *d*, 0.12; 95% CI, −0.05 to 0.28; *P* = .18). Improvements were observed in an observer-rated measure of persecutory delusions (PSYRATS delusion) at 12 weeks (Cohen *d*, 0.47; 95% CI, 0.17-0.78; *P* = .002) and 24 weeks (Cohen *d*, 0.50; 95% CI, 0.20-0.80; *P* = .001) and belief flexibility at 12 weeks (Cohen *d*, 0.29; 95% CI, 0.09-0.49; *P* = .004) and 24 weeks (Cohen *d*, 0.28; 95% CI, 0.08-0.49; *P* = .005). There were no significant effects on jumping to conclusions. Improved belief flexibility and worry mediated paranoia change (range mediated, 36%-56%).

**Conclusions and Relevance:**

SlowMo did not demonstrate significant improvements in the primary measure of paranoia at 24 weeks; however, a beneficial effect of SlowMo on paranoia was indicated by the results on the primary measure at an earlier point and on observer-rated paranoia and self-reported persecution at 12 and 24 weeks. Further work to optimize SlowMo’s effects is warranted.

**Trial Registration:**

isrctn.org Identifier: ISRCTN 32448671

## Key Points

**Question:**

Can a brief blended digital therapy targeting reasoning (SlowMo) improve paranoia for adults with psychosis when added to usual care?

**Findings:**

This randomized clinical trial of 361 individuals with clinical paranoia did not demonstrate that SlowMo therapy reduced the primary outcome of self-reported paranoia at 24 weeks compared with usual care only, although secondary beneficial effects were found on this measure at 12 weeks. Self-reported persecution and observer-rated paranoia were improved at both points.

**Meaning:**

SlowMo, a digitally supported reasoning intervention, indicated a beneficial effect on paranoia; further work to optimize the effects of SlowMo is warranted.

## Teaser

This randomized clinical trial investigates the effects and mechanisms of action of SlowMo, a digitally supported reasoning intervention, plus usual care compared with usual care only among individuals with clinical paranoia.

## Introduction

Paranoia, or fear of deliberate harm from others, is among the most common symptoms of schizophrenia spectrum disorders and is associated with substantial distress and disruption.^1^ Developing effective interventions for paranoia is a clinical priority. Meta-analyses of first-generation cognitive-behavioral therapy for psychosis (CBTp) have indicated associations with delusions^2^ and broader positive symptoms.^3^ However, marked challenges to treatment engagement, adherence, and effectiveness remain.^1,4^

SlowMo therapy adopts an interventionist-causal approach^5^ to increasing CBTp effectiveness by targeting reasoning processes considered causal in paranoia.^6^ These biased processes include jumping to conclusions (JTC) (ie, forming rapid judgments using limited information) and belief inflexibility (reduced metacognitive capacity for reflecting on and reviewing one’s beliefs and considering alternatives).^6,7,8^ SlowMo aims to build awareness of a tendency to JTC and develop increased belief flexibility. SlowMo is the end point of a decade of development, during which preliminary evidence that the intervention reduced paranoia severity, mediated by increased belief flexibility, was found.^9,10,11,12^ Over time, the intervention has focused increasingly on belief flexibility, adopting the terms *fast and slow thinking* to communicate reasoning concepts.^8,13,14^ SlowMo also uses digital technology and inclusive, human-centered design to improve the user experience with the aim of enhancing engagement and adherence for the widest possible range of people.^14,15,16^ SlowMo builds on the encouraging findings for stand-alone and blended mobile phone apps for psychosis^17,18,19,20^ and, to our knowledge, is the first blended digital psychological intervention for paranoia (using digitally supported face-to-face therapy and a mobile app).

This randomized clinical trial aimed to test the efficacy of SlowMo in reducing paranoia and improving reasoning. We hypothesized that SlowMo would improve paranoia and reasoning together with outcomes prioritized by the trial’s service-user consultants: self-concept, quality of life, and well-being. We also hypothesized that the treatment effects on paranoia would be mediated through reasoning, specifically belief flexibility and JTC. We also examined worry as an outcome and mediator because worry mediates change in paranoia.^21^ However, because worry was not directly targeted by the treatment, we hypothesized that worry would not mediate the treatment effects of SlowMo on paranoia.

## Methods

### Research Design

This parallel-group randomized clinical trial (ISRCTN32448671) used 1:1 allocation and blinded assessors to test the efficacy of adding SlowMo therapy to treatment as usual (TAU) to reduce paranoia severity compared with TAU alone (trial protocol given in Supplement 1). The trial was performed from May 1, 2017, to October 31, 2019. Recruitment was from UK community mental health services across 3 main sites. The trial received ethical approval from the Camberwell St Giles research ethics committee, and all participants gave written informed consent. This study followed the Consolidated Standards of Reporting Trials (CONSORT) reporting guideline.^22^

### Participants

Eligible participants met the following criteria: 18 years or older; persistent (≥3 months) distressing paranoia (assessed using the Schedules for Clinical Assessment in Neuropsychiatry^23^); score of greater than 29 on the Green et al Paranoid Thoughts Scale (GPTS) Part B, the Persecutory subscale^24^; a diagnosis of schizophrenia spectrum psychosis (codes F20-29 from the *International Statistical Classification of Diseases and Related Health Problems, Tenth Revision*)^25^; capacity to provide informed consent; and sufficient English to participate in trial processes. Participants were excluded if they had profound visual or hearing impairment, were unable to engage in assessments, were currently receiving psychological therapy for paranoia, and had a primary diagnosis of substance use disorder, personality disorder, organic syndrome, or learning disability.

### Randomization and Masking

After baseline assessment, we randomly assigned (1:1) eligible patients using a secure, independent, web-based service hosted by King’s Clinical Trials Unit. Randomly varying sized blocks were used and stratified by site and baseline paranoia (median split of ≥62 on GPTS Part B score^24^). Research assessors were masked to allocation. The site coordinators (T.W., M.R.-C., A.M., C.S., and N.C.) conducted randomization and informed participants. If unmasking occurred, reallocation to another rater occurred when operationally feasible. Breaks in masking were recorded.

### Interventions

SlowMo is a digitally supported CBTp consisting of 8 individual, face-to-face sessions (60-90 minutes) in accordance with a clinical manual that was delivered within 12 weeks. The intervention builds awareness of unhelpful fast thinking and supports individualized formulation. SlowMo then assists people with slowing down for a moment to find ways of feeling safer. Sessions are assisted by the SlowMo web app delivered using a touchscreen laptop, with interactive features including information, animated vignettes, games, and personalized thought bubbles. The web app synchronizes to a native android mobile app providing access in daily life to SlowMo strategies and individualized safer-thought bubbles.^14,26^ A device was provided to all participants. Behavioral work outside the clinic room was encouraged, with the aim of practicing strategies. Therapy was delivered in clinic settings or at home (eMethods 1 and eFigure 1 in Supplement 2 give further details on the intervention).

Therapists included 11 trained doctoral-level psychologists (M.R.-C., T.W., A.M., C.S., and N.C. and 6 others), with therapists supervised weekly using recorded sessions. Therapy uptake was assessed by number and duration of sessions attended, with fidelity to the clinical manual defined as no more than 1 web app component missed per session (mean calculated across all attended sessions). Mobile app adherence was operationalized as at least 1 home screen interaction after a minimum of 3 therapy sessions and was recorded by system analytics (eMethods 2 in Supplement 2).

Treatment as usual was delivered according to UK national and local service guidelines and typically involved antipsychotic therapy, contact with a mental health worker, and outpatient psychiatric appointments. Participation did not alter pharmacologic or psychosocial treatment decisions (recorded in both groups using the modified Client Service Receipt Inventory^27^).

### Measurements

Assessments were performed at 0 (baseline), 12 weeks (postintervention), and 24 weeks (follow-up). Blinded assessors conducted enrollment and assessments at clinics or in participants’ homes. Participants were compensated £20 (approximately US $28) at each point.

### Outcomes

The primary outcome was self-reported paranoia severity at 24 weeks, measured by the GPTS total score^24^ (range, 32-160, with higher scores indicating more severe paranoia). The GPTS consists of two 16-item subscales assessing ideas of social reference (Part A) and persecution (Part B) during the previous month, with reported scores being secondary outcomes. Detail on all measures is provided in Supplement 1 and eMethods 3 and 4 in Supplement 2. Secondary paranoia measures also consisted of 2 observer-rated scales: the Psychotic Symptom Rating Scales (PSYRATS) delusions subscale,^28^ scored as a total and as 2 factors (conviction and distress^29^), and individual persecutory delusions and ideas of reference items from the Scales for Assessment of Positive Symptoms (SAPS).^30^ We also assessed outcomes on the Revised GPTS (R-GPTS)^31^ (total and subscale scores); this revised measure was published during the trial and was added to the statistical analysis plan before statistical analysis commenced (Supplement 1). It consists of 2 scales assessing thinking relevant to paranoia based on the original items: ideas of social reference (8 items) and persecution (10 items).

Reasoning was assessed as an outcome and as a potential mediator by 2 established methods of assessing belief flexibility relating to delusions^8^: possibility of being mistaken (self-rated 0%-100% and observer-rated yes or no) from the Maudsley Assessment of Delusions Schedule^32^ and alternative explanations from the Explanations of Experiences interview,^33^ with increased flexibility being desirable; and by the JTC Beads Task,^7^ versions 85:15 and 60:40. The Fast and Slow Thinking Questionnaire^34^ (previously the TAPS^26^), consisting of 2 scales, one assessing fast (intuitive) thinking and one measuring slow (analytic) thinking, was included as a reasoning outcome but was not prespecified as a hypothesized mediator. Other secondary outcomes were well-being (the Warwick-Edinburgh Mental Well-being Scale^35^), quality of life (the Manchester Short Assessment of Quality of Life^36^), self and other schemas (the Brief Core Schema Scales^37^), and worry (the Penn State Worry Questionnaire^38^). Adverse events were actively monitored throughout the study until the 24-week follow-up.

### Statistical Analysis

We powered the study to detect a 10-point reduction in the GPTS total score (effect size, 0.40) and accounted for the partial nested design owing to therapist clustering in the SlowMo arm.^39^ With 1:1 allocation and a statistical significance level of 2-tailed *P* < .05, a simple 2-tailed *t* test with 150 people per group had 90% power to detect an effect size of 0.40 and 80% power for an effect size of 0.35. To allow for 20% attrition, we aimed to recruit 360 patients at baseline split equally across 3 sites. All analyses were performed using the intention-to-treat population. Statisticians (R.E. and K.J.) were only unblinded after database lock, and the statistical analysis was performed unblinded owing to the need to account for therapist effects in the SlowMo arm. No interim analysis was performed. All analyses were conducted in Stata, version 16.0 (StataCorp LLC).^40^

To test the primary hypothesis that the intervention would reduce paranoia severity during the 24-week study, we fitted a linear mixed model allowing for clustering by participants and therapists to the repeated measures of the GPTS with fixed effects of randomization, time, time by randomization interaction, site, paranoia severity (stratifier), and baseline GPTS. The treatment effect (adjusted between-group mean difference) was estimated from the model for each point separately. All secondary outcome measures were analyzed using linear mixed models for continuous outcomes and logistic mixed models for binary outcomes. Cohen *d* effect sizes at 12 and 24 weeks were calculated as the adjusted mean difference divided by the sample SD of the outcome at baseline and are shown in a forest plot. Mediation analysis used parametric regression models, whereas moderation analyses were conducted by adding interaction terms between randomized groups and a set of prespecified moderators; further detail of the moderation and mediation analyses and the methods and results of a compliance-adjusted analysis are provided in eMethods 5, 6, and 8 in Supplement 2.

Missing data on measures were prorated if more than 90% of items were completed; otherwise the measure was considered missing. We checked for covariates associated with missing outcomes by comparing responders with nonresponders on key baseline variables. Maximum likelihood estimation in the mixed models accounted for missing outcome data under a missing-at-random assumption, conditional on the covariates included in the model.

## Results

**Figure 1.**
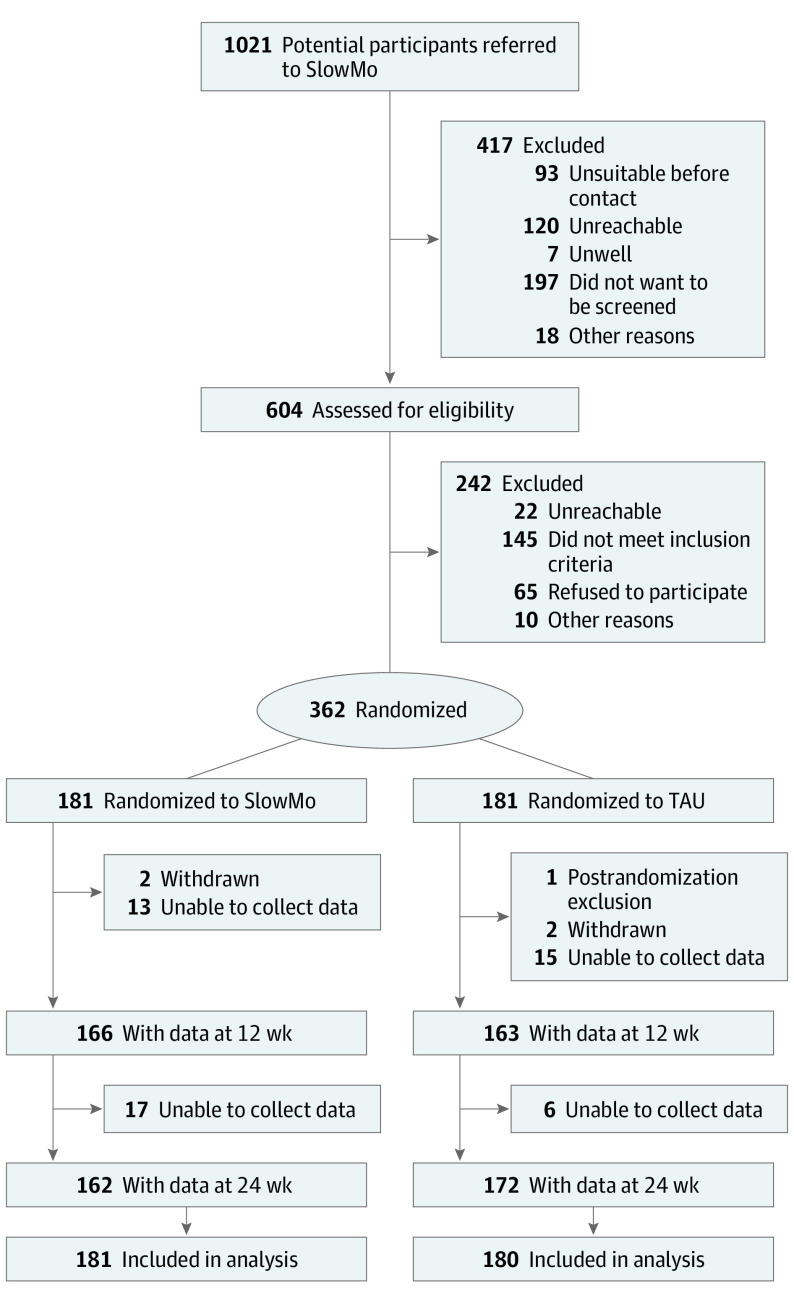
Trial Profile TAU indicates treatment as usual.

Of 604 people assessed for eligibility, 362 were recruited; 181 were randomized to the SlowMo group and 181 to the TAU group (Figure 1). One participant in the TAU group withdrew consent to use data after randomization. The final sample was therefore 361 participants.

Data on the primary outcome were available on 328 participants (90.6%) at 12 weeks and 332 (92.0%) at 24 weeks. Unmasking without replacement of an assessor occurred for 22 participants (6.7%) at 12 weeks and 19 participants (5.7%) at 24 weeks (eTable 1 in Supplement 2).

Participant baseline characteristics and stratification factors (paranoia severity and site) are shown in Table 1. Typical of samples with persisting psychosis, participants were predominantly male (252 [69.8%]) and White (249 [69.0%]), with a mean (SD) age of 42.6 (11.6) years. Other clinical characteristics (diagnosis, years in contact with services, and medication equivalent doses) were also unexceptional. There were no marked differences between the groups. Most participants had severe paranoia; 170 (94.4%) in the TAU arm and 169 (93.4%) in the SlowMo arm met criteria^31^ for likely presence of persecutory delusions on the GPTS (eTable 2 in Supplement 2). Baseline characteristics are also shown by site in eTable 3 in Supplement 2. eTable 4 in Supplement 2 shows baseline values of clinical and cognitive measures examined as putative moderators.

**Table 1.** Baseline Characteristics of the Intention-to-Treat Population

Characteristic	Study arm^a^		
	SlowMo (n = 181)	TAU (n = 180)	Overall (N = 361)
Age, mean (SD), y	43.1 (11.7)	42.2 (11.6)	42.6 (11.6)
Sex			
Male	132 (72.9)	120 (66.7)	252 (69.8)
Female	49 (27.1)	60 (33.3)	109 (30.2)
Marital status			
Single	145 (80.1)	137 (76.1)	282 (78.1)
Cohabiting	6 (3.3)	6 (3.3)	12 (3.3)
Married or civil partnership	22 (12.2)	24 (13.3)	46 (12.7)
Divorced	7 (3.9)	10 (5.6)	17 (4.7)
Widowed	1 (0.6)	3 (1.7)	4 (1.1)
Self-defined race/ethnicity			
White	120 (66.3)	129 (71.7)	249 (69.0)
Black Caribbean	9 (5.0)	9 (5.0)	18 (5.0)
Black African	12 (6.6)	10 (5.6)	22 (6.1)
Black other	16 (8.8)	12 (6.7)	28 (7.8)
Indian	0	3 (1.7)	3 (0.8)
Pakistani	4 (2.2)	4 (2.2)	8 (2.2)
Chinese	1 (0.6)	0	1 (0.3)
Other	19 (10.5)	12 (6.7)	31 (8.6)
Missing	0	1 (0.6)	1 (0.3)
Highest level of schooling			
Primary school	4 (2.2)	3 (1.7)	7 (1.9)
Secondary, no examinations or qualifications	30 (16.6)	34 (18.9)	64 (17.7)
Secondary O/CSE equivalent	50 (27.6)	51 (28.3)	101 (28.0)
Secondary A-level equivalent	23 (12.7)	16 (8.9)	39 (10.8)
Vocational education or college	43 (23.8)	44 (24.4)	87 (24.1)
University degree or professional qualification	31 (17.1)	30 (16.7)	61 (16.9)
Missing	0	2 (1.1)	2 (0.6)
Current working status			
Unemployed	141 (77.9)	150 (83.3)	291 (80.6)
Employed			
Full-time	8 (4.4)	8 (4.4)	16 (4.4)
Part-time	15 (8.3)	14 (7.8)	29 (8.0)
Self-employed	4 (2.2)	2 (1.1)	6 (1.7)
Retired	10 (5.5)	2 (1.1)	12 (3.3)
Student	1 (0.6)	3 (1.7)	4 (1.1)
Housewife or househusband	2 (1.1)	1 (0.6)	3 (0.8)
Normal living situation			
Alone	108 (59.7)	103 (57.2)	211 (58.4)
With partner	19 (10.5)	28 (15.6)	47 (13.0)
With parents	25 (13.8)	30 (16.7)	55 (15.2)
With other relatives	4 (2.2)	4 (2.2)	8 (2.2)
With others	25 (13.8)	15 (8.3)	40 (11.1)
Site			
London	66 (36.5)	64 (35.6)	130 (36.0)
Oxford	49 (27.1)	50 (27.8)	99 (27.4)
Sussex	66 (36.5)	66 (36.7)	132 (36.6)
GPTS Part B score (stratification factor)			
<62	110 (60.8)	109 (60.6)	219 (60.7)
≥62	71 (39.2)	71 (39.4)	142 (39.3)
Diagnosis			
Schizophrenia	116 (64.1)	109 (60.6)	225 (62.3)
Schizoaffective disorder	30 (16.6)	34 (18.9)	64 (17.7)
Delusional disorder	3 (1.7)	3 (1.7)	6 (1.7)
Psychosis (other)	32 (17.7)	34 (18.9)	66 (18.3)
Time in contact with services, y			
<1	7 (3.9)	6 (3.3)	13 (3.6)
1-5	22 (12.2)	33 (18.3)	55 (15.2)
6-10	40 (22.1)	44 (24.4)	84 (23.3)
11-20	70 (38.7)	70 (38.9)	140 (38.8)
>20	42 (23.2)	27 (15.0)	69 (19.1)
Chlorpromazine-equivalent dose of antipsychotic drug, mean (SD), mg/d	452.96 (399.45)	519.97 (419.80)	486.37 (410.53)

Abbreviations: GPTS, Green et al Paranoid Thoughts Scale; O/CSE, O-level/Certificate of Secondary Education; TAU, treatment as usual.

^a^ Unless otherwise indicated, data are expressed as number (percentage) of patients.

The mean (SD) number of SlowMo sessions attended was 6.8 (2.6), increasing to 7.3 (1.9) for those attending 1 or more sessions. Among the 181 participants in the SlowMo arm, 145 (80.1%) completed all 8 therapy sessions, 13 (7.2%) attended no sessions, and 23 (12.7%) discontinued therapy between sessions 1 and 7. Mean (SD) session duration, including behavioral work, was 75 (29) minutes. Therapy fidelity was high; of the 168 individuals who attended at least 1 session, 159 (94.6%) met a priori criteria for web app delivery, and 100 of 140 (71.4%) met adherence criteria for mobile app use.

**Figure 2.**
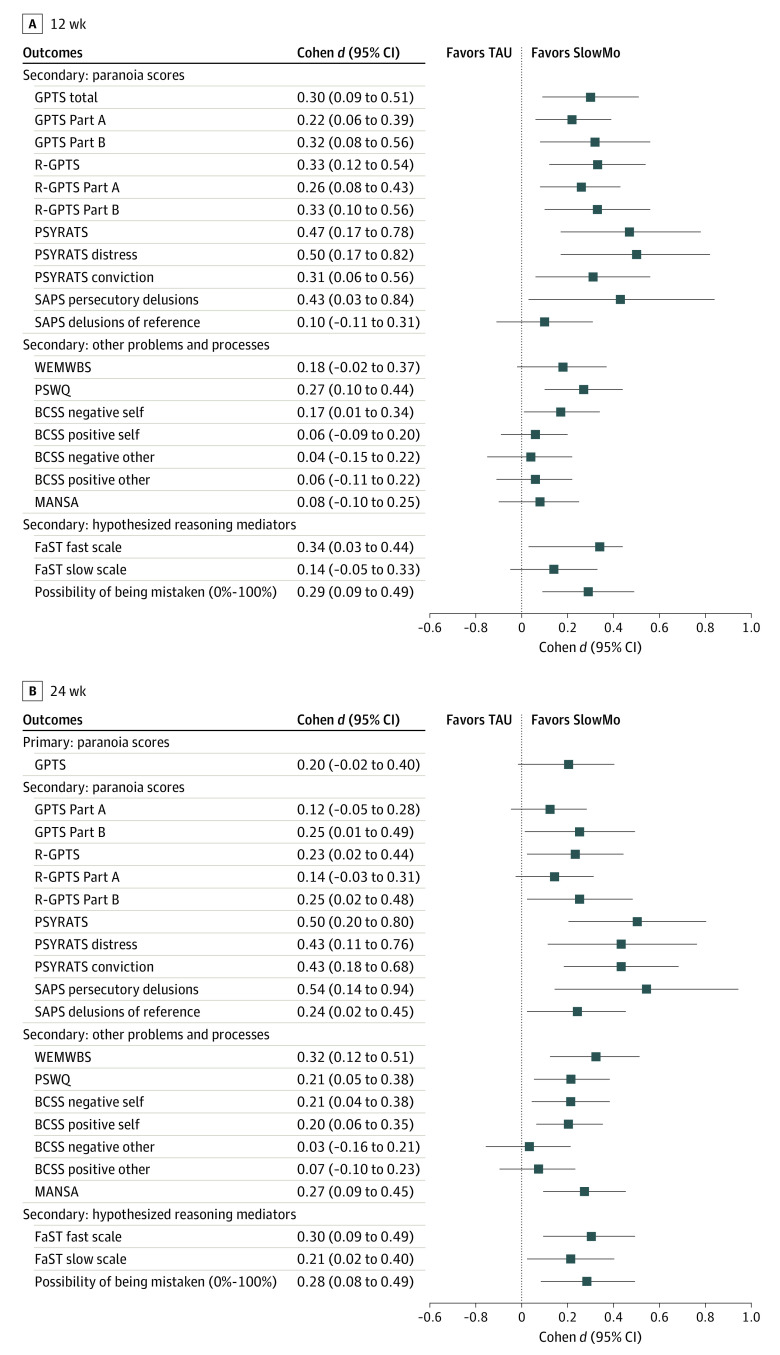
Primary and Secondary Outcomes of SlowMo Markers represent point estimates, with horizontal lines representing 95% CIs. BCSS indicates Brief Core Schema Scales; FaST, Fast and Slow Thinking Questionnaire; GPTS, Green et al Paranoid Thoughts Scales; MANSA, Manchester Short Assessment of Quality of Life; PSWQ, Penn State Worry Questionnaire; PSYRATS, Psychotic Symptom Rating Scales; R-GPTS, Revised GPTS; SAPS, Scale for the Assessment of Positive Symptoms; TAU, treatment as usual; and WEMWBS, Warwick-Edinburgh Mental Well-being Scale.

Descriptive statistics, between-group mean differences and their associated *P* values and 95% CIs, and standardized effect sizes for all primary and secondary paranoia outcomes are given in Table 2. Figure 2 shows standardized effect sizes for continuous variables as a forest plot (eFigure 2 in Supplement 2 gives a forest plot of binary secondary outcomes). SlowMo plus TAU was not associated with greater reductions than TAU alone in the primary outcome of GPTS total paranoia score at 24 weeks (Cohen *d*, 0.20; 95% CI, −0.02 to 0.40; *P* = .06). At 12 weeks, SlowMo plus TAU was associated with greater reductions than TAU alone in GPTS total score (Cohen *d*, 0.30; 95% CI, 0.09-0.51; *P* = .005), Part A score (Cohen *d*, 0.22; 95% CI, 0.06-0.39; *P* = .009), and Part B score (Cohen *d*, 0.32; 95% CI, 0.08-0.56; *P* = .009). At 24 weeks, SlowMo was significantly associated with lower Part B score (Cohen *d*, 0.25; 95% CI, 0.01-0.49; *P* = .04) but not Part A score (Cohen *d*, 0.12; 95% CI, −0.05 to 0.28; *P* = .18).

**Table 2.** Primary and Secondary Paranoia Outcomes

Outcome	SlowMo group^a^		TAU group^a^		Adjusted mean difference (SE) [95% CI]	*P* value	Cohen *d* effect size (95% CI)^b^
	Mean (SD)	No. of participants	Mean (SD)	No. of participants			
**Primary**							
GPTS total score							
Baseline	104.7 (27.6)	180	105.9 (26.0)	179	NA	NA	NA
24 wk	81.7 (31.6)	161	86.3 (33.2)	171	−5.27 (2.84) [−10.83 to 0.29]	.06	0.20 (−0.02 to 0.40)
**Secondary**							
GPTS total score							
Baseline	104.7 (27.6)	180	105.9 (26.0)	179	NA	NA	NA
12 wk	84.8 (30.8)	165	92.5 (33.1)	163	−8.06 (2.85) [−13.64 to −2.48]	.005	0.30 (0.09 to 0.51)
GPTS Part A score							
Baseline	48.6 (15.9)	181	50.3 (15.1)	179	NA	NA	NA
12 wk	40.2 (14.9)	165	44.2 (15.8)	163	−3.49 (1.34) [−6.12 to −0.86]	.009	0.22 (0.06 to 0.39)
24 wk	39.2 (15.0)	161	42.0 (15.8)	172	−1.79 (1.34) [−4.41 to −0.83]	.180	0.12 (−0.05 to 0.28)
GPTS Part B score							
Baseline	56.2 (14.4)	180	55.9 (13.8)	180	NA	NA	NA
12 wk	44.6 (18.1)	166	48.2 (18.7)	163	−4.51 (1.71) [−7.87 to −1.15]	.009	0.32 (0.08 to 0.56)
24 wk	42.2 (18.2)	161	45.1 (18.9)	171	−3.53 (1.71) [−6.89 to −0.18]	.04	0.25 (0.01 to 0.49)
R-GPTS total score							
Baseline	40.7 (15.4)	179	41.5 (14.8)	180	NA	NA	NA
12 wk	29.6 (17.2)	166	34.3 (18.6)	163	−5.00 (1.61) [−8.16 to −1.86]	.002	0.33 (0.12 to 0.54)
24 wk	27.5 (17.6)	160	31.1 (18.6)	169	−3.42 (1.61) [−6.57 to −0.27]	.03	0.23 (0.02 to 0.44)
R-GPTS social reference score							
Baseline	16.0 (7.8)	180	17.1 (8.2)	180	NA	NA	NA
12 wk	11.9 (7.6)	166	14.2 (8.1)	163	−2.05 (0.70) [−3.42 to −0.68]	.003	0.26 (0.08 to 0.43)
24 wk	11.3 (7.6)	160	13.1 (8.0)	172	−1.15 (0.70) [−2.51 to 0.22]	.099	0.14 (−0.03 to 0.31)
R-GPTS persecution score							
Baseline	24.7 (9.3)	180	24.4 (8.7)	180	NA	NA	NA
12 wk	17.7 (11.1)	166	20.1 (11.7)	163	−2.97 (1.07) [−5.07 to −0.88]	.005	0.33 (0.10 to 0.56)
24 wk	16.3 (11.2)	161	18.0 (11.6)	169	−2.25 (1.07) [−4.34 to −0.16]	.04	0.25 (0.02 to 0.48)
PSYRATS total score							
Baseline	16.5 (3.3)	180	16.2 (3.1)	180	NA	NA	NA
12 wk	13.2 (4.9)	166	14.5 (5.0)	162	−1.53 (0.50) [−2.50 to −0.56]	.002	0.47 (0.17 to 0.78)
24 wk	12.5 (5.2)	161	14.0 (5.5)	171	−1.62 (0.49) [−2.59 to −0.65]	.001	0.50 (0.20 to 0.80)
PSYRATS distress score							
Baseline	8.1 (1.8)	181	7.9 (1.7)	180	NA	NA	NA
12 wk	6.3 (2.8)	166	7.0 (2.8)	162	−0.87 (0.29) [−1.44 to −0.30]	.003	0.50 (0.17 to 0.82)
24 wk	6.0 (3.0)	161	6.8 (3.0)	171	−0.76 (0.29) [−1.32 to −0.19]	.009	0.43 (0.11 to 0.76)
PSYRATS conviction score							
Baseline	8.4 (2.0)	180	8.3 (1.9)	180	NA	NA	NA
12 wk	6.9 (2.5)	166	7.4 (2.6)	163	−0.62 (0.25) [−1.11 to −0.13]	.01	0.31 (0.06 to 0.56)
24 wk	6.4 (2.5)	161	7.2 (2.8)	172	−0.84 (0.25) [−1.33 to −0.35]	.001	0.43 (0.18 to 0.68)
SAPS persecutory delusions score							
Baseline	3.5 (0.8)	181	3.4 (0.9)	180	NA	NA	NA
12 wk	2.8 (1.3)	164	3.0 (1.3)	161	−0.37 (0.18) [−0.71 to −0.03]	.04	0.43 (0.03 to 0.84)
24 wk	2.5 (1.5)	161	2.8 (1.4)	171	−0.46 (0.18) [−0.80 to −0.12]	.009	0.54 (0.14 to 0.94)
SAPS ideas and delusions of reference score							
Baseline	2.5 (1.8)	181	2.7 (1.7)	180	NA	NA	NA
12 wk	2.2 (1.9)	165	2.4 (1.8)	161	−0.18 (0.19) [−0.55 to 0.19]	.35	0.10 (−0.11 to 0.31)
24 wk	1.9 (1.9)	160	2.4 (1.9)	171	−0.41 (0.19) [−0.79 to −0.04]	.03	0.24 (0.02 to 0.45)

Abbreviations: GPTS, Green et al Paranoid Thoughts Scale; NA, not applicable; PSYRATS, Psychotic Symptom Rating Scales; R-GPTS, Revised GPTS; SAPS, Scale for the Assessment of Positive Symptoms; TAU, treatment as usual.

^a^ Low score indicates better outcomes.

^b^ Negative effects indicate benefit of SlowMo compared with TAU.

SlowMo was also associated with improvements in observer-rated measures of persecutory delusions, including PSYRATS Delusions subscale score at 12 (Cohen *d*, 0.47; 95% CI, 0.17-0.78; *P* = .002) and 24 (Cohen *d*, 0.50; 95% CI, 0.20-0.80; *P* = .001) weeks and SAPS Persecutory Delusions subscale score at 12 (Cohen *d*, 0.43; 95% CI, 0.03-0.84; *P* = .04) and 24 (Cohen *d*, 0.54; 95% CI, 0.14-0.94; *P* = .009) weeks. The reduction in paranoia on the R-GPTS total score was significant at both points (Cohen *d* at 12 weeks, 0.33 [95% CI, 0.12-0.54; *P* = .002]; Cohen *d* at 24 weeks, 0.23 [95% CI, 0.02-0.44; *P* = .03]), as were the reductions on the PSYRATS distress (Cohen *d* at 12 weeks, 0.50 [95% CI, 0.17-0.82; *P* = .003]; Cohen *d* at 24 weeks, 0.43 [95% CI, 0.11-0.76; *P* = .009]) and conviction (Cohen *d* at 12 weeks, 0.31 [95% CI, 0.06-0.56; *P* = .01]; Cohen *d* at 24 weeks, 0.43 [95% CI, 0.18-0.68; *P* = .001]) subscale scores. Referential ideas assessed using GPTS Part A, R-GPTS Reference, and SAPS Ideas of Reference subscale scores showed less consistent effects, with significant effects either at 12 weeks or 24 weeks but not both.

Treatment effects were found for some but not all reasoning measures. Belief flexibility (possibility of being mistaken) improved at 12 (Cohen *d*, 0.29; 95% CI, 0.09-0.49; *P* = .004) and 24 (Cohen *d*, 0.28; 95% CI, 0.08-0.49; *P* = .005) weeks but alternative explanations did not. The binary measure of the possibility of being mistaken was significant at 12 but not 24 weeks. Jumping to conclusions did not improve (with 1 significant finding, beads drawn at 12 weeks). The fast-thinking scale of the Fast and Slow Thinking Questionnaire showed improvements at both time points, and the slow-thinking scale showed improvement at 24 weeks. Significant improvements were found for SlowMo in well-being (Cohen *d* at 24 weeks, 0.32; 95% CI, 0.12-0.51; *P* = .001), quality of life (Cohen *d* at 24 weeks, 0.27; 95% CI, 0.09-0.45; *P* = .003), worry (Cohen *d* at 12 weeks, 0.27 [95% CI, 0.10-0.44; *P* = .002]; Cohen *d* at 24 weeks, 0.21 [95% CI, 0.05-0.38; *P* = .01]), and self-concept (Cohen *d* for negative self at 12 weeks, 0.17 [95% CI, 0.01-0.34; *P* = .04]; Cohen *d* for negative self at 24 weeks, 0.21 [95% CI, 0.04-0.38; *P* = .01]; Cohen *d* for positive self at 24 weeks, 0.20 [95% CI, 0.06-0.35; *P* = .006]) (Table 3 and Figure 2).

**Table 3.** Secondary Outcomes and Hypothesized Mediators

Outcome	SlowMo group^a^		TAU group^a^		Adjusted mean difference (SE) [95% CI]^b^	*P* value
	Mean (SD)	Participants, No,	Mean (SD)	Participants, No.		
WEMWBS score^c^						
Baseline	39.3 (9.1)	179	40.5 (8.7)	175	NA	NA
12 wk	42.2 (9.4)	164	41.6 (9.1)	157	1.56 (0.89) [−0.18 to 3.30]	.08
24 wk	43.3 (11.0)	157	41.2 (9.6)	165	2.82 (0.89) [1.08 to 4.56]	.001
PSWQ score						
Baseline	56.9 (10.8)	179	56.6 (10.1)	175	NA	NA
12 wk	53.2 (11.6)	158	55.4 (11.5)	157	−2.81 (0.90) [−4.57 to −1.04]	.002
24 wk	52.2 (11.6)	154	54.5 (11.5)	163	−2.24 (0.90) [−4.00 to −0.48]	.01
BCSS negative self score						
Baseline	9.9 (5.8)	181	10.3 (5.5)	178	NA	NA
12 wk	9.0 (6.0)	162	10.0 (6.0)	159	−0.98 (0.48) [−1.92 to −0.04]	.04
24 wk	8.4 (5.9)	160	9.7 (5.8)	167	−1.19 (0.48) [−2.12 to −0.25]	.01
BCSS positive self score^c^						
Baseline	10.7 (5.6)	181	10.8 (5.4)	178	NA	NA
12 wk	11.5 (5.6)	164	11.5 (5.6)	159	0.33 (0.41) [−0.48 to 1.13]	.43
24 wk	12.5 (5.5)	159	11.6 (5.8)	168	1.11 (0.41) [0.31 to 1.92]	.006
BCSS negative other score						
Baseline	13.3 (6.1)	181	13.3 (5.8)	178	NA	NA
12 wk	12.9 (6.1)	163	13.0 (6.0)	159	−0.21 (0.55) [−1.30 to 0.88]	.70
24 wk	12.6 (6.2)	159	12.7 (6.3)	168	−0.16 (0.55) [−1.25 to 0.92]	.77
BCSS positive other score^c^						
Baseline	11.6 (5.2)	180	11.1 (4.9)	177	NA	NA
12 wk	12.2 (5.1)	164	11.8 (4.8)	159	0.28 (0.42) [−0.55 to 1.12]	.50
24 wk	12.4 (4.8)	158	12.1 (4.8)	168	0.34 (0.42) [−0.49 to 1.17]	.42
MANSA score^c^						
Baseline	46.8 (9.9)	161	48.1 (10.2)	164	NA	NA
12 wk	48.1 (10.7)	145	48.9 (10.6)	146	0.76 (0.91) [−1.02 to 2.55]	.40
24 wk	50.5 (11.7)	135	49.1 (9.5)	148	2.75 (0.92) [0.94 to 4.55]	.003
Possibility of being mistaken (0%-100%)^c^						
Baseline	34.6 (30.9)	181	35.1 (31.0)	180	NA	NA
12 wk	48.9 (32.2)	165	39.9 (33.2)	161	9.02 (3.16) [2.83 to 15.21]	.004
24 wk	45.3 (31.8)	161	37.7 (31.1)	172	8.88 (3.16) [2.70 to 15.07]	.005
Possibility of being mistaken, No. yes/no (% yes/% no)^c^						
Baseline	105/76 (58/42)	NA	106/74 (59/41)	NA	NA	NA
12 wk	124/41 (75/25)	NA	100/61 (62/38)	NA	3.83 (1.53 to 9.59)^d^	.004
24 wk	105/56 (65/35)	NA	100/72 (58/42)	NA	2.01 (0.86 to 4.70)^d^	.11
Alternative explanations, No. yes/no (% yes/% no)^c^						
Baseline	79/102 (44/56)	NA	85/94 (48/52)	NA	NA	NA
12 wk	90/74 (55/45)	NA	78/83 (48/52)	NA	1.74 (0.90 to 3.36)^d^	.10
24 wk	87/73 (54/46)	NA	87/82 (52/48)	NA	1.33 (0.70 to 2.55)^d^	.39
JTC version 85:15, No. yes/no (% yes/% no)						
Baseline	103/77 (57/43)	NA	83/96 (46/54)	NA	NA	NA
12 wk	70/95 (42/58)	NA	68/91 (42/58)	NA	0.71 (0.31 to 1.62)^d^	.42
24 wk	55/105 (34/66)	NA	62/107 (37/63)	NA	0.58 (0.25 to 1.34)^d^	.20
JTC version 85:15 (No. of beads)						
Baseline	3.8 (4.4)	NA	3.9 (4.0)	NA	NA	NA
12 wk	4.3 (4.3)	NA	4.1 (3.9)	NA	0.39 (0.43) [−0.45 to 1.22]	.37
24 wk	5.2 (4.8)	NA	4.1 (3.3)	NA	0.99 (0.42) [0.16 to 1.83]	.02
JTC version 60:40 (yes or no)						
Baseline	72/108 (40/60)	NA	59/120 (33/67)	NA	NA	NA
12 wk	47/118 (29/71)	NA	42/117 (26/74)	NA	0.82 (0.26 to 2.51)^d^	.72
24 wk	43/117 (27/73)	NA	44/125 (26/74)	NA	0.69 (0.22 to 2.18)^d^	.53
JTC version 60:40 (No. of beads)						
Baseline	5.7 (5.4)	NA	5.7 (5.1)	NA	NA	NA
12 wk	7.0 (5.7)	NA	6.8 (5.4)	NA	0.28 (0.49) [−0.68 to 1.25]	.56
24 wk	7.0 (5.2)		6.5 (4.9)		0.49 (0.49) [−0.47 to 1.45]	.32
FaST fast scale score						
Baseline	16.9 (4.7)	174	16.7 (4.3)	169	NA	NA
12 wk	15.3 (4.9)	165	16.2 (5.0)	160	−1.07 (0.47) [−1.98 to −0.16]	.02
24 wk	15.0 (4.4)	160	16.2 (5.1)	168	−1.33 (0.46) [−2.23 to −0.42]	.004
FaST slow scale score^c^						
Baseline	19.9 (4.7)	174	19.7 (4.8)	169	NA	NA
12 wk	20.3 (4.8)	165	19.3 (4.8)	160	0.66 (0.45) [−0.22 to 1.55]	.14
24 wk	20.3 (4.4)	160	19.3 (4.8)	168	1.00 (0.45) [0.12 to 1.88]	.03

Abbreviations: BCSS, Brief Core Schema Scale; FaST, Fast and Slow Thinking Questionnaire; JTC, jumping to conclusions; MANSA, Manchester Short Assessment of Quality of Life; NA, not applicable; PSWQ, Penn State Worry Questionnaire; TAU, treatment as usual; WEMWBS, Warwick-Edinburgh Mental Well-being Scale.

^a^ Unless otherwise indicated, low score indicates better outcomes.

^b^ Unless otherwise indicated, negative effects indicate benefit of SlowMo compared with TAU.

^c^ High score indicates better outcomes; positive effects indicate benefit of SlowMo compared with TAU.

^d^ Odds ratio (95% CI).

The moderation analysis (eTable 5 in Supplement 2) revealed no differential effects on paranoia as measured by the GPTS or R-GPTS.^31^ There were 2 moderation effects (on PSYRATS), at *P* < .05. However, given the number of tests, this finding may have occurred by chance.

The mediation analysis results on the GPTS, R-GPTS, and PSYRATS at 12 and 24 weeks are shown in eTables 6 to 8 in Supplement 2. The possibility of being mistaken (0%-100%)^32^ and worry^38^ mediated the treatment effects on all paranoia outcomes at 12 and 24 weeks. Approximately 40% of the total effect was mediated through each mediator at 12 weeks and 56% at 24 weeks.

Fifty-four adverse events were reported, of which 51 were serious, occurring in 19 participants in the SlowMo group and 21 in the TAU group; no deaths were recorded (eMethods 7 and eTable 9 in Supplement 2). A compliance-adjusted analysis showed significant treatment effects of SlowMo therapy on the primary outcome compared with TAU in those adherent to treatment at all points, with treatment effects increasing as the number of sessions increased (eTable 10 in Supplement 2). Data on concomitant treatments and service use are shown in eMethods 9 and eTables 11 and 12, respectively, in Supplement 2.

## Discussion

Treatment with SlowMo, a brief blended digital therapy, did not result in significant improvements in the primary outcome of total GPTS paranoia score at 24 weeks. However, the pattern of results indicates that SlowMo had a beneficial effect on paranoia in general. Effects on total self-rated GPTS paranoia after treatment and on self-rated GPTS persecution during the 24-week study and significant sustained moderate effects on all observer-rated measures of persecutory delusions were seen. SlowMo treatment was associated with improvements in reasoning, in belief flexibility (possibility of being mistaken), and Fast and Slow Thinking Questionnaire scores. Change in both belief flexibility and worry mediated improvements in paranoia. There were effects on outcomes prioritized at the design stage by service-user consultants^41^ in well-being, self-esteem, quality of life, and worry, with the most consistent change at 24 weeks. Therapy uptake and adherence were high. Treatment effects were not moderated by clinical or demographic variables, indicating benefits regardless of cognitive capacity, symptoms, or caregiver relationships. There was no evidence of the intervention being harmful, with similar numbers of serious adverse events in both groups.

Although GPTS effects were small, most met the threshold of *P* < .05, suggesting consistent effects on secondary self-reported paranoia outcomes. Of note, the effects for the observer-rated and widely used PSYRATS total score were in the moderate range. Although adjustment of type I error in the reporting of secondary outcomes in clinical trials is not mandated,^42^ this improvement (at *P* < .001) would remain significant at 24 weeks even if a conservative adjustment for multiple testing were to be applied. In addition to reduced PSYRATS conviction scores, the clinically important target of distress also showed a sustained reduction. Taken together, the secondary paranoia outcomes indicate small to moderate effects that were equal to or greater than rates reported in meta-analyses of longer-term CBTp for delusions.^2,43^ However, given this overall pattern of results, the absence of an effect on the primary outcome of GPTS total score at 24 weeks and the failure to reach the a priori threshold for clinical importance merit further consideration. Examination of the results and the GPTS subscales constituting the total score indicates that persecutory beliefs showed stronger effects across a range of measures, whereas milder referential ideas (self or observer rated) did not show consistent improvement. One potential explanation may be that as persecutory beliefs improved, they changed into milder ideas of reference (thus shifting down the hierarchy of paranoid beliefs^44,45^), but that the therapy prevented such ideas and their experiential components from being elaborated into paranoid fears of intentional harm. Given this finding, we believe that future iterations of SlowMo therapy should enhance work on referential ideas.

High uptake, fidelity, and adherence and the absence of moderation by baseline characteristics suggest that the inclusive, human-centered design facilitated engagement across a wide range of users and settings, which is crucial to real-world implementation.^15,16^ Usability and acceptability will be the subjects of future studies. Barriers to accessing psychological therapy for paranoia are widely reported,^46^ with effective, usable brief treatments such as SlowMo offering a potential solution.

A second goal was to evaluate reasoning as a mechanism. Improvements were observed in belief flexibility. Consistent with a proof-of-concept study,^9^ the possibility of being mistaken mediated the change in paranoia, explaining 36% to 56% of the variance after the intervention and at follow-up. In contrast, JTC showed little evidence of change. This finding, together with meta-analytic results,^47^ suggests that JTC may be associated with vulnerability to persecutory beliefs but be relatively unresponsive to change over time. This evolving understanding supports foregrounding the promotion of slow thinking and greater flexibility with the aim of generating compensatory strategies for real-world fast thinking.^8^

Worry also mediated paranoia reduction, with a similar proportion of the variance explained by the mediation by belief flexibility. This was not hypothesized because worry was not explicitly targeted in SlowMo. However, given that worry causes paranoia^21^ and that SlowMo altered worry, the finding suggests that worry reduction plausibly constitutes part of the treatment route for SlowMo. Of note, SlowMo shares features with worry interventions.^21^ Both involve noticing thoughts, decentering, and refocusing attention. Furthermore, the extent to which worry and belief flexibility are independent routes to change or whether other mechanisms for treatment effects, such as the parallel improvements in self-concept and well-being, might occur could not be determined in the present study. Our original hypotheses derived from a theory of change in which the primary process underpinning SlowMo was via reasoning. However, the evidence from this study suggests the potential for other processes also to be involved in treatment effects. Our cognitive model of paranoia proposes multifactorial causality, particularly highlighting both reasoning and emotional processes.^48^ We plan additional investigations of these and other potential mechanisms to inform further causal understanding of paranoia.

### Limitations

This study has limitations. The trial design did not control for effects of time with a therapist, with TAU being selected as the comparator condition. There is a low penetration of evidence-based psychological treatment in clinical services,^49^ and thus a key efficacy question is whether SlowMo therapy confers benefits beyond those of TAU. Garety et al^9^ previously established the superiority of an earlier brief version of the intervention against an active control intervention. Examination of mechanisms of change also required a control condition as much as possible inert with respect to reasoning. A further limitation is that our primary outcome, the GPTS score, uses self-report and was revised during the trial.^31^ However, the more psychometrically robust revision^31^ yielded similar results but with slightly larger effects. In addition, the use of blinded observer-rated measures of delusions (yielding moderate effect sizes) was consistent with improvement in clinically severe paranoia. Furthermore, we did not assess functioning; however, we did measure quality of life^36^ and well-being,^35^ indicating improvements in satisfaction with a range of domains of everyday life and function.

## Conclusions

This is the first randomized clinical trial, to our knowledge, to test a blended digital therapy for paranoia in people with psychosis. Although no effect was demonstrated on the primary paranoia outcome at 24 weeks, the pattern of results on secondary outcomes indicates SlowMo had a positive effect on paranoia, mostly sustained at follow-up, that matched or exceeded effects observed for standard CBTp albeit delivered in fewer sessions.^50^ Improvements in well-being, quality of life, and self-concept also occurred. The results indicate that the treatment was effective, in part, through helping people to slow down their thinking and to worry less. Further understanding of the mechanisms of action of SlowMo is warranted. The trial results also indicate the need for future work to enhance and translate the effects of SlowMo.

## Notes

Supplement 1. Trial Protocol

Supplement 2. **eMethods 1.** SlowMo Therapy Description **eFigure 1.** SlowMo Webapp Formulation **eMethods 2.** SlowMo Mobile App Adherence Criterion **eMethods 3.** Data Quality and Interrater Reliability **eMethods 4.** Data Completeness and Timing of Assessments **eTable 1.** Unblinding by Site **eTable 2.** Number (Percentage) Above Threshold for a Potential Persecutory Delusion **eFigure 2.** Forest Plot Binary Outcomes **eTable 3.** Baseline Characteristics by Site **eMethods 5.** Moderation Analysis: Further Detail **eTable 4.** Baseline Clinical and Cognitive Characteristics as Potential Moderators of the Intention-to-Treat Population **eTable 5.** Moderation Analysis Results **eMethods 6.** Mediation Analysis: Further Detail **eTable 6.** Mediation Effects of SlowMo on GPTS: Mediator Variables at 12 Weeks and GPTS at 12 and 24 Weeks **eTable 7.** Mediation Effects of SlowMo: Mediator Variables at 12 Weeks and PSYRATS at 12 and 24 Weeks **eTable 8.** Mediation Effects of SlowMo: Mediator Variables at 12 Weeks and R-GPTS at 12 and 24 Weeks **eMethods 7.** Adverse Events **eTable 9.** Adverse Events and Trial-Related Adverse Events **eMethods 8.** Compliance Analysis **eTable 10.** Analysis of Treatment Received **eMethods 9.** Concomitant Therapy and Medication and Service Use Data **eTable 11.** Concomitant Therapy and Medication **eTable 12.** Service Use Over Preceding 6 Months at Baseline and Follow-up References.

Supplement 3. Data Sharing Statement
