# Supplementary material for: Effects of SlowMo, a Blended Digital Therapy Targeting Reasoning, on Paranoia Among People With Psychosis: A Randomized Clinical Trial
Source: JAMA Psychiatry. 2021 Apr 7;78(7):1–13. doi: 10.1001/jamapsychiatry.2021.0326 (PMC8027943; doi:10.1001/jamapsychiatry.2021.0326)
Supplement: Supplement 1. — Trial Protocol [file jamapsychiatry-e210326-s001.pdf]

SlowMo Trial Protocol and Statistical Analysis Plan (SAP)

1. SlowMo Trial Protocol

The following protocol (v1.0; 26/09/2016) predates the start of recruitment on the trial (01/05/2017). The trial protocol was also published in a peer reviewed journal:

Garety PA, Ward T, Freeman D, et al. SlowMo, a digital therapy targeting reasoning in paranoia, versus treatment as usual in the treatment of people who fear harm from others: study protocol for a randomised controlled trial. *Trials.* 2017;18(1):510.

Before and during the trial the only changes to this v1.0 protocol are as follows (resulting in a final Protocol Version 1.2):

R

**Document ID - (Document Title) revision X.Y**	**Description of changes from previous revision**	**Effective Date**
Version 1.1 ; 13/03/2017<br>Amended on 25/09/2018	Updated to reflect six-month trial extension	25/09/2018
Version 1.0; 26/09/2016- Amended on 13/3/2017	Inclusion criteria added (18 years old and use of SCAN). Further detail on screening and stratification by paranoia severity. Time-points specified on Table 1 (previously missing.)	13/3/2017

1. PROTOCOL FULL TITLE: The SlowMo Trial: A randomised controlled trial of a digital therapy for people who fear harm from others

Protocol Short Title/ Acronym:

SlowMo trial: a digital therapy for people who fear harm from others.

Trial Identifiers

**ISRCTN:**	32448671		
**REC Number:**	REC Reference: 16/LO/1862; IRAS: 206680.		
**UKCRN Number:**	**CPMS ID: 32154**		
**Protocol Version Number:**	1.0	Date:	26/09/2016

(Co) Sponsor(s)

**Name:**	Keith Brennan- Director of Research Management, Director of Administration (Health Schools)
**Address:**	Room 1.8 Hodgkin Building, Guy's Campus, King's College London, London SE1 4UL
**Telephone:**	020 7848 6960
**Fax:**	
**Email:**	keith.brennan@kcl.ac.uk

**Name:**	South London and Maudsley NHS Foundation Trust (Jennifer Liebscher)
**Address:**	R&D Department, Room W1.11, Institute of Psychiatry, Psychology & Neuroscience (IoPPN), De Crespigny Park, London, SE5 8AF
**Telephone:**	020 7848 0251
**Fax:**	
**Email:**	jennifer.liebscher@kcl.ac.uk

Chief Investigator

**Name:**	Professor Philippa Garety
**Address:**	PO Box 77 Henry Wellcome Building, SE5 8AF
**Telephone:**	+44 (0)20 7848 5046/ +44(0)7837 005412
**Fax:**	+44 (0)20 7848 5006
**Email:**	philippa.garety@kcl.ac.uk

Name and address of Co-Investigator(s), Statistician, Therapy Service, Laboratories etc

**Name:**	Professor Paul Bebbington
**Position/ Role:**	Emeritus Professor of Social and Community Psychiatry (Member of Research Team)
**Address:**	UCL Mental Health Sciences Unit, 2nd Floor, Charles Bell House, 67-73 Riding House Street
**Telephone:**	02076799465
**Fax:**	
**Email:**	P.Bebbington@ucl.ac.uk

**Name:**	Professor Graham Dunn
**Position/ Role:**	Professor of medical statistics (Trial Statistician)
**Address:**	University of Manchester, Centre for Biostatistics, Jean McFarlane Building (First Floor), Oxford Road, Manchester, M139PL
**Telephone:**	0161 275 5422
**Fax:**	
**Email:**	Graham.dunn@manchester.ac.uk

**Name:**	Professor Richard Emsley
**Position/ Role:**	Professor of medical statistics (Trial Statistician)
**Address:**	University of Manchester, Centre for Biostatistics, Jean McFarlane Building (First Floor), Oxford Road, Manchester, M139PL
**Telephone:**	0161 275 5422
**Fax:**	
**Email:**	richard.emsley@manchester.ac.uk

**Name:**	Professor David Fowler
**Position/ Role:**	Professor of Clinical Psychology (Member of Research Team)
**Address:**	University of Sussex, School of Psychology, Pevensey Building, University of Sussex, Falmer, BN1 9QH

**Telephone:**	01273 872721
**Fax:**	
**Email:**	d.fowler@sussex.ac.uk

32

**Name:**	Professor Daniel Freeman
**Position/ Role:**	Professor of Clinical Psychology (Member of Research Team)
**Address:**	University of Oxford, Department of Psychiatry, Warneford Hospital, Oxford, OX3 7JX
**Telephone:**	01865226490
**Fax:**	
**Email:**	daniel.freeman@psych.ox.ac.uk

33

**Name:**	Dr Kathryn Greenwood
**Position/ Role:**	Senior Research Fellow (Sussex University) and Consultant Clinical Psychologist (Sussex Partnership Trust, Research and Development) (Member of Research Team)
**Address:**	University of Sussex, School of Psychology, Pevensey Building, University of Sussex, Falmer, BN1 9QH
**Telephone:**	01273 872721
**Fax:**	
**Email:**	k.e.greenwood@sussex.ac.uk

34

**Name:**	Dr Helen Harding (nee Waller)
**Position/ Role:**	Research Clinical Psychologist (Member of Research Team)
**Address:**	South London and Maudsley Foundation NHS trust.
**Telephone:**	
**Fax:**	
**Email:**	helen.waller@kcl.ac.uk

35

**Name:**	Dr Amy Hardy
**Position/ Role:**	Research Clinical Psychologist (Member of Research Team); Digital Lead
**Address:**	PO Box 77 Henry Wellcome Building, SE5 8AF
**Telephone:**	02078485178

**Fax:**	
**Email:**	Amy.hardy@kcl.ac.uk

**Name:**	Professor Elizabeth Kuipers
**Position/ Role:**	Professor of Clinical Psychology (Member of Research Team)
**Address:**	PO Box 77 Henry Wellcome Building, SE5 8AF
**Telephone:**	02078480232
**Fax:**	
**Email:**	elizabeth.kuipers@kcl.ac.uk

**Name:**	Dr Tom Ward
**Position/ Role:**	Research Clinical Psychologist (Member of Research Team); Trial Co-ordinator
**Address:**	PO Box 77 Henry Wellcome Building, SE5 8AF
**Telephone:**	02078480594
**Fax:**	
**Email:**	Thomas.ward@kcl.ac.uk

2. Study Synopsis

**TITLE OF CLINICAL TRIAL:**	THE SLOWMO TRIAL: A RANDOMISED CONTROLLED TRIAL OF A DIGITAL THERAPY FOR PEOPLE WHO FEAR HARM FROM OTHERS.
**Protocol Short Title/ Acronym:**	SlowMo trial: a digital therapy for people who fear harm from others.
**Study Phase If Not Mentioned In Title:**	This is a late phase II/early phase III trial.
**Sponsor Name:**	Kings College London (Co-Sponsor: South London and Maudsley NHS Foundation Trust)
**Chief Investigator:**	Professor Philippa Garety
**UKCRN Number:**	**CPMS ID: 32154**
**REC Number:**	REC Reference: 16/LO/1862; IRAS: 206680.
**Medical Condition Or Disease Under Investigation:**	Psychosis (specifically paranoia/ fears about harm from others)

**Purpose Of Clinical Trial:**	We aim to test the clinical efficacy of SlowMo, our new therapy, and determine the mechanism through which it reduces paranoia severity, over 24 weeks, and to identify participant characteristics that moderate its effectiveness (either by moderating the degree of change in the mechanism, or by influencing adherence to the intervention).
**Primary Objective:**	The main research questions are as follows:<br>1. Is SlowMo efficacious in reducing paranoia severity over 24 weeks, when added to treatment as usual (TAU), in comparison to TAU alone?<br>2. Does SlowMo reduce paranoia severity by improving fast thinking (reducing belief inflexibility and jumping to conclusions)?<br>3. Do participant characteristics (i.e. their cognitive capacities, specifically working memory and thinking habits; and their symptoms, specifically negative symptoms) moderate the effects of the intervention?<br>4. Does outcome differ by adherence to the intervention and is adherence predicted by the participants' beliefs about their illness and about the intervention?<br>5. Does the SlowMo digital therapy platform have acceptable rates of usability, acceptability and adherence?<br>6. Does SlowMo reduce worry?
**Secondary Objective(s):**	N/A
**Trial Design:**	A parallel-group randomised controlled trial, with 1:1 allocation and blinded assessors, to test the efficacy of the SlowMo intervention in reducing paranoia severity when added to standardised Treatment As Usual (TAU).
**Endpoints:**	Assessments will be made at baseline, after treatment at 12 weeks, and at 24-week follow-up. Trial aims to commence in January 2017 and will proceed for a total of 31 months.
**Sample Size:**	360 people (2 groups):<br>Intervention (SlowMo) plus Treatment as Usual (TAU); n=180<br>TAU only; n=180
**Summary Of Eligibility Criteria:**	Inclusion criteria: persistent (3+ months) distressing paranoia (Green Paranoid Thoughts Scale (GPTS; Green et al., 2008) score >29, persecutory subscale), diagnosis of schizophrenia-spectrum psychosis (F20-29, ICD 10: Present State Examination, version 10), capacity to provide informed consent, sufficient grasp of English to participate in informed consent process, assessments and interventions.<br>Exclusion criteria: Profound visual and/ or hearing impairment; inability to engage in the assessment procedure; currently in receipt of psychological therapy for paranoia; primary diagnosis of substance abuse disorder, personality disorder, organic syndrome or learning disability.
**Intervention (Description, frequency, details of delivery)**	SlowMo consists of eight individual, face-to-face sessions (delivered weekly on average), delivered by trained therapists, and assisted by a website with interactive stories and games. SlowMo supports people to

	find out how fast thinking habits can contribute to upsetting thoughts, and try out tips to learn what helps them slow down their thinking and cope with worries. Personalised session content is synchronised with a mobile app to support people to make use of strategies learnt in their daily life.
**Comparator Intervention:**	Treatment as usual (TAU) only:<br>N.B All participants will receive TAU. We define usual care with reference to best practice guidance, specifically NICE guidance on community mental health treatment for people with psychosis and the standards of community care required by the Care Quality Commission. Participation will not alter normal treatment decisions about medication and additional psychosocial interventions which remain the responsibility of the clinical team.
**Maximum Duration Of Treatment Of A Subject:**	Time taken to complete the 8 sessions- typically period between randomisation and 12-week follow up.
**Version And Date Of Final Protocol:**	Version 1.0; 26/09/2016
**Version And Date Of Protocol Amendments:**	

3. Revision History

Document ID - (Document Title) revision X.Y	Description of changes from previous revision	Effective Date

4. Protocol Contents

1. PROTOCOL FULL TITLE: THE SLOWMO TRIAL: A RANDOMISED CONTROLLED TRIAL OF A DIGITAL THERAPY FOR PEOPLE WHO FEAR HARM FROM OTHERS 3

2. STUDY SYNOPSIS 6

3. REVISION HISTORY 8

4. PROTOCOL CONTENTS 8

5. BACKGROUND & RATIONALE 11

6. TRIAL OBJECTIVES AND DESIGN 12

6.1 TRIAL OBJECTIVES 12
6.2 FOLLOW-UPS/ ENDPOINTS 12
6.3 TRIAL DESIGN 12
6.4 TRIAL FLOWCHART 13

7. TRIAL INTERVENTION 13

7.1 THERAPY/INTERVENTION DETAILS 13
7.2 FREQUENCY AND DURATION OF INTERVENTION 14
7.3 INTERVENTION RECORDS 15
7.4 SUBJECT COMPLIANCE. 15
7.5 STUDY ADHERENCE 15
7.6 CONCOMITANT MEDICATION 15
8. RESEARCH ENVIRONMENT 15
9. SELECTION AND WITHDRAWAL OF SUBJECTS 16
9.1 INCLUSION CRITERIA 16
9.2 EXCLUSION CRITERIA 16
9.3 SELECTION OF PARTICIPANTS 16
9.4 RANDOMISATION PROCEDURE / CODE BREAK 16
9.5 WITHDRAWAL OF SUBJECTS 16
9.6 EXPECTED DURATION OF TRIAL. 17
10. TRIAL PROCEDURES 19
10.1 BY VISIT 19
10.2 LABORATORY TESTS 20
11. ASSESSMENT OF EFFICACY 20
11.1 PRIMARY OUTCOME 20
11.2 SECONDARY OUTCOME 20
11.3 PROCEDURES FOR ASSESSING EFFICACY PARAMETERS 20
12. ASSESSMENT OF SAFETY 20
12.1 SPECIFICATION, TIMING AND RECORDING OF SAFETY PARAMETERS. 20
12.2 PROCEDURES FOR RECORDING AND REPORTING ADVERSE EVENTS 20
12.3 ADVERSE EVENTS THAT DO NOT REQUIRE REPORTING 21
12.4 STOPPING RULES 22
13. STATISTICS 22
13.1 SAMPLE SIZE 22
13.2 RANDOMISATION 22
13.3 ANALYSIS 22
14. TRIAL STEERING COMMITTEE 23
15. DATA MONITORING COMMITTEE 23
16. DIRECT ACCESS TO SOURCE DATA AND DOCUMENTS 23
17. ETHICS & REGULATORY APPROVALS 23
18. QUALITY ASSURANCE 24
19. DATA HANDLING 24
20. DATA MANAGEMENT 25
21. PUBLICATION POLICY 25
22. INSURANCE / INDEMNITY 25
23. FINANCIAL ASPECTS 25
24. SIGNATURES 26
25. REFERENCES 27
26. APPENDIXES 29

5. Background & Rationale

'Every day, I think they are following me and am terrified that they will kill me.'

'Ben' believes he is in danger. When someone looks at him in the street he decides he is under attack. He rushes home and avoids going out. People often experience distressing fears about other people intentionally causing harm, which is also known as paranoia (Freeman et al., 2005). Paranoia severity lies on a continuum, and can range from fleeting ideas that someone on the street might be laughing at us, to more elaborate and persistent beliefs (sometimes called persecutory delusions) such as that the secret services are trying to have us killed. Paranoia is one of the most common symptoms of schizophrenia-spectrum disorders and is associated with significant distress and disruption to the person's life. This results in increased use of services, including inpatient admissions and high costs to mental health care providers. Developing effective interventions for paranoia is therefore a clinical priority. NICE (2014) recommend cognitive-behavioural therapy for psychosis (CBTp), including paranoia. However, there are significant challenges to access, engagement, adherence and effectiveness (Freeman et al., 2013; Haddock et al., 2013). CBTp has relatively high training and delivery costs, which limits access, and even when available, people can struggle to understand, remember and apply strategies learnt during therapy. Recent meta-analytical studies of CBTp have found small- to medium-sized beneficial effects on paranoia, and a pressing target of research is therefore to improve outcomes (van der Gaag et al., 2014). Our new therapy, SlowMo aims to address these identified challenges, specifically in terms of improving the appeal, ease of use and clinical effectiveness for people who fear harm from others.

Our research group has adopted an interventionist causal approach to improving therapy effectiveness, which involves developing tailored interventions to target the specific mechanisms that research has shown to play a causal role in paranoia. These mechanisms include thinking habits, worry processes, negative self-beliefs, safety behaviours, and sleep dysfunction (Freeman, 2016). Interventions targeting each of these mechanisms are all anticipated to reduce paranoia severity, albeit it through different pathways, given the multifactorial causality of paranoia. For example, a recent randomised controlled trial of a brief intervention focused on worry processes demonstrated that reductions in this mechanism accounted for improvements in paranoia (Freeman et al, 2015). In contrast, SlowMo works by targeting a certain type of thinking habit, which can be considered *fast thinking* (Garety et al., 2015; Kahneman, 2011). Fast thinking is characterised by focusing on too little information ('jumping to conclusions') and belief inflexibility (high conviction in thoughts and a lack of consideration of alternative ideas), and has been robustly associated with paranoia (Garety et al, 2014; Dudley et al., 2015; McLean et al., 2016; So et al., 2012). When Ben feels in danger, he is sure of what is happening based on his instincts, does not look for more information or consider other possible ideas. SlowMo aims to help people like Ben by supporting them to notice their upsetting worries and fast thinking habits, and then provides tips to help them *slow down for a moment* to focus on new information and develop safer thoughts.

We have iteratively developed SlowMo over the past 10 years, and now have sufficient proof-of-concept, feasibility and acceptability evidence from four preliminary studies to test the intervention in a randomised controlled trial (Ross et al., 2009; Waller et al., 2011; Garety et al., 2014; Waller et al. 2015). In three randomised studies and one case series, we found that reductions in unhelpful fast thinking account for improvements in paranoia severity, and that the intervention is highly acceptable. Our pilot data indicate very promising large effects on paranoia severity.

SlowMo has been developed from a user-centred inclusive design approach, to address the challenges to therapy engagement and adherence for people with severe mental health problems. It consists of an easy to use and enjoyable digital interface, thereby harnessing the potential of technology for improving health-related outcomes and reducing costs, in line with the 'NHS Five Year Forward View' (Hollis et al, 2015; NHS England, 2014). Thoughts are visualised as bubbles, with different speeds, sizes and colours, to reflect different thinking habits, levels of distress and coping tips. This simple metaphor makes it easier for people to understand thoughts are transient, and that by using coping strategies we can modify them. An interactive digital interface assists the delivery of face-to-face sessions, which are synchronised with a mobile app for use in daily life. Our design approach was informed by the Design Council's (2005) double diamond method consisting of discover, define, develop and deliver phases. As an inclusive design project, stakeholders (service users, clinicians and researchers) were involved from the outset, with iterative interviews, observation of therapy sessions, and system mapping of service contexts. This led to the development of a design brief, followed by iterative concept generation and prototype testing with service users. Feasibility testing of SlowMo has been extremely positive, with people indicating they significantly prefer the digital interface to conventional therapy materials.

Given its established evidence base and comprehensive user-centred design, SlowMo is expected to be highly acceptable and to lead to clinically worthwhile gains, reducing paranoia distress, conviction and preoccupation, enhancing wellbeing, and improving quality of life. It is anticipated to reduce service use, including inpatient admissions for the duration of the trial assessment period. The data from this study will also add significantly to our understanding of psychological mechanisms and change processes in paranoia. We will test our hypothesis that changes in fast thinking mediate changes in paranoia severity. In line with our interventionist causal approach, worry is not hypothesised to be a mediator as it is not targeted in the SlowMo intervention, but any observed effects will be explored. As well as providing valuable information for treatment development, evidence of mechanisms of action will inform the theoretical understanding of paranoia in a way that may itself shape future therapeutic initiatives. In addition, we have preliminary evidence of modifiers of treatment effects that we will investigate further. We will examine whether characteristics of participants (including working memory and negative symptoms) moderate the effects of the intervention on fast thinking, and also the effect on treatment of receipt of an adequate dose of treatment and therapy adherence. Finally, the trial will be the first to examine the usability and adherence of digital therapies in a large sample of people affected by severe mental health difficulties. The findings therefore have the potential to inform future stratified medicine approaches, and the development of more targeted therapies.

6. Trial Objectives and Design

6.1 Trial Objectives

Aims

We aim to test the clinical efficacy of SlowMo and determine the mechanism through which it reduces paranoia severity, over 24 weeks, and to identify participant characteristics that moderate its effectiveness (either by moderating the degree of change in the mechanism, or by influencing adherence to the intervention).

The main research questions are as follows:

1. Is SlowMo efficacious in reducing paranoia severity over 24 weeks, when added to treatment as usual (TAU), in comparison to TAU alone?
2. Does SlowMo reduce paranoia severity by improving fast thinking (reducing belief inflexibility and jumping to conclusions)?
3. Do participant characteristics (i.e. their cognitive capacities, specifically working memory and thinking habits; and their symptoms, specifically negative symptoms) moderate the effects of the intervention?
4. Does outcome differ by adherence to the intervention and is adherence predicted by the participants' beliefs about their illness and about the intervention?
5. Does the SlowMo digital therapy platform have acceptable rates of usability, acceptability and adherence?
6. Does SlowMo reduce worry?

Hypotheses

Primary hypotheses:

1. The intervention will reduce paranoia severity over 24 weeks.
2. Fast thinking (belief inflexibility and jumping to conclusions) will improve in response to the intervention.
3. Reductions in fast thinking will mediate positive change in paranoia severity.

Secondary hypotheses:

4. Poorer working memory and more severe negative symptoms will negatively moderate treatment effects.
5. Therapy adherence will moderate the effects of treatment on outcome and adherence will be predicted by beliefs about mental health problems.
6. Worry will not mediate reductions in paranoia severity

6.2 Follow-ups/ endpoints

Outcomes will be assessed over 24 weeks (first follow-up occurs at 12 weeks).

6.3 Trial Design

Design: A parallel-group randomised controlled trial, with 1:1 allocation and blinded assessors, to test the efficacy of the SlowMo intervention in reducing paranoia severity when added to standardised Treatment As Usual (TAU).

Independent randomisation (King's Clinical Trials Unit) will use randomly varying permuted blocks, stratified by site and baseline paranoia severity. Research workers will be blind to therapy allocation, to facilitate completion of unbiased and objective assessments. Adherence to the blindness procedure will be supported by the research co-ordinator and therapists having responsibility for the randomisation process, and informing participants of randomisation outcome. Further, the blinding procedure will be explained to participants and they will be reminded not to inform research workers of therapy allocation. Breaks in blinding will be monitored and recorded.

6.4 Trial Flowchart

Please refer to Appendix 1 for trial/ recruitment flow-chart and Section 11.1 for details of assessment at each visit.

7. Trial Intervention

7.1 Therapy/Intervention Details

Intervention: SlowMo consists of eight individual, face-to-face sessions, delivered by trained therapists, assisted by a website with interactive stories and games (see Figure One for examples on the session content). SlowMo supports people to find out how fast thinking habits can contribute to upsetting thoughts, and try out tips to learn what helps them slow down their thinking and cope with worries. Personalised session content is synchronised with a mobile app to support people to make use of strategies learnt in their daily life (see Figure Two for examples of the app content). The first two sessions involve learning that worries about others and fast thinking are common, and developing an individualised understanding of the person's thoughts and thinking habits. The concepts of 'thinking fast' and 'thinking slow' are introduced. It is explained that everyone thinks fast at times, and this can be helpful although at other times thinking fast can mean we feel worried when we do not need to be. Participants learn that thinking slow can be helpful in dealing with stress and worries about other people. This key principle frames the remaining 6 sessions where people are supported to find out about and try out tips to *slow down for a moment*, such as the impact of mood and past experiences on paranoia.

Figure One. Examples of the website content to support delivery of face-to-face sessions.

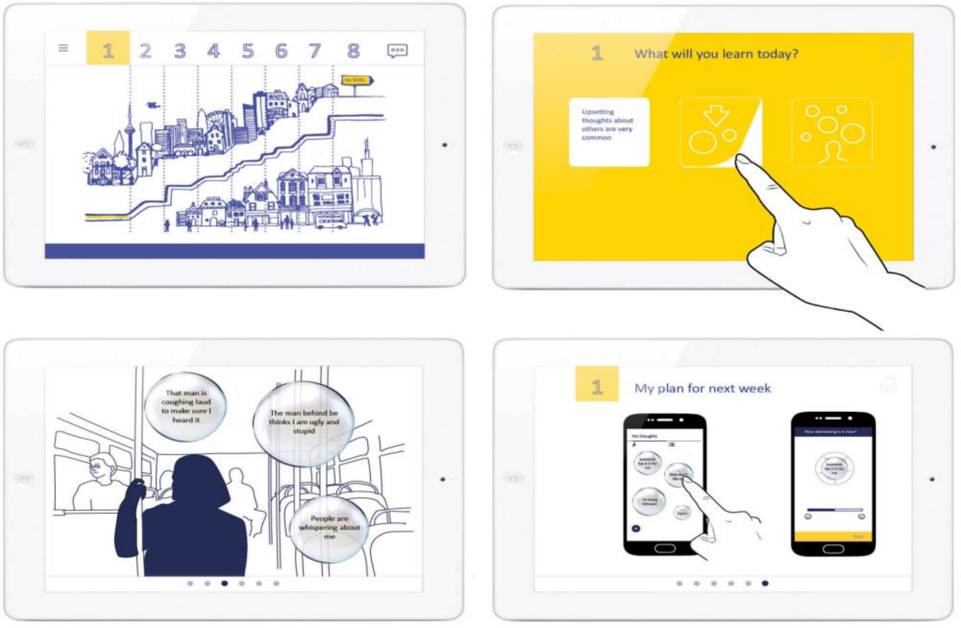

Figure Two. Examples of the app content to support self-management in daily life.

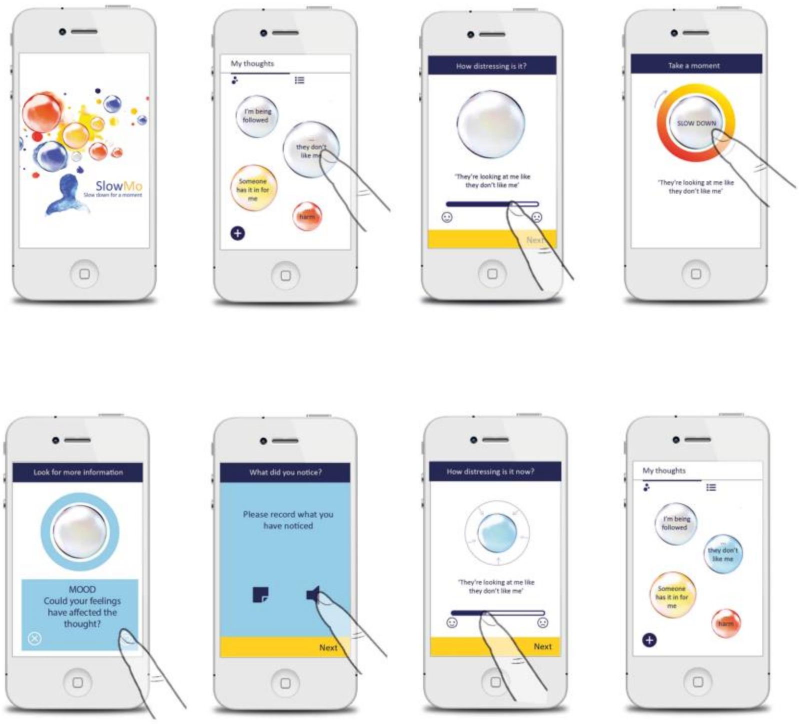

There is an emphasis throughout the intervention on practicing using the skills inside and outside of sessions. Participants build confidence in being able to manage paranoia, feeling safer in their daily life and working towards a valued goal. Security and privacy of information stored on the app has been considered throughout its development, with the functionality only allowing sharing of information with people's informed consent and no personally identifiable information being stored. If people agree, app usage can be synchronised with the digital platform and guides the subsequent sessions. The final session provides an opportunity for the participant to reflect on what has been learnt, progress made towards goals, and make plans for how they can continue to slow down their thinking and make use of coping tips in the future. The digital platform allows session-by-session monitoring of distress, conviction, preoccupation and general wellbeing which helps to monitor progress and tailor sessions according to participants' needs. Given the novelty of the digital platform therapy, usability and acceptability will be assessed through system analytics data on the use of the platform, a post-therapy assessment of participants' experience with a semi-structured interview and the User Experience Survey (adapted from Ben-Zeev et al, 2014) and a service-user led qualitative interview with a sub-sample of those receiving SlowMo (n = 20).

During the trial, therapy will be delivered by trained and experienced therapists, with expertise in working with this client group, who will attend peer supervision with the project team for the duration of the studies. The therapy will not interfere with the usual care offered through mental health services and no attempt to control the delivery of other services to either group will be made. The only exception to this will be if a person is currently receiving psychological interventions from another source, in which case we will liaise carefully with the participant and their therapist prior to randomisation to ensure that engagement in two psychological therapies is not overwhelming, confusing or unhelpful.

7.2 Frequency and duration of intervention

PROCEDURE: RECRUITMENT, INFORMED CONSENT AND RESEARCH ASSESSMENTS

Potential participants will be identified by close liaison between research workers and staff in clinical teams. Potential participants will be screened for suitability to see if they meet the initial eligibility criteria. Service users meeting these study criteria will be briefly introduced to the research by their clinician to see if they wish to give verbal consent to meet with the research worker and commence the remainder of the screening and informed consent process. Alternatively, potential participants may contact the researcher directly through responding to posters promoting the study displayed in community health team bases. If this is the case, the research worker will then complete the initial screen of the service user for suitability to participate, through discussion with the service user's clinician, before arranging to meet them to complete the screening process and commence the informed consent process. Potential

participants will be given the opportunity to discuss the study and at least 24 hours to decide whether to participate. The research worker will also assess capacity to provide consent to participate. Throughout the recruitment and research process all efforts will be made to tailor to participants' needs and preferences.

Service users who consent to participate will then complete a range of self-report and interview based measures involving questions about paranoia severity, wellbeing, self-esteem, quality of life, service use, worry and mood. Assessments will be done at baseline, after treatment at 12 weeks, and at 24-week follow-up. These assessments will be administered by trained local research workers, who will be supervised by experienced research clinical psychologists. Assessments will be conducted at locations convenient for the participant (at either NHS, University or residential locations). The research worker will inform the research coordinator when the baseline assessments have been completed, and the participant will then be randomised to either the SlowMo intervention or Treatment as Usual (TAU). The research coordinator or research therapist will meet with the participant to inform them of the outcome of randomisation and remind them about not informing the research worker of the allocation during the follow-up assessments. Participants will meet with the research workers again at 12 and 24 weeks following randomisation to complete follow-up assessments.

FOR PARTICIPANTS RANDOMISED TO TAU ONLY:

N.B All participants (in both groups) will receive TAU.

We define usual care with reference to best practice guidance, specifically NICE guidance on community mental health treatment for people with psychosis and the standards of community care required by the Care Quality Commission. Participation will not alter usual treatment decisions about medication and additional psychosocial interventions which remain the responsibility of the clinical team.

FOR PARTICIPANTS RANDOMISED TO SLOWMO IN ADDITION TO TAU:

SlowMo consists of eight individual, face-to-face sessions, delivered by trained therapists, assisted by a website with interactive stories and games. It is anticipated that face-to-face sessions will mostly be conducted at a local community clinical team setting. However the intervention is portable and therefore location can be changed in line with participant preference.

7.3 Intervention records

Assessments and therapy sessions will be audiotaped (after first establishing consent) to allow for assessment of adherence to the research protocol and assessment ratings. Relevant information concerning meetings with the project worker or therapist will be recorded in the participants' electronic notes system.

7.4 Subject Compliance.

Compliance will be determined by the participants' attendance at sessions and by system analytic data on engagement with the digital intervention.

7.5 Study adherence

Each session will be recorded and the following will be assessed:

1) Treatment adherence: sessions attended and system analytics data on website and app use.
2) Therapy adherence (including digital recording of in-session tasks and use of app for self-monitoring and exercises)
3) Therapist competence and fidelity to the manual.

7.6 Concomitant Medication

Participation will not alter usual treatment decisions about medication and additional interventions which remain the responsibility of the clinical team.

8. Research environment

The three main University trial sites are the Institute of Psychiatry, Psychology and Neuroscience, (King's College London), Oxford University and Sussex University. Participants will be recruited from mental health services associated with each University site with similar procedures followed at each site: South London and Maudsley NHS Foundation Trust, Sussex Partnership NHS Foundation Trust, and Oxford Health NHS Foundation Trust. Two additional PICs have been identified per site to be used as required: Oxford site- Berkshire Healthcare NHS Foundation Trust and Northamptonshire Healthcare NHS Foundation Trust; London site-South West London and St George's and Oxleas NHS Trust; Sussex site- Surrey & Borders Partnership NHS Foundation Trust and Kent & Medway NHS &

Social Care Partnership Trust. All measures and procedures, apart from therapy-specific assessments, will be administered by trained local research workers, who will be supervised by experienced research clinical psychologists. Assessments and therapy will be conducted at locations convenient for the participant (at either NHS, University or residential locations) and will be audiotaped to allow for reliability checks for adherence to the research protocol and assessment ratings. Please see Table One for an overview of the assessment battery.

9. Selection and Withdrawal of Subjects

9.1 Inclusion Criteria

Persistent (3+ months) distressing paranoia (Green Paranoid Thoughts Scale (GPTS; Green et al., 2008) score >29, persecutory subscale), diagnosis of schizophrenia-spectrum psychosis (F20-29, ICD 10: Present State Examination, version 10), capacity to provide informed consent, sufficient grasp of English to participate in informed consent process, assessments and interventions.

9.2 Exclusion Criteria

Profound visual and/ or hearing impairment; inability to engage in the assessment procedure; currently in receipt of other psychological therapy for paranoia; primary diagnosis of substance abuse disorder, personality disorder, organic syndrome or learning disability.

9.3 Selection of Participants

Recruitment: Participants will be recruited from mental health services across three main trial sites with similar procedures followed at each site: South London and Maudsley NHS Foundation Trust, Sussex Partnership NHS Foundation Trust, and Oxford Health NHS Foundation Trust. Participants will be identified through close liaison with clinical staff. Clinicians will need to obtain verbal consent from potential participants to be contacted by a study research worker, but no further demands will be placed on their time. After clinical staff have confirmed that a potential participant is suitable to be approached (i.e. meets study criteria and no clinical contra‒indications) Research Workers will meet each potential participant to discuss the study, provide written information, respond to questions and seek written informed consent.

Additional sources of recruitment:

1) Consent for Contact (C4C) provides access to existing research recruitment databases- e.g. South London and Maudsley (SLaM) Clinical Record Interactive Search (CRIS), an IT system which anonymises and provides authorised researchers with access to SLaM's 230,000 electronic health records. Sussex Partnership Trust is also currently setting up an opt-out system for consent to be contacted about research projects, scheduled to start in 2017, which should aid recruitment.

2) Patient Identification Centres (PIC) sites- two additional PICs have been identified per site to be used as required: Oxford site- Berkshire Healthcare NHS Foundation Trust and Northamptonshire Healthcare NHS Foundation Trust; London site-South West London and St George's and Oxleas NHS Trust; Sussex site- Surrey & Borders Partnership NHS Foundation Trust and Kent & Medway NHS & Social Care Partnership Trust.

3) Through direct patient approach: we intend to place recruitment posters in the main clinical areas of the specialist mental health teams. This will give details of the study. Although the poster asks participants to approach the research staff via their clinical team, we know from experience in the pilot that some will make a direct approach. Additional self-referrals are also possible as a result of interest generated through media/ public engagement events. In all such instances we will contact the relevant clinical team and discuss suitability for participation.

9.4 Randomisation Procedure / Code Break

Independent randomisation (King's Clinical Trials Unit) will use randomly varying permuted blocks, stratified by site and baseline paranoia severity.

9.5 Withdrawal of Subjects

Participants have the right to withdraw from the study at any time for any reason. The investigator also has the right to withdraw patients from the study in the event of clinical contra-indications. It is understood by all concerned that an excessive rate of withdrawals can render the study uninterpretable; therefore, unnecessary withdrawal of participants

should be avoided. Should a participant decide to withdraw from the study, all efforts will be made to report the reason for withdrawal as thoroughly as possible. Should a participant withdraw from therapy only, efforts will be made to continue to obtain follow-up data, with the permission of the patient.

9.6 Expected Duration of Trial.

The participation of each person within the trial will be 6 months from assessment/ randomisation until the 24 week follow-up.

Timescale

The study will take 31 months in 4 stages (with an additional preparatory stage 6 months

beforehand).

Stage 0 Preparatory stage in the six months before start: Detailed Trial Protocol drafted; Ethics and all R&D approvals applied for and granted; digital intervention and app redesign completed by end of May 2016; identification of trust therapists; initiation of coordinator recruitment; initiation of research worker recruitment; preparation of participant recruitment; computers and digital equipment ordered.

Milestone 1 Digital intervention and app re-design completed by end of May 2016

Stage 1 Months 1-3 Final set up: Staff recruitment completed and training completed, therapists trained and site-specific testing of digital platform completed. Participant recruitment initiated, including publicity campaign, visits to participating teams. Trial management folder and all essential trial documentation created; protocol finalised. Staff will be in post (trial and site coordinators on day 1 and research workers by the end of month 2 (all staff recruitment having commenced in preparatory phase). Trial therapists (previously identified) will be in place from start and will be trained in the first two months.

Milestone 2 Ethics and R&D approvals in place before start of month 1

Milestone 3 end month 3 Protocol submitted for publication.

Stage 2 Months 4-24 Participant recruitment and treatment delivery: Participant recruitment initiated, monitored and completed and treatment delivered without delay following randomisation. Data completion rates monitored. Participant recruitment (18 months: months 4-21) commences.

Milestone 4 end month 4: participant recruitment commenced in three sites

Milestone 5 end of month 6 Statistical Analysis Plan completed

Milestone 6 end month 7: 72 participants recruited, min 20 in each site. If Milestone 6 target not met in any site, activate additional recruitment sites in neighbouring Trusts

Milestone 7 end month 13: 192 participants recruited; 90 commenced treatment. If Milestone 7 not met, activate additional recruitment sites

Milestone 8 end month 21: 360 recruited (End of recruitment)

Milestone 9 end of month 24 All treatment completed

Stage 3 Months 25-27 Database completion and checking: All follow-up data collected. All baseline, 12 week and 24 week data correctly entered, checked, cleaned and data base locked ready for analysis

Milestone 10 end of month 27 All follow ups (24 weeks) completed; database fully checked, cleaned and locked.

Stage 4 Months 28-31 Final analysis and writing up. Data analysis, write up and initial dissemination. *Milestone 11 End of month 30 final report drafted.*

Project Gantt Chart:

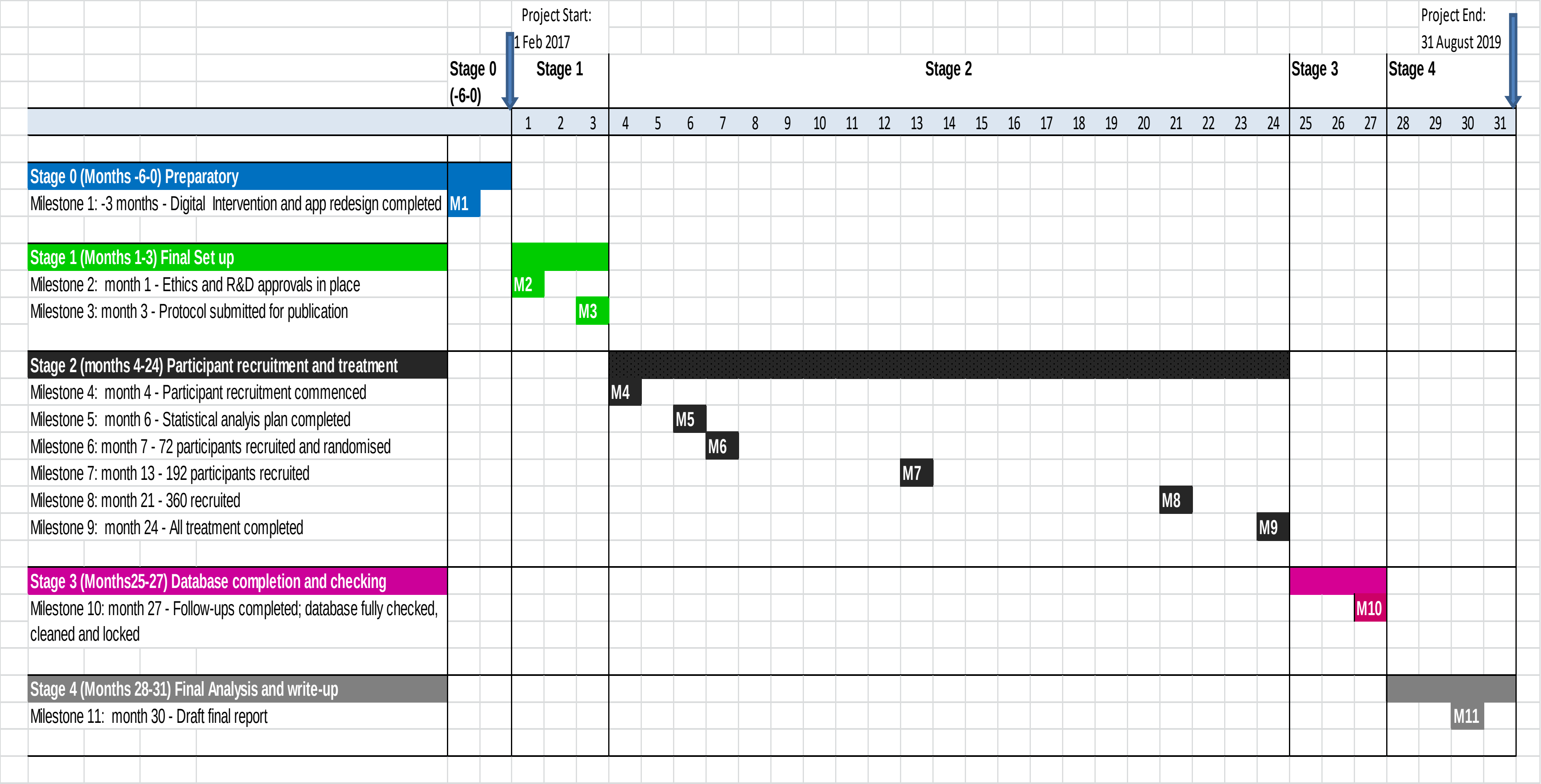

10. Trial Procedures

10.1 By Visit

Table One. Overview of assessment battery

Measure type	Measure	Time-point*
Paranoia screening for eligibility and primary outcome	Green Paranoid Thoughts Scale (GPTS). Green et al. (2008).	Screening,<br>1, 2, 3
Other paranoia outcome measures	The Psychotic Symptom Rating Scales (PSYRATS) – a dimensional measure of delusions. Haddock et al. (1999). Amended to include visual analogue scale ratings (0-100) of belief conviction, distress and preoccupation.<br>Persecutory delusions and ideas of reference items from Scales for Assessment of Positive Symptoms (SAPS). Andreasen (1984).	1, 2, 3
Fast thinking measures[1]	Maudsley Assessment of Delusional Beliefs (MADS): Possibility of Being Mistaken (PM). Wessely et al. (1993).	1, 2, 3
	Explanation for Experiences. Freeman et al. (2004).	1, 2, 3
	The Jumping to Conclusions Reasoning Test. Beads in ratios 60:40 and 85:15 Garety et al. (1991).	1, 2, 3
Other problems and processes	Scales for Assessment of Positive Symptoms (SAPS). Andreasen (1984).	1
	Brief Negative Symptom Scale (BNSS). Kilpatrick et al. (2011).	1
	Beliefs about Problems Questionnaire. Marcus et al. (2014).	1
	Letter Number Sequencing Test from the Wechsler Adult Intelligence Scale (WAIS). (Wechsler et al., 1997)	1
	Trail Making Task- A&B (Lezak 2004)	1
	TAPS (Thinking about Paranoia Scale); Hardy et al. (in prep)	1, 2, 3
	Penn State Worry Questionnaire (Meyer et al. 1990)	1, 2, 3
	Brief Core Schema Scales (BCSS). Fowler et al. (2006).	1, 2, 3
	Perception of carer criticism (adapted from	1

	Hooley et al., 1989)	
	The Warwick-Edinburgh Mental Well-being Scale (WEMWBS). Tennant et al. (2006).	1, 2, 3
	Short Assessment of Quality of Life (MANSA, Priebe et al 1999)	1, 2, 3
	Client Service Receipt Inventory including medication, bed and crisis team days, contact with criminal justice system. Beecham (1995).	1, 3

10.2 Laboratory Tests

N/A

11. Assessment of Efficacy

Participants will complete a range of self-report and interview based measures to assess the impact of the interventions on outcomes, the hypothesised mediators and other key processes implicated in paranoia and response to therapy (See Table 1 above for full details).

11.1 Primary outcome

The primary outcome is change in paranoia severity over 24 weeks.

11.2 Secondary outcome

Secondary outcomes include wellbeing, self-esteem, quality of life, service use, worry and standard mood and symptom assessments.

11.3 Procedures for Assessing Efficacy Parameters

N/A

12. Assessment of Safety

12.1 Specification, Timing and Recording of Safety Parameters.

Best practice, professional guideline and local NHS policies for monitoring mental state and risk will be followed throughout the participants' involvement in the trial and will be facilitated by close liaison with clinical teams.

12.2 Procedures for Recording and Reporting Adverse Events

Adverse Event (AE): Any untoward medical occurrence in a subject to whom a therapy has been administered including occurrences which are not necessarily caused by or related to that therapy.

Adverse Reaction (AR): Any untoward and unintended response in a subject to a therapy which is related to any duration of therapy administered to that subject.

Unexpected Adverse Reaction (UAR): An adverse reaction the nature and severity of which is not consistent with the information known about the therapy in question in the view of the investigator

Serious adverse Event (SAE), Serious Adverse Reaction (SAR) or Unexpected Serious Adverse Reaction (USAR): Any adverse event, adverse reaction or unexpected adverse reaction, respectively, that

- Results in death;
- Is life-threatening;

- Required hospitalisation or prolongation of existing hospitalisation;
- Results in persistent or significant disability or incapacity;
- Consists of a congenital anomaly or birth defect.

Reporting Responsibilities

All SARs and SUSARs (excepting those specified in this protocol as not requiring reporting) will be reported immediately by the Chief Investigator to the R&D office

Safety and adverse event assessment and monitoring: It is an important subsidiary goal of the trial to establish the safety of the intervention, and we will also take all appropriate steps during the conduct of the trial for ensuring participant safety. The occurrence of adverse events (AEs) will be monitored actively and systematically, following guidance from the Consolidated Standards of Reporting Trials (CONSORT) with the extension for non-pharmacologic treatment, and the extension for reporting of harms. Medical Research Council Guidelines for Good Practice in Clinical Trials will also be followed to ensure good governance of the trial for integrity and participants' safety and wellbeing. AEs are defined as including deaths; self-harm; serious violent incidents; complaints about therapy; referrals to crisis care or admission to psychiatric hospital during therapy. A standard method of reporting will be employed, categorising events by severity (five grades, A-E). Investigators will also determine relatedness of an event to the intervention based on a temporal relationship, as well as whether the event is unexpected or unexplained given the participant's clinical course, previous conditions and history, and concomitant treatments, in five categories from 'not related' to 'related' (following Linden 2013). The following will be considered as serious adverse events (SAE, Categories A-C): All deaths (category A), incidents which acutely jeopardise the health or psychological wellbeing of the individual, resulting in immediate hospital admission and/or permanent disability (category B), or resulting in injury requiring immediate medical attention (category C). These SAEs will include but are not limited to: 1) Hospital admissions; 2) Home treatment team involvement; 3) Suicide attempts; 4) Any violent incident necessitating police involvement (whether victim or accused); 5) Self-harming behaviour; 6) All deaths.

Reasons for withdrawal from the study will also be recorded. Furthermore, in the event of any AEs and participant withdrawal, the trial coordinator/ site coordinators will review participant clinical notes and contact clinicians for any important additional information. In order to ensure active surveillance of harms, at each assessment point, research workers will actively check for the occurrence of specific AEs using a structured checklist. At the completion of the trial, all medical notes will additionally be checked, for the total duration of enrolment, for any previously undisclosed record of AEs. This is to ensure completeness of records and to address the possibility that the disclosure of adverse events might be greater in the active intervention condition, as a result of the therapeutic relationship. For the final reports of the trial, the numbers, types and severity of AEs by trial condition, as well as discontinuations, will be reported, using descriptive statistics (since there are no pre-specified hypotheses concerning adverse events or harms, and, given the expected low frequency of AEs, the data will not be suitable for an ITT statistical analysis).

All SAEs will be reported immediately to the Chief Investigator and Principal Investigators (for each site) and the independent chair of the Data Monitoring and Ethics Committee (DMEC). All AEs including complaints (from each site) will be pooled and reported monthly to the Trial Management Committee and at each meeting of the DMEC. All relevant protocols for reporting SAEs to the Research Ethics Committee, the research sponsor and the respective local NHS Trust will be followed. Urgent actions concerning participant and staff safety, communication with others, and clinical care will be immediately addressed by the Trial CI and PIs and reported to the Trial Management Committee. At each meeting of the DMEC, or at any time at the request of the DMEC Chair, a full report of AEs will be reviewed. The DMEC will be responsible for investigating further, if there are any concerns about unexpectedly high rates of AEs, which may include being unblinded as to trial condition or seeking further data on adverse events, and will advise the TSC on any ethical or safety reasons why the trial should be prematurely ended.

12.3 Adverse events that do not require reporting

There are no AEs or SAES that do not require reporting for this trial.

12.4 Stopping Rules

The trial may be prematurely discontinued by the Sponsor or Chief Investigator on the basis of new safety information or for other reasons given by the Data Monitoring & Ethics Committee / Trial Steering Committee regulatory authority or ethics committee concerned. The trial may also be prematurely discontinued due to lack of recruitment or upon advice from a Trial Steering Committee (if applicable), who will advise on whether to continue or discontinue the study and make a recommendation to the sponsor. If the study is prematurely discontinued, active participants will be informed and no further participant data will be collected.

13. Statistics

Research workers will be blind to therapy allocation, to facilitate completion of unbiased and objective assessments. Adherence to the blindness procedure will be supported by the research co-ordinator and therapists having responsibility for the randomisation process, and informing participants of randomisation outcome. Further, the blinding procedure will be explained to participants and they will be reminded not to inform research workers of therapy allocation. Breaks in blinding will be monitored and recorded.

13.1 Sample Size

Total n=360 (120 per site):

SlowMO plus TAU; n=180

TAU only; n=180

Power calculation: Calculations used Clsampsi in Stata. A 10-point reduction in the primary outcome measure (GTPS) is clinically meaningful; based on a standard deviation of 25, this is a 0.4 effect size (Freeman et al, 2014). We account for: clustering in the SlowMo arm with an ICC=0.01 with 10 therapists (no clustering in the TAU arm), 1:1 allocation, 0.05 significance level. A simple two-tailed t-test with 150 people per group gives 90% power to detect an effect size of 0.4, and 80% for 0.35. In practice, power will be increased by using multiple regression. To allow for 20% attrition (conservatively high: our trials in this population had much lower rates: 5% Freeman et al, 2015; 4% Garety et al, 2008), we will recruit 360 patients at baseline split equally across 3 sites (120 per site, 60 per arm per site). While powering the study to detect moderate effect sizes, we anticipate larger effects: our sample is more homogeneous than in standard psychosis trials (being selected for one key problem: paranoia severity) with substantially less variance in the outcome variable and larger standardised effect sizes, giving increased power. For mediational analyses, N= 300 has >80% power to detect a proportion mediated of 40%, and >70% power to detect a proportion mediated of 30%, corresponding to findings in pilot work (calculated using PowerMediation in R).

13.2 Randomisation

Independent randomisation (King's Clinical Trials Unit) will use randomly varying permuted blocks, stratified by site and baseline paranoia severity.

13.3 Analysis

Analysis

Following CONSORT principles, we will report all participant flow and analyses will be conducted on the intention-to-treat (ITT) population: all participants randomised regardless of non-compliance with protocol or withdrawal from the study. Analyses will post-date final follow-up assessments, with due consideration of potential biases from loss to follow-up.

The primary analysis will test for a treatment effect on the primary and secondary clinical outcomes. Random effects regression models allowing for clustering by both participants and therapists will be fitted to the repeated measures, controlling for treatment site, baseline paranoia severity and the corresponding baseline assessment for the outcome under investigation. We will allow for missing outcome data under the Missing At Random assumption (Little and Rubin, 2002); we may also use inverse probability weighting to adjust for non-adherence to allocated treatment and other intermediate outcomes as predictors of future loss to follow-up (Dunn et al, 2005).

Secondary analyses will test treatment-effect mechanisms, moderation and process/adherence effects using modern causal inference methods (Emsley, Dunn & White, 2010, Dunn et al, 2015). The trial outcomes will comprise two parallel series of longitudinal data: one for the putative mediators (M) and one for the clinical outcomes (Y). For the mechanistic analysis, to test for a treatment effect on the putative mediator, we will replace the clinical outcome with the mechanistic variable as the dependent variable in the random effect models.

If we separately demonstrate a treatment effect on both the putative mediator and on the clinical outcome, we will evaluate mediation in these parallel longitudinal data sets through the use of parallel growth curve and latent change models (Cheong et al., 2003; MacKinnon, 2008). These models preserve the basic mediation model by replacing observed variables with latent constructs – the growth factors driving the temporal responses, M_1 to M_p and Y_1 to Y_p. Importantly the mediational structure only applies to the slope growth or change factors since randomised treatments are independent of the intercept growth factors (baseline values). Growth curve and latent change models can be estimated by maximum likelihood and other methods using the software package Mplus (Muthén & Muthén, 1998-2016). The application of these methods to mechanism evaluation within EME trials is illustrated in Dunn et al (2015), Chapter 4.

The aim of these analyses is to demonstrate that the effect of treatment on the growth (change) in the clinical outcome (Y) is explained (caused) by its effect on the growth (change) in the mediator. The major challenge to a valid inference is that there may be confounding of the mediator and outcome. We will begin by allowing for baseline values of the mediator and of the clinical outcome, as in the analyses of the successful WIT EME trial (Freeman et al, 2015) and then check the sensitivity of the results to the possibility of hidden confounding (unmeasured variables) through the use of instrumental variable methods (Emsley et al, 2010; Dunn et al, 2015).

14. Trial Steering Committee

The Trial Steering Committee (TSC) will meet at least annually and will report to the EME Programme. Its purpose is to provide overall supervision of the trial, approving the protocol and amendments, monitoring adherence to the protocol and providing independent advice on all aspects of the trial. Prof Richard Bentall, an independent international expert in psychological treatment research will be nominated as the chair. The TSC will include two further independent clinical academics, a service user and the lead investigator. Observers from the EME Programme will be invited to all TSC meetings.

15. Data Monitoring Committee

A DMEC will be convened and will meet at least annually and report to the TSC. It will have access to all trial data and will receive regular reports on adverse events. Membership of the DMEC will be independent of the applicants and of the TSC. Prof Andrew Gumley, an independent international expert experienced in conducting clinical trials with this population will be nominated as chair and the group will also comprise an independent senior statistician and another independent senior clinician. The DMEC will be notified of any serious adverse events as they occur and will consider whether any interim analyses are warranted, review data and advise the TSC on any ethical or safety reasons why the trial should be prematurely ended.

16. Direct Access to Source Data and Documents

The Investigator(s) will permit trial-related monitoring, audits and REC review by providing the Sponsor(s), and REC direct access to source data and other documents as required.

17. Ethics & Regulatory Approvals

The trial will be conducted in compliance with the principles of the Declaration of Helsinki (1996), the principles of GCP and in accordance with all applicable regulatory requirements including but not limited to the Research Governance Framework and the Mental Capacity Act 2005. This protocol and related documents will be submitted for review to Camberwell St Giles Research Ethics Committee (REC). The Chief Investigator will submit a final report at conclusion of the trial to the funder, the REC and the Sponsor.

18. Quality Assurance

The trial has been carefully designed to ensure compliance with Good Clinical Practice and scientific integrity. The research programme development, design and implementation will be managed by the Chief Investigator and the co-applicants, in consultation with service-user consultants and other expert research collaborators from within and outside of the Chief Investigator's institution. A dedicated Trial coordinator post will assist in the day-to day management of the project reporting to the Chief investigator, (CI). A trial management committee (TMC) will meet monthly, its membership will include the investigators and the Trial coordinator and site coordinators. It will be chaired by the CI and will manage the day–to-day running of the study and ensure good communication between trial sites, receiving monthly reports from each site on recruitment, therapy completion, adverse events, reviewing progress against milestones and finding solutions to problems as they arise. It will oversee the preparation of reports to the Trial Steering Committee (TSC) and Data Monitoring and Ethics Committee (DMEC). The Chief Investigator and the co-applicants are highly experienced in working clinically with service users with psychosis, and in carrying out research studies in this population.

19. Data Handling

The Chief Investigator will act as custodian for the trial data. The following guidelines will be strictly adhered to:

Participant data will be anonymised.

- All anonymised data will be stored on a password-protected computer.
- All trial data will be stored in line with the Data Protection Act.
- and archived in line with Sponsor requirements

CONFIDENTIALITY/ DATA PROTECTION

Issues relating to confidentiality will be addressed and potential participants will be advised of the limits of confidentiality (i.e. that the researcher will have a duty to inform health professionals if the participant discloses information which highlights any safeguarding or risk issues). The potential participant will be given at least 24 hours to consider all the information provided before written consent can be obtained. Participants will provide informed consent to data being collected on the understanding that information will be confidential and stored in a secure manner (in a locked room in a locked filing cabinet) for the duration of the study, or for longer, only if specific consent has been sought and given for this. A numerical system will be used for computerised information so that individual participants will not be identifiable. After completion of questionnaires and collection of demographic and clinical data, the researcher will destroy information linking participants to their research numbers so that individuals cannot be identified from their data. Participant consent forms will be retained, kept confidential and stored securely. All data will be destroyed following a period of 7 years as determined by relevant information governance policies) after the completion of the trial. It is possible that disclosure of criminal or other acts potentially requiring action will occur during sessions. The research team will be trained in both local and national policies for dealing with such disclosures, and have access to supervisory input to ensure appropriate action is taken. The possibility of action arising from certain disclosures will be clearly noted in the information sheet for participants.

PRIVACY ISSUES RELATED TO MOBILE APPLICATION (‘APP’)

We appreciate that use of a mobile application raises potential privacy issues, which we have considered throughout the development phase and are of great importance in mobile healthcare. We have developed the platform in line with the British Standards Institute quality criteria and code of practice for healthcare apps (2015) and guidance from the National Information Board. We have established and are regularly reviewing our risk management strategy and propose setting up a risk register that would be monitored by the trial management committee and data monitoring and ethics committee. Measures to address privacy issues include the informed consent process, which will ensure potential participants are fully aware of what data are collected by the platform, and how data are stored and used. This information will also be available from the

settings menu of the app, which consenting participants can access at any time. Second, all participants will have the opportunity, if they wish, to password protect the handset with a pin number or password. Third, the app does not store or transfer any personal identifiable information. Data transferred over internet transfer protocols will only contain a name (chosen by the person) and a Unique Device Identifier (UDID) which is generated automatically by the system, and will match the anonymised participant number. Any data transferred will also be secured by standard internet transfer protocol security layers. The welcome screen message does contain the participant's chosen name, should they agree to this doing so, however this can also be left blank if they prefer. During this project, the app will run as an offline native app, and therefore will not be connected to any network. App data will be synched during therapy sessions, over secure connections and stored on a password protected, secure database. It is of note that to date the app has been tested by service users with high levels of clinical paranoia, and all have wanted their name to be inputted onto the welcome screen.

AUDIO RECORDING

The study will adhere to the joint guidance on secure audio recording issued by King's College London and South London and Maudsley (SLaM). Assessment and therapy sessions will be recorded, with consent, using encrypted smart phone devices and data will be transferred to secure central storage as soon as possible. When not in use, devices will be stored in a locked cabinet within a locked office. Each device will be password protected. In the event of the device being lost or stolen this will be reported as a data incident to the Information Management and Compliance Team at King's College London and the Information Governance Team at SLaM. Any sensitive data on a lost/stolen device will be remotely erased.

20. Data Management

All data is anonymised at source. A log of contacts with participants including address and other contact details will be kept separate from all the research data. Details necessary to contact participants, and for communication with teams will be stored as above. Data will be shared through CRN, potentially with other researchers working under their auspices.

No patient identifiable information is recorded on the research assessment records and the computerised database is held centrally and managed by the KCL Clinical Trials Unit. Data from the assessments are entered into this central record by research assistants using a secure network connection. Audiorecording equipment will be used to record assessments to check fidelity to assessment protocols and allow for multiple ratings of assessments to ensure interrater reliability. The therapy sessions will be audio recorded (with participant consent) for monitoring the intervention in terms of fidelity and competence. These audio files named with a unique participant identifier will be stored as computer files on secure NHS/ University servers.

All personal data will be kept in a locked filing cabinet in a locked office at the three trial sites and will be accessible only by researchers. Therapy files will be kept in a secure office in the clinic and are not accessible to the staff collecting the research outcome data. Audio recordings of the therapy are stored as described above, are accessible to the patient's trial therapist and to the senior research clinician supervising that therapist.

21. Publication Policy

It is intended that the results of the study will be reported and disseminated at international conferences and in peer-reviewed scientific journals and will be made available to participants and clinical teams in an accessible format.

22. Insurance / Indemnity

KCL insurance applies.

23. Financial Aspects

This trial is fully funded by the MRC/NIHR Efficacy and Mechanism Evaluation (EME) Programme.

24. Signatures

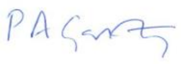

______________________________ 26.09.2016

Chief Investigator Date

Print name: Professor Philippa Garety

______________________________ ______________________

Statistician (if applicable) Date

Print name

25. References

Andreasen, N.C. (1984). *The scale for the assessment of positive symptoms (SAPS)*. Iowa City, Iowa: The University of lowa.

Beecham, J. (1995) Collecting and estimating costs. In M. Knapp: The Economic Evaluation of Mental Health Care. Aldershot: Arena.

Ben-Zeev D, Brenner CJ, Begale M et al. (2014) Feasibility, acceptability, and preliminary efficacy of a smartphone intervention for schizophrenia. *Schizophrenia Bull, 40,* 1244–53.

Boutron I, Moher D, Altman DG, Schulz KF, Ravaud P, Grp C. (2008) Extending the CONSORT statement to randomized trials of nonpharmacologic treatment: explanation and elaboration. Ann Intern Med. *148(4),* 295–309.

Cheong JW, MacKinnon DP, & Khoo ST (2003) Investigation of mediational processes using parallel process latent growth curve modelling, *Structural Equation Modeling, 10,* 238-62.

Design Council. (2005). Double Diamond Method. (2015, 10th June 2015). Retrieved from http://www.designcouncil.org.uk/sites/default/files/asset/document/ElevenLessons_Design_Council%20(2).pdf

Dudley, R., Taylor, P.J., Wickham, S., Hutton, P. (2015). Psychosis, delusions and the 'jumping to conclusions' reasoning bias: A systematic review and meta-analysis. *Schizophrenia Bulletin,* http://dx.doi.org/10.1093/schbul/sbv150

Dunn G., Emsley R., Liu H., Landau S., Green J., White I. & Pickles A. (2015) Evaluation and validation of social and psychological markers in randomised trials of complex interventions in mental health. *Health Technology Assessment, 19 (93). doi: 10.3310/hta19930*

Dunn, G. et al (2005). Estimating treatment effects from randomized clinical trials with noncompliance and loss to follow-up: the role of instrumental variable methods. *Statistical Methods in Medical Research, 14, 369-395.*

Emsley R., Dunn G., & White IR. (2010). Mediation and moderation of treatment effects in randomised controlled trials of complex interventions. *Stat Methods Med Res 19, 237–70.*

Freeman D. (2016). Persecutory delusions: a cognitive perspective on understanding and treatment, *The Lancet Psychiatry, 3(7),* 685-692.

Freeman, D., Dunn, G., Garety, P., Weinman, J., Kuipers, E., Fowler, D., Jolley, S. & Bebbington, P. (2013). Patients' beliefs about the causes, persistence and control of psychotic experiences predict take-up of effective cognitive behavior therapy for psychosis. *Psychological Medicine, 43(2),* 269-277.

Freeman, D., Dunn, G., Startup, H., Pugh, K., Cordwell, J., Mander, H., . . . Kingdon, D. (2015). Effects of cognitive behaviour therapy for worry on persecutory delusions in patients with psychosis (WIT): a parallel, single-blind, randomised controlled trial with a mediation analysis. *Lancet Psychiatry, 2*(4), 305-313.

Freeman, D., Garety, P. A., PE Bebbington, P. E. et al. (2005). Psychological investigation of the structure of paranoia in a non-clinical population, *Br J Psychiatry, 186,* 427–435

Freeman, D., Garety, P.A., Fowler, D., Kuipers, E., Bebbington, P. E., Dunn, G. (2004). Why do people with delusions fail to choose more realistic explanations for their experiences? An empirical investigation. *Journal of Consulting and Clinical Psychology, 72,* 671–680.

Fowler, D., Freeman, D., Smith,B., *et al*. (2006). The Brief Core Schema Scales (BCSS). Psychometric properties and associations with paranoia and grandiosity in non-clinical and psychosis samples. *Psychological Medicine, 36,* 749-759.

Garety, P. A., Hemsley, D. R., & Wessely, S. (1991). Reasoning in deluded schizophrenic and paranoid patients: Biases in performance on a probabilistic inference task. *Journal of Nervous and Mental Disease, 179,* 194-201.

Garety, P.A., Freeman, D., Jolley, S., Dunn, G., Bebbington, P.E., Fowler, D.G., Kuipers, E., Dudley, R. (2005). Reasoning, emotions and delusional conviction in psychosis. *Journal of Abnormal Psychology, 114,* 373-384.

Garety P, Waller H., Emsley R, Jolley S., Kuipers E., Bebbington P., Dunn G., Fowler D., Hardy A. & Freeman D. (2014) Cognitive mechanisms of change in delusions: An experimental investigation targeting reasoning to effect change in paranoia. *Schizophrenia Bulletin, 41 (2),* 400-410. doi: 10.1093/schbul/sbu103

Garety, P. A., Kuipers, E., Fowler, D., Freeman, D., Bebbington, P. E. (2001). A cognitive model of the positive symptoms of psychosis. *Psychological Medicine, 31.* 189-195.

Garety, P.A., Kuipers, E., Fowler, D., Freeman, D. & Bebbington, P.E. (2001). A cognitive model of the positive symptoms of psychosis. *Psychological Medicine*, *31*(2):189-195.

Garety, P. A., Fowler, D. G., Freeman, D., Bebbington, P., Dunn, G., & Kuipers, E. (2008). Cognitive-behavioural therapy and family intervention for relapse prevention and symptom reduction in psychosis: Randomised controlled trial. *British Journal of Psychiatry, 192,* 412-423.

Garety, P. A. & Freeman, D. (1999). Cognitive approaches to delusions: A critical review of theories and evidence. *British Journal of Clinical Psychology, 38, 113-154.*

Green, C. E., Freeman, D., Kuipers, E., Bebbington, P., Fowler, D., Dunn, G., Garety, P. A. (2008). Measuring ideas of persecution and social reference: The Green et al. Paranoid Thoughts Scales (GPTS). *Psychological Medicine, 38(1), 101-111.*

Haddock, G., Eisner, E., Boone, C., Davies, G., Coogan, C., & Barrowclough, C. (2014). An investigation of the implementation of NICE-recommended CBT interventions for people with schizophrenia. *Journal of Mental Health, 23(4),* 162-165.

Haddock, G., McCarron, J., Tarrier, N., & Faragher, E. B. (1999). Scales to measure dimensions of hallucinations and delusions: the psychotic symptom rating scales (PSYRATS). *Psychological Medicine, 29,* 879-889.

Hooley J. M & Teasdale J. D. (1989) Predictors of relapse in unipolar depressives: expressed emotion, marital distress, and perceived criticism. *Journal of Abnormal Psycholology, 98,* 229–35.

Hollis, C., Morriss, R., Martin, J., Amani, S., Cotton, R., Denis, M. & Lewis, S. (2014). Technological innovations in mental healthcare: harnessing the digital revolution. *The British Journal of Psychiatry, 206 (4),* 263-265.

Kahneman, D. (2011). *Thinking, fast and slow.* New York, NY: Farrar, Straus and Giroux; US.

Kilpatrick, B., Strauss, G.P., Nyugyen, L., Fischer, B.F., Daniel, D.C., Cienfuegos, S., Marder, S. R. (2011). The Brief Negative Symptom Scale: psychometric properties. *Schizophrenia Bulletin, 37,* 300-305.

Lezak MD, Howieson DB, Loring DW. *Neuropsychological Assessment*. 4th ed. New York: Oxford University Press; 2004.

Linden, M. (2013). How to define, find and classify side effects in psychotherapy: From unwanted events to adverse treatment reactions. *Clinical Psychology and Psychotherapy, 20,* 286-296.

Little, R.J.A. & Rubin, D.B (2002). Statistical Analysis with Missing Data (2nd Ed.). Wiley, Chichester & New York.

Marcus, E., Garety, P., Weinman, J., Emsley, R., Dunn, G., Bebbington, P. *et al.* (2014). A pilot validation of a modified Illness Perceptions Questionnaire designed to predict response to cognitive therapy for psychosis. *Journal of Behavior Therapy & Experimental Psychiatry, 45,* 459-466.

MacKinnon DP (2008). Statistical Mediation Analysis. New York: Lawrence Erlbaum Associates.

McLean, B. F., Mattiske, J. K. Balzan, R. P. (2016). Association of the Jumping to Conclusions and Evidence Integration Biases With Delusions in Psychosis: A Detailed Meta-analysis, Schizophrenia Bulletin, 11, Schizophrenia Bulletin Advance Access published May 11, 2016

Meyer TJ, Miller ML, Metzger RL, Borkovec TD (1990). Development and validation of the Penn State Worry Questionnaire. *Beh Research and Therapy, 28,* 487-495.

National Institute for Health and Care Excellence. Psychosis and schizophrenia: treatment and management. (Clinical guideline 178.) 2014. http://guidance.nice.org.uk/CG178].

Priebe, S., Huxley, P., Knight, S. & Evans, S. (1999) Application and results of the Manchester Short Assessment of Quality of Life (MANSA). *International Journal of Social Psychiatry*, 45, 7–12.

Ross, K., Freeman, D., Dunn, G. and Garety, P. (2009). A randomised experimental investigation of reasoning training for people with delusions. *Schizophrenia Bulletin, 37,* 324-333.

So, S. H., Freeman, D., Dunn, G., Kapur, S., Kuipers, E., Bebbington, P., Fowler, D., Garety, P. (2012). Jumping to Conclusions, a Lack of Belief Flexibilty and Delusional Conviction in Psychosis: A Longitudinal Investigation of the Structure, Frequency, and Relatedness of Reasoning Biases. *Journal of Abnormal Psychology, 121(1),* 129-139.

Tennant, R., Hiller, L., Fishwick, R., Platt, S., Joseph, S., Weich, S., Parkinson, J., Secker, J., Stewart-Brown, S. (2007). The Warwick-Edinburgh Mental Well-being Scale (WEMWBS): development and UK validation, *Health and Quality of Life Outcomes, 5(63).*

van der Gaag M., Valmaggia L. R &Smit F. (2014)The effects of individually tailored formulation-based cognitive behavioural therapy in auditory hallucinations and delusions: a meta-analysis. Schizophrenia Research, *156 (1),* 30-7. doi: 10.1016/j.schres.2014.03.016

Waller, H., Freeman, D., Jolley, S. & Garety, P. (2011). Targeting reasoning biases in delusions: A pilot study of the Maudsley Review Training Programme for individuals with persistent, high conviction delusions. *Journal of Behaviour Therapy and Experimental Psychiatry, 42,* 414-421.

Waller H., Emsley R., Freeman D., Bebbington P., Dunn G., Fowler D., Hardy A., Kuipers E. & Garety P. Thinking Well: A randomised controlled feasibility study of a new CBT therapy targeting reasoning biases in people with distressing persecutory delusional beliefs. *Journal of Behavior Therapy and Experimental Psychiatry*, 2015; 48: 82-9. doi: 10.1016/j.jbtep

Wessely, S., Buchanan, A., Reed, A., Cutting, J., Evertitt, B., Garety, P., & Taylor, P. J. (1993). Acting on delusions: I. Prevalence. *British Journal of Psychiatry, 163,* 69-76.

Wechsler D. Wechsler Adult Intelligence Scale–Fourth Edition. Pearson; San Antonio, TX: 2008.

World Health Organisation (2010). International Classification of Diseases 10th Revision (2010).

26. Appendixes

Appendix 1: SlowMo Trial
Design and Recruitment Flowchart

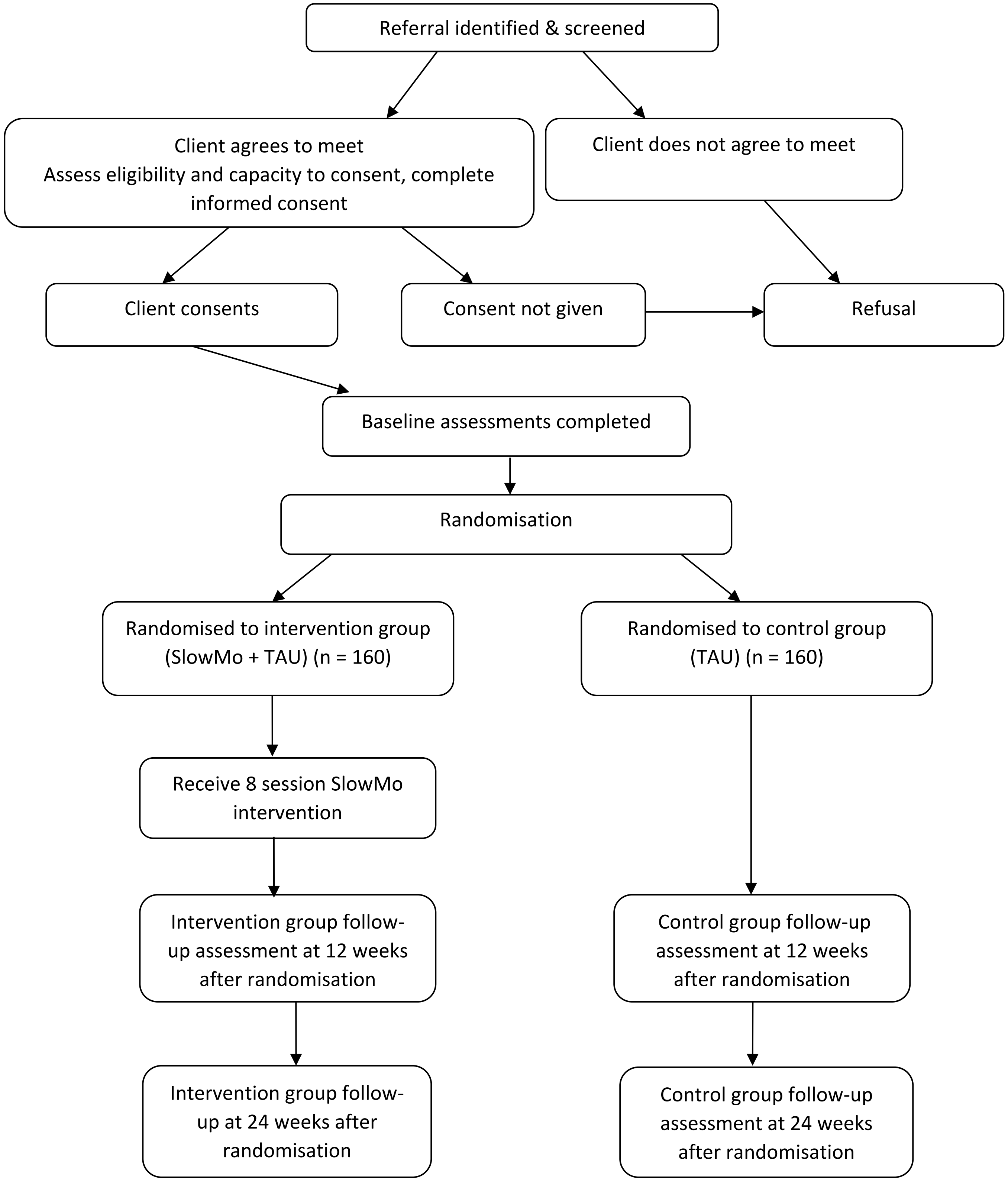

2. Statistical Analysis Plan (SAP)

Version: 1.2
Authors: Richard Emsley
Date: 20/12/2019
Protocol version: This SAP has been written based on Protocol V1.2
Trial registration: Current Controlled Trials ISRCTN32448671, registration date: 02/02/17.
Version history:

Version:	Date:	Changes:
1.2	20/12/19	Updated to include new version of GPTS based on Freeman et al 2019
1.1	27/11/2017	Incorporating comments from Investigator team
1.0	10/07/2017	First draft of SAP

Chief Investigator: Professor Philippa Garety

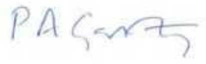

Date 28/01/20

Signature..

Trial Statisticians: Professor Richard Emsley

Signature Date 20/12/2019

Trial Steering Group Chair: Professor Richard Bentall

Signature...... 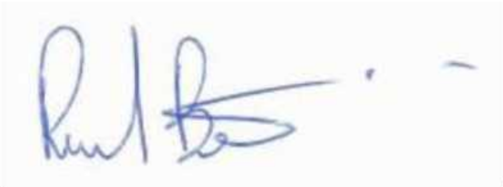 . Date . Feb 2nd 2020

DMEC Statistician: Professor John Norrie

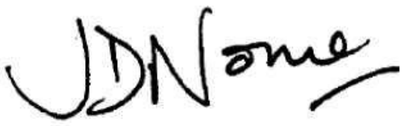

Signature .... Date 29/1/2020

Contents

1. Quantitative Analysis Plan 4
2. Research objectives and hypotheses 5
a. Research questions 5
b. Hypothesis 5
3. Brief description of the trial 6
a. Trial design 6
b. Randomisation procedure, allocation concealment and blinding 6
c. Eligibility screening 6
4. Outcome measures 7
a. Primary outcome 7
b. Secondary outcomes 7
c. Timing of outcome measurements 11
5. Sample size and power calculations 11
6. Data analysis - Data description 13
7. Statistical methods for inferential analysis 13
a. Primary outcomes 14
b. Secondary outcomes 14
c. Mediation analysis 14
d. Missing data 14
8. Database and data entry checks 15

1. Quantitative Analysis Plan

This document details the presentation and analysis strategy for the primary papers reporting results from the SlowMo trial. It is intended that the results reported in these papers will follow the strategy set out herein; subsequent papers of a more exploratory nature will not be bound by this analysis plan but will be expected to follow the broad principles laid down for the primary paper(s). The principles are not intended to curtail exploratory analysis or to prohibit sensible statistical and reporting practices but they are intended to establish the strategy that will be followed as closely as possible in analysing and reporting the trial. Reference was made to the trial protocol version 1.2 (dated 25/09/2018). A published protocol is available in (Garety et al, 2017, Trials 18:510.10.1186/s13063-017-2242-7), ICH guidelines on Statistical Principles (ICH E9 (1998)) reference and CONSORT SPI guidelines (Grant et al, 2018).

Investigators: Prof Philippa Garety, Prof Daniel Freeman, Prof David Fowler, Prof Richard Emsley, Prof Graham Dunn, Prof Elizabeth Kuipers, Prof Paul Bebbington, Prof Kathryn Greenwood, Dr Amy Hardy.

PI: Prof Philippa Garety

Trial Manager: Dr Tom Ward

Trial Statisticians: Prof Richard Emsley, Prof Graham Dunn

2. Research objectives and hypotheses

a. Research questions

- Is SlowMo efficacious in reducing paranoia severity over 24 weeks, when added to treatment as usual (TAU) in comparison to TAU alone?
- Does SlowMo reduce paranoia severity by modifying fast thinking (reducing belief inflexibility and jumping to conclusions)?
- Do participant characteristics (i.e. their cognitive capacities, specifically working memory and thinking habits; and negative symptoms) moderate the effects of the intervention?
- Does outcome differ by adherence to the intervention, and is adherence predicted by beliefs about illness and about the intervention?
- Does the SlowMo digital therapy platform have acceptable rates of usability, acceptability and adherence?
- Does SlowMo lead to changes in the following secondary outcomes: other delusional symptoms, wellbeing, quality of life, self and others schemas, service use and worry?

b. Hypothesis

Primary hypotheses:

1. The intervention will reduce paranoia severity over 24 weeks
2. Fast thinking (belief inflexibility and jumping to conclusions) will improve in response to the Intervention
3. Reductions in fast thinking will mediate improvement in paranoia severity

Secondary hypotheses:

- Poorer working memory and more severe negative symptoms will negatively moderate treatment effects
- Therapy adherence will moderate the effects of treatment on outcome and adherence will be predicted by beliefs about mental health problems
- Worry will not mediate reductions in paranoia severity

3. Brief description of the trial

a. Trial design

Parallel-group randomised controlled trial, with 1:1 allocation and blinded assessors, to test the efficacy of the SlowMo intervention in reducing paranoia severity when added to Treatment As Usual (TAU), compared with TAU.

b. Randomisation procedure, allocation concealment and blinding

Randomisation will take place via a web-based service hosted at the King's Clinical Trial Unit (KCTU). This can be accessed at www.ctu.co.uk by clicking 'randomisation - advanced' on the lower right hand side of the page. This system can only be accessed by trial staff who are trained and have previously been allocated a username and password. Requests for passwords are via the trial manager to the KCTU.

Independent randomisation will use an online system generating randomly varying permuted blocks, stratified by site and baseline paranoia severity. Stratification by paranoia severity will use a median split of >62 (Green Paranoid Thoughts Scale (GPTS) Part B).

Research workers will be blind to therapy allocation in order to facilitate completion of unbiased and objective assessments. Adherence to the blindness procedure will be supported by the research co-ordinator and therapists having responsibility for the randomisation process and for informing participants of randomisation outcome. Further, the blinding procedure will be explained to participants, who will be reminded not to inform research workers of therapy allocation. Breaks in blinding will be monitored and recorded.

c. Eligibility screening

Inclusion criteria:

- Aged 18 years and over;
- Persistent (3+ months) distressing paranoia (as assessed using the Schedules for Clinical Assessment in Neuropsychiatry (SCAN) and scoring >29 on the GPTS, Part B; persecutory subscale);
- Diagnosis of schizophrenia-spectrum psychosis (F20-29, ICD-10);
- Capacity to provide informed consent;
- Sufficient grasp of English to participate in informed consent process, assessments and interventions.

Exclusion criteria:

- Profound visual and/ or hearing impairment;
- Inability to engage in the assessment procedure;
- Currently in receipt of other psychological therapy for paranoia;
- Primary diagnosis of substance abuse disorder, personality disorder, organic syndrome or learning disability.

4. Outcome measures a.

Primary outcome

The primary outcome is paranoia severity measured by the GPTS over 24 weeks using both total score and subscale scores. The GPTS comprises two scales assessing thinking relevant to paranoia: ideas of social reference and persecution, rated over the preceding month. Each item is scored on a five-point Likert scale from 1 (‘not at all') to 5 (‘totally'). A total score can be calculated ranging from 32 to 160, with higher scores reflecting higher levels of paranoia. Two 16-item subscales assess ideas of social reference (Part A) and persecution (Part B) relevant to paranoia.

b. Other paranoia outcomes (as secondary outcomes)

- The Psychotic Symptom Rating Scales-Delusions (PSYRATS-Delusions), consisting of six items assessing the following dimensions of delusions: amount of preoccupation with delusions, duration of preoccupation with delusions, conviction, amount of distress, intensity of distress, and disruption to life caused by beliefs. Outcomes will be reported as total scores, as well as the two factors reported by Steel et al (2007): Factor 1 Preoccupation and Conviction; Factor 2 Distress.

- The persecutory delusions and ideas of reference items from the Scales for Assessment of Positive Symptoms (SAPS; Andreasen, 1984), a semi-structured interview designed to assess the positive symptoms of psychosis.

- The Revised-GPTS over 24 weeks using both total scores and subscale scores. The R-GPTS (Freeman et al, 2019) comprises two scales assessing thinking relevant to paranoia based on items from the original scale: ideas of social reference (8-items) and persecution (10- items), rated over the preceding month. Each item is scored on a five-point Likert scale from 0 (‘not at all') to 4 (‘totally'). A total score can be calculated ranging from 0 to 72, with higher scores reflecting higher levels of paranoia.

c. Mediators (as secondary outcomes)

Hypothesised mediators are measured by changes in fast thinking and slow thinking assessed by:

- Possibility of Being Mistaken (taken from the Maudsley Assessment of Delusions Schedule (MADS;); a binary measure (presence/ absence of flexibility) will be reported, together with self-reported % conviction in the ‘possibility of being mistaken'.
- Alternative Explanations from the Explanations of Experiences interview. A binary measure (presence/ absence of alternative explanations) will be reported, as well as the number of alternative explanations
- Jumping to Conclusions (JTC) Beads data-gathering task versions 85:15 and 60:40 A binary score will be constructed for presence (defined as fewer than 3 beads) JTC/ absence of JTC, as well as the number of beads drawn.

In addition to the above, we will report the level of conviction about the jar chosen in the beads gathering task (rated using a visual analogue scale from 0-100), together with the levels of endorsement (VAS 0-100) of rational reasoning (I took my time to think it through) and experiential reasoning (I chose based on a gut-feeling or hunch) to choose jar.

- The Fast and Slow Thinking Scale (FAST; Hardy et al. and previously named the TAPS) is a self-report questionnaire assessing dual-process reasoning in the context of paranoia. It comprises 10 statements rated on a 5-point scale (1 = not at all, 5 totally). There are two subscales, one assessing fast (intuitive) thinking and one measuring slow (analytic) thinking. Each subscale consists of 5 items, with higher scores reflecting greater endorsement of that reasoning style.

d. Other problems and processes (secondary outcomes)

- The Penn State Worry Questionnaire (PSWQ; Meyer et al., 1990) is a 16-item self-report measure of worry, with high internal consistency and good test-retest reliability (Meyer et al., 1990; Fresco et al., 2003). It uses a 5-point rating scale (1 = not at all typical of me, 5 = very typical of me) to assess worry. The total score is the sum of all responses; ranging from 16 to 80. Items 1, 3, 8, 11 are reverse-scored.
- The Brief Core Schema Scales (BCSS) is a self-report assessment of schemata concerning self and others in psychosis. It uses a 5-point scale (0 = do not believe it, 4 = believe it totally) and assesses four dimensions (each comprising 6 items) of self and other evaluation: negative-self, positive-self, negative-other, positive-other.
- The Warwick-Edinburgh Mental Wellbeing Scale (WEMWBS) comprises 14-items, designed to measure mental wellbeing over the last 2 weeks with a focus on positive aspects of mental health including positive affect (feelings of optimism, cheerfulness, relaxation), satisfying interpersonal relationships, and positive functioning (energy, clear thinking, selfacceptance, personal development, competence and autonomy). All items are positively worded, and are rated on a 5-point Likert scale ranging from 1 (‘none of the time') to 5 (‘all of the time'). Total scores ranges from 14 to 70, with higher scores indicating higher levels of well-being.

- The Manchester Short Assessment of Quality of Life (MANSA) is a 25-item measure assessing quality of life. The first Section (9 items) assesses demographic details (age at leaving full-time education,

employment status; kind of occupation; working hours per week; monthly income; state benefits; number of children; people the patient lives with; and type of residence). N.B we collect demographic information at the start of the assessment and will not report these items separately. Four subjective items assess the existence of a "close friend", whether a friend has been seen over the last week, whether the person has been accused of a crime (past year), and whether they have been the victim of physical violence (past year); these are answered dichotomously (yes or no) and summarised individually. Twelve subjective items assess satisfaction: with life as a whole, job, financial situation, number and quality of relationships, leisure activities, accommodation, personal safety, the people the patient lives with, sex life, family relationships, physical health, and mental health. These twelve satisfaction items are rated on a 7-point Likert scale ranging from 1 ('couldn't be worse') to 7 ('couldn't be better'). Total scores thus range from 12 to 84, with higher scores reflecting a better quality of life.

• Service Use is assessed by the Client Service Receipt Inventory (CSRI). We will report data in four domains (all over the last 6 months): Contact with professionals, Day service use, Home Treatment/ Crisis team involvement, Hospital Admission.

e. Adverse events

The occurrence of adverse events (AEs) will be monitored actively and systematically, following CONSORT guidance for reporting harms. AEs include: deaths; self-harm; serious violent incidents; complaints about therapy; and referrals to crisis care or admission to psychiatric hospital during therapy. A standard method of reporting will be employed, categorising events by severity (five grades, A-E). Subject to approval by the independent chairperson of the Data Monitoring and Ethics Committee, investigators will also determine whether an event is temporally related to the intervention, and whether it is unexpected or unexplained given the participant's clinical course, previous condition and history, and concomitant treatments. The event will then be rated in five categories from 'not related' to 'related'. Any associations between AEs and the SlowMo hardware or software will also be recorded.

f. Other measures (assessed at baseline only; potential moderators)

- Scales for Assessment of Positive Symptoms: a semi-structured interview assessing positive symptoms of psychosis in four domains: hallucinations, delusions, bizarre behaviour and positive formal thought disorder. Within the 4 domains a 6-point Likert scale is used to assess the severity of individual symptoms from '0' ("none") to '5' ("severe"). A scale total score is calculated by summing all individual items.

- Brief Negative Symptom Scale: a semi-structured interview designed to assess negative symptoms of psychosis. It comprises 13 items distributed over six domains: i) Anhedonia (Intensity and Frequency of pleasure (current activities); Intensity of expected pleasure for future activities); ii) Lack of normal distress (one item); iii) Asociality (Behaviour, Internal experience); iv) Avolition (Behaviour, Internal Experience); v) Blunted affect (Facial Expression, Vocal Expression, Expressive Gestures); and vi) Alogia (Quantity of speech and spontaneous elaboration). (The distress subscale has only one item). A likert scale ranging from absent (0) to severe (6) is used to assess the severity of each item. These individual scores are summed to provide a total score for the scale ranging from 0 to 78. Subscale scores are calculated by summing the individual items within each subscale.

- Beliefs about Problem Questionnaire: a 14-item self-report questionnaire designed to assess illness perceptions, including secondary appraisals of the nature, cause, duration, consequences and management of illness/problems. Each item is rated on a five-point likert scale in opinion format: Strongly agree=5, Agree= 4, Neither agree nor disagree= 3, Disagree= 2, Strongly Disagree=1. Items 5, 7 and 8 are reverse scored. Higher scores represent higher levels of cure-control, greater optimism and expectation of change, endorsement of psychological causes etc.)

- Letter Number Sequencing Test: A cognitive task which assesses working memory and involves sequencing, mental manipulation, attention, short-term auditory memory, visuospatial imaging and processing speed. In the task the participant is read a sequence of letter and numbers, and recalling the numbers in ascending order and letters in alphabetical order. The number is recalled first, followed by the letter, for

example, 3-W-5 would be 3-5-W and 1-J-A would be 1-A-J. The LNS includes a set of practice items, where corrections and repetitions can be made for errors in understanding the instructions. In the test phase of the LNS, instructions and items are not re-read to participants or corrected. Participants are deemed to have failed a test if they miss all 3 trials of an item. The task comprises 7 items, with 3 trials per item yielding an overall maximum raw score of 21 which is then converted into an age-adjusted Scaled Score.

- Trail Making Test: The Trail Making Test (TMT; Lezak, 2004) is an accessible and widely used neuropsychological instrument assessing a range of cognitive skills, such as visual attention, task switching, shifting cognitive set, psychomotor speed, abstraction, mental flexibility and executive function (Tombaugh, 2004; Salthouse, 2011). The TMT consists of two conditions: A and B, both requiring participants to draw lines sequentially connecting 25 numbers or letters as quickly as possible. In condition A, participants draw lines connecting circled numbers (1 - 25) in a numerical sequence, as fast as possible. In condition B, participants circle both numbers (1 - 13) and letters (A - L) in an alternating numerical and alphabetic sequence, as quickly as possible (e.g., 1-A-2-B-3-C-4-D-5). In both parts, participants are required to connect the set of circles as quickly and accurately as possible, without lifting the pen or pencil from the paper. Time taken in Task A and B will be reported.

- Perception of Carer Criticism. A single self-reported item adapted from Tooley et al (1989). This assesses the person's perception of criticism from a carer (where one is identified) over the previous month (How critical do you feel this person has been of you in the past month?). Scores range from 0 (not at all) to 5 (extremely).

g. Acceptability and adherence (in SlowMo therapy arm only)

The accessibility and usability of the therapy and the digital platform will be evaluated, along with adherence. Usability and acceptability of the digital platform will be assessed by the User Experience Survey, which consists of 12 items rated from 0 to 10 that comprise 3, 4-item subscales for enjoyment, ease-of-use and usefulness. This will be completed with therapist at the end of therapy.

Adherence to individual sessions will be assessed by number and duration of sessions attended, while the fidelity of therapy to the treatment manual will be assessed by the SlowMo therapy fidelity checklist completed by therapists at the end of each session. This will involve a) rating (dichotomous) on whether key webapp app **content** was delivered in each session and b) extent to which key SlowMo therapeutic **processes** were facilitated as per therapy manual (items vary by session with individual scoring ranging from 0=not at all; 1= partially; 2= totally). Adherence to the mobile app will be operationalised as at least one interaction with home-screen occurring outside of sessions for a minimum of three of the therapy sessions.

5. Timing of outcome measurements

A digital therapy for people who fear harm from others (SlowMo) STUDY PERIOD

	**Enrolment**	**Allocation**	**Post-allocation**		**Follow up**
**TIME POINT**	*Completed within 4 weeks*	*Within two weeks of baseline, 0 weeks*	*0-12 weeks*	*12-weeks*	*24-weeks*
**ENROLMENT:** ***Routine eligibility screen***	X				
Informed consent	X				
Allocation		X			
**INTERVENTIONS:** ***SlowMo+TAU***			X		
Treatment as usual (TAU)			X		
**ASSESSMENTS:** ***Primary outcome:***					
Paranoia severity (Green Paranoid Thoughts Scale, total, scale A and B)	X			X	X
Other paranoia outcomes:					
The Psychotic Symptoms Rating Scales- delusions	X			X	X
Delusions of persecution and reference items (Scales for Assessment of Positive Symptoms	X			X	X
Hypothesised mediators:					
Possibility of being mistaken (Maudsley Assessment of Delusions Schedule	X			X	X
Alternative Explanations (Explanations for Experiences	X			X	X
Jumping to Conclusions Reasoning	X			X	X
Other problems and processes:					
Scales for Assessment of Positive Symptoms	X				

Brief Negative Symptom Scale	X				
Beliefs about Problem Questionnaire	X				
Letter Number Sequencing Test	X				
Trail Making Test	X				
Fast and Slow Thinking (FAST, formally TAPS)	X			X	X
Penn State Worry Questionnaire	X			X	X
Brief Core Schema Scales	X			X	X
Perception of Carer Criticism	X				
The Warwick-Edinburgh Mental Wellbeing Scale	X			X	X
The Manchester Short Assessment of Quality of Life	X			X	X
Client Service Receipt Inventory	X				X

6. Sample size and power calculations

Our required sample size is N=300, based on the following considerations. We powered the study conservatively to detect a clinically meaningful 10-point reduction in the primary outcome measure (GTPS). Based on a standard deviation of 25, this is a 0.4 effect size. We account for: clustering in the SlowMo arm with an ICC=0.01 with 10 therapists (there is no clustering in the TAU arm), 1:1 allocation, 0.05 significance level. Calculations were made using Clsampsi in Stata. A simple twotailed t-test with 150 people per group gives 90% power to detect an effect size of 0.4, and 80% for 0.35. In practice, power will be increased by using multiple regression. In order to allow for a conservatively high 20% attrition, we will recruit 360 patients at baseline, split equally between 3 sites (120 per site, 60 per arm per site). For the mediation analyses, a sample of N=300 has >80% power to detect a proportion mediated of 40%, and >70% power to detect a proportion mediated of 30%, corresponding to findings in our pilot work (calculated using PowerMediation in R).

7. Data summary and reporting

We will report data in line with the Consolidated Standards of Reporting Trials (CONSORT) 2018 Statement for Social and Psychological Interventions showing attrition rates and loss to follow-up (see Figure 1). All analyses will be carried out using the intention to treat principle, incorporating data from all participants including those who do not complete therapy. Every effort will be made to follow up all participants in both arms for research assessments.

This statistical analysis plan will be agreed with a Data Monitoring and Ethics Committee before any inspection of post-randomisation data by the research team. No interim analysis is planned.

Analysis will be conducted in Stata version 15 or later. Descriptive statistics within each randomised group will be presented for baseline values. These will include counts and percentages for binary and categorical variables, and means and standard deviations, or medians with lower and upper quartiles, for continuous variables, along with minimum and maximum values and counts of missing values. There will be no tests of statistical significance or confidence intervals for differences between randomised groups on any baseline variable.

Descriptive statistics will be used to summarize assessments of feasibility and acceptability in terms of recruitment, drop-out and completeness of therapy.

Outcomes at 12 and 24 weeks will be presented separately for each group and summarised using counts and percentages for binary and categorical variables, and means and standard deviations for continuous variables, along with counts of missing values.

The number of serious adverse events and adverse events will be presented as the number of events and number of individuals with events. These will be provided separately for each randomised group and according to the treatment received.

8. Statistical methods for inferential analysis a.

Primary outcome

To test the primary hypothesis that the intervention will reduce paranoia severity over 24 weeks, we will fit a linear mixed model allowing for clustering by both participants and therapists to the repeated measures of GPTS. The model will include as fixed effects: randomised arm, time, time by randomised arm interaction, treatment site, baseline paranoia severity and the corresponding baseline assessment for the outcome under investigation. The treatment effect (between-group difference) will be extracted from the model for each time point. The model will include a random intercept for therapist in the intervention arm, with participants in the control arm considered as clusters of size 1. The use of a mixed effect models will allow for estimation of the intra-cluster correlation coefficient, a measure of the proportion of variance in outcome because of therapist effects.

b. Secondary outcomes

All secondary outcome measures (including putative mediators) will be analysed using the same modelling approach, using linear mixed models for continuous outcomes, and logistic mixed models for binary outcomes.

c. Moderation

The putative moderators will be tested separately by extending the mixed models by including as fixed effects the moderator, an interaction between moderator and treatment, and an interaction between moderator, time and treatment. The estimated coefficients from these will be used to assess if the treatment effects vary across levels of the moderator.

d. Mediation analysis

The trial outcomes will comprise two parallel series of longitudinal data: one for the putative mediators (M) and one for the clinical outcomes (Y). If we separately demonstrate a treatment effect on both the putative mediators and on the clinical outcomes, we will evaluate mediation in these parallel longitudinal data sets through the use of parallel growth curve and latent change models. These models preserve the basic mediation model by replacing observed variables with latent constructs - the growth factors driving the temporal responses, Ml to Mp and Y1 to Yp. Importantly the mediational structure only applies to the slope growth or change factors, since randomised treatments are independent of the intercept growth factors (baseline values).

Growth curve and latent change models will be estimated by maximum likelihood and other methods using Stata or latent variable modelling package Mplus. The aim of these analyses is to demonstrate that the effect of treatment on the growth (change) in the clinical outcome (Y) is explained (caused) by its effect on the growth (change) in the mediator. The major challenge to a valid inference is that there may be confounding of the mediator and outcome. We will begin by allowing for baseline values of the mediator and of the clinical outcome, as in the analyses of the successful EME Worry Intervention Trial. We will then check the sensitivity of the results to the possibility of hidden confounding (unmeasured variables) through the use of sensitivity analysis.

e. Missing data

Missing data on individual measures will be pro-rated if more than 90% of the items are completed; otherwise the measure will be considered as missing.

We will check for differential predictors of missing outcomes by comparing responders to nonresponders on key baseline variables. Any significant predictors will be included in the analysis models. This accounts for missing outcome data under a missing at random assumption, conditional on the covariates included in the model. As a sensitivity analysis, we will assess whether treatment adherence is associated with missing data, and if it is associated, use inverse probability weights or multiple imputation to compare results.

f. Presentation of results

Cohen's D effect sizes at 12 and 24 weeks will be calculated as the adjusted mean difference of the outcome divided by the sample standard deviation of the outcome at baseline. These will be displayed in a Forest Plot with the primary outcome at the top, all other paranoia outcomes below, followed by the mediators and the remaining secondary outcomes.

9. Database and data entry checks

Data quality will be ensured by close monitoring and routine auditing for accuracy throughout the data collection period. In order to ensure the accuracy of the data entered into the database, the main outcome measure entry will be checked for every participant by comparing the paper record with that on the database. An error rate of no more than 5% is acceptable. This will be done once all possible assessments for each time point have been completed. If the error rate is higher than 5%, advice will be sought from the trial statistician and methodologist regarding further data checking.

10. Draft figures and tables Figure 1:
Draft CONSORT statement

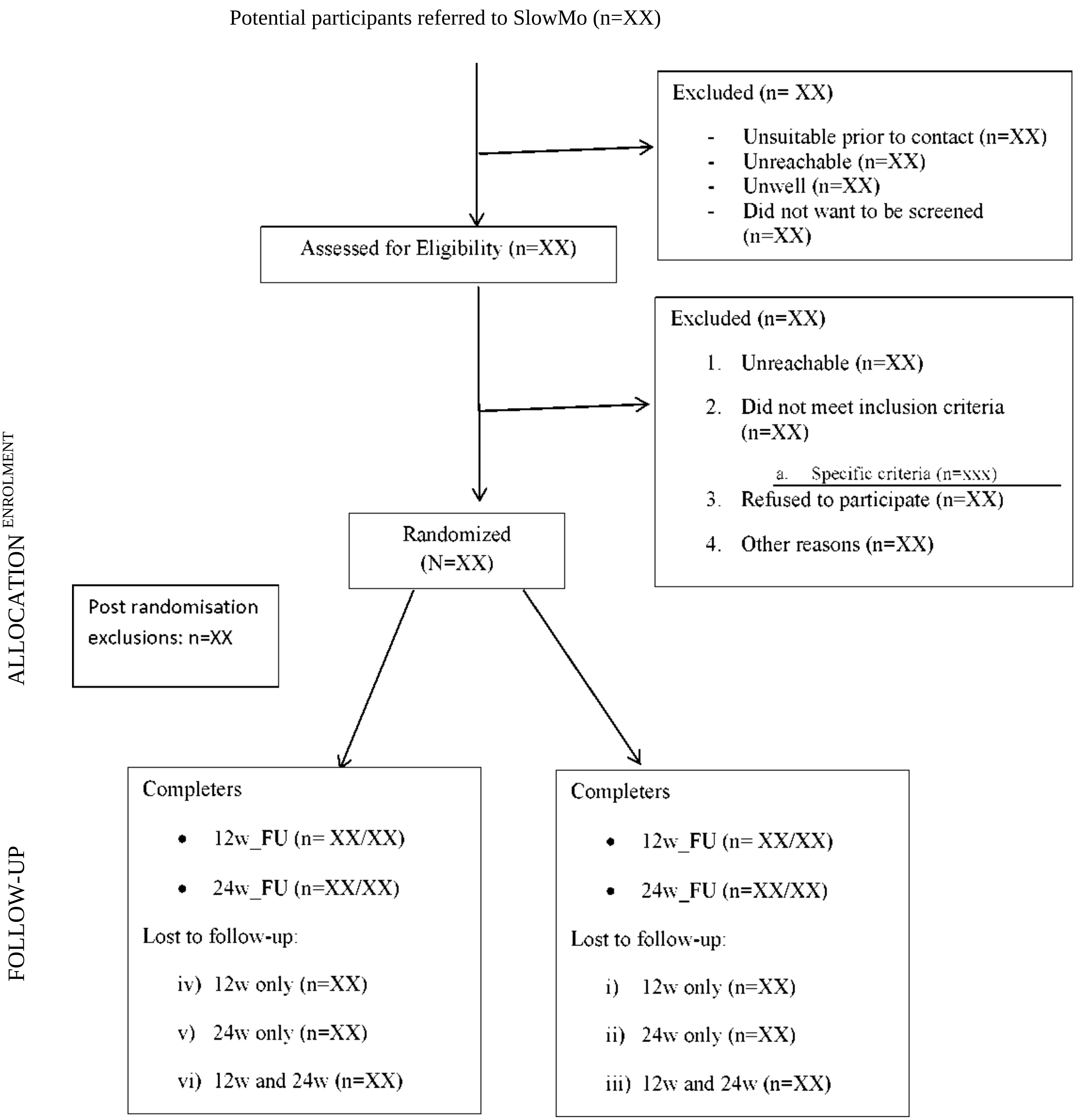

Analysed (n=)

Excluded from analysis (reasons) (n=)

Draft tables

Table 1 - Descriptive characteristics at baseline.

Baseline characteristics		Sample (N=)	SlowMo n=	TAU n=
Scale				
Sex N(%)	Male			
	Female			
Ethnicity N(%)	White			
N=	Black Caribbean			
	Black African			
	Black Other			
	Asian			
	Other			
Marital status N(%)	Single			
	Cohabiting			
	Married or civil partnership			
	Divorced			
	Widowed			
Highest level of schooling N(%)	Primary school			
	Secondary no exams qualifications			
	Secondary (O/ CSE equivalent)			
	Secondary (A level equivalent)			
	Vocational Education/ college			
	University degree/ professional qualification			
	Missing or not applicable			
Current working status N(%)	Unemployed			
	Employed full-time			
	Employed part-time			
	Self-employed			
	Retired			
	Student			
	Housewife/husband			
Normal living situation N(%)	Living alone (+/- children)			
	Living with partner			
	Living with parents			
	Living with other relatives			
	Living with others			

Table 2: Baseline Characteristics - Stratification factors

Baseline Characteristics		Total	SlowMo	TAU
Stratification Factor		N(%)		
Site	London			
	Oxford			
	Sussex			
Paranoia severity	GPTS Part B < 62			
	GPTS Part B > 62			

Table 3: Baseline clinical outcomes

Scale	Sample mean (SD) or N(%)	SlowMo	TAU
**Primary outcome**			
GPTS - total score			
GPTS (A) - ideas of social reference			
GPTS (B) - paranoia			
**Secondary outcomes - other paranoia scales**			
PSYRATS - Delusions			
SAPS - delusions item			
SAPS - ideas of reference item			
R-GPTS total score			
R-GPTS (A)			
R-GPTS (B)			
**Secondary outcomes - mediators**			
Possibility of being mistaken (yes)			
% conviction in Possibility of being mistaken			
Alternative explanations (yes)			
Jumping to conclusions 85:15 task (yes)			
Jumping to conclusions 85:15 task (Mean, SD)			
Jumping to conclusions 60:40 task (yes)			
Jumping to conclusions 60:40 task (Mean, SD)			
**Secondary outcomes - other problems**			
Penn State Worry Questionnaire			
Brief Core Schema Scales - negative self			
Brief Core Schema Scales - positive self			
Brief Core Schema Scales - negative other			
Brief Core Schema Scales - positive other			
FAST - fast thinking			
FAST - slow thinking			
WEMWBS			

Table 4: Primary outcome

	Unadjusted, Mean (SD)			
Outcome	SlowMo<br>*n*= XX	TAU<br>*n*= XX	Adjusted Difference (SE); p- value (95% CI)	Cohen *d*<br>*(95% CI)*
GPTS				
12 weeks				
24 weeks				
GPTS -A				
12 weeks				
24 weeks				
GPTS - B				
12 weeks				
24 weeks				

Table 5: Secondary outcomes

	Unadjusted, Mean (SD)			
Outcome	SlowMo<br>*n*= XX	TAU<br>*n*= XX	Adjusted Difference (SE); p-value (95% CI)	Cohen *d*<br>*(95% CI)*
Outcome				
12 weeks				
24 weeks				
