# Supplementary material for: Effects of SlowMo, a Blended Digital Therapy Targeting Reasoning, on Paranoia Among People With Psychosis: A Randomized Clinical Trial
Source: JAMA Psychiatry. 2021 Apr 7;78(7):1–13. doi: 10.1001/jamapsychiatry.2021.0326 (PMC8027943; doi:10.1001/jamapsychiatry.2021.0326)
Supplement: Supplement 2. — eMethods 1. SlowMo Therapy Description eFigure 1. SlowMo Webapp Formulation eMethods 2. SlowMo Mobile App Adherence Criterion eMethods 3. Data Quality and Interrater Reliability eMethods 4. Data Completeness and Timing of Assessments eTable 1. Unblinding by Site eTable 2. Number (Percentage) Above Threshold for a Potential Persecutory Delusion eFigure 2. Forest Plot Binary Outcomes eTable 3. Baseline Characteristics by Site eMethods 5. Moderation Analysis: Further Detail eTable 4. Baseline Clinical and Cognitive Characteristics as Potential Moderators of the Intention-to-Treat Population eTable 5. Moderation Analysis Results eMethods 6. Mediation Analysis: Further Detail eTable 6. Mediation Effects of SlowMo on GPTS: Mediator Variables at 12 Weeks and GPTS at 12 and 24 Weeks eTable 7. Mediation Effects of SlowMo: Mediator Variables at 12 Weeks and PSYRATS at 12 and 24 Weeks eTable 8. Mediation Effects of SlowMo: Mediator Variables at 12 Weeks and R-GPTS at 12 and 24 Weeks eMethods 7. Adverse Events eTable 9. Adverse Events and Trial-Related Adverse Events eMethods 8. Compliance Analysis eTable 10. Analysis of Treatment Received eMethods 9. Concomitant Therapy and Medication and Service Use Data eTable 11. Concomitant Therapy and Medication eTable 12. Service Use Over Preceding 6 Months at Baseline and Follow-up References. [file jamapsychiatry-e210326-s002.pdf]

Supplementary Online Content

Garety P, Ward T, Emsley R, et al. Effects of SlowMo, a Blended Digital Therapy Targeting Reasoning, on Paranoia Among People With Psychosis: A Randomized Clinical Trial. *JAMA Psychiatry*. Published online April 7, 2021. doi:10.1001/jamapsychiatry.2021.0326

eMethods 1. SlowMo Therapy Description

eFigure 1. SlowMo Webapp Formulation

eMethods 2. SlowMo Mobile App Adherence Criterion

eMethods 3. Data Quality and Interrater Reliability

eMethods 4. Data Completeness and Timing of Assessments

eTable 1. Unblinding by Site

eTable 2. Number (Percentage) Above Threshold for a Potential Persecutory Delusion

eFigure 2. Forest Plot Binary Outcomes

eTable 3. Baseline Characteristics by Site

eMethods 5. Moderation Analysis: Further Detail

eTable 4. Baseline Clinical and Cognitive Characteristics as Potential Moderators of the Intention-to-Treat Population

eTable 5. Moderation Analysis Results

eMethods 6. Mediation Analysis: Further Detail

eTable 6. Mediation Effects of SlowMo on GPTS: Mediator Variables at 12 Weeks and GPTS at 12 and 24 Weeks

eTable 7. Mediation Effects of SlowMo: Mediator Variables at 12 Weeks and PSYRATS at 12 and 24 Weeks

eTable 8. Mediation Effects of SlowMo: Mediator Variables at 12 Weeks and R-GPTS at 12 and 24 Weeks

eMethods 7. Adverse Events

eTable 9. Adverse Events and Trial-Related Adverse Events

eMethods 8. Compliance Analysis

eTable 10. Analysis of Treatment Received

eMethods 9. Concomitant Therapy and Medication and Service Use Data

eTable 11. Concomitant Therapy and Medication

eTable 12. Service Use Over Preceding 6 Months at Baseline and Follow-up

References.

This supplementary material has been provided by the authors to give readers additional information about their work.

©2021 Garety P et al. *JAMA Psychiatry*.

eMethods 1. SlowMo Therapy Description

Therapy structure

SlowMo is a digitally supported cognitive-behavioural therapy consisting of eight individual, face-to-face sessions, each module addressing a specific topic, typically lasting 60-90 minutes, within a 12-week timeframe. Therapy delivery is assisted by a web-based app (the webapp) delivered via a touchscreen laptop, with interactive features including information, animated vignettes, games and personalised content, which is synchronised with a native mobile app installed on a standard android smartphone provided to participants to assist therapy generalisation. Mobile app set up and use ('onboarding') is facilitated by the therapist at the end of the first session with ongoing support as needed (typically during an initial check-in at the start of each session).

The webapp structure is delivered consistently, but content is tailored throughout as participants interact with personalised worry 'bubbles', safer/positive thought 'bubbles', key learning and messages for the week ahead (recorded by the person at the end of each session in text or audio form and then synchronised with the mobile app). The mobile app allows people to notice their fears and thinking habits as they occur in daily life, and supports them to find other ways of managing distressing experiences.

Therapy content

Sessions 1 - 2 involve building the meta-cognitive skill of noticing thoughts and thinking habits (visualised as 'bubbles' spinning slowly or fast). People learn the normalising message that fast thinking (jumping to conclusions and belief inflexibility) is part of human nature and can be useful at times. However, fast thinking can fuel worries and thinking slowly can be helpful in dealing with difficult situations and fears about other people.

Formulation is commenced in session 1 and iteratively developed throughout therapy. A flexible approach ensures that the targeted causal processes (fast and slow thinking) are communicated in an individualised and accessible manner. The webapp formulation is presented in simple ABC format (My triggers; My upsetting thoughts; Impact on my life) to promote accessibility (See Figure 1). Where helpful, specific aspects of the formulation (e.g. specific reciprocal interactions between thoughts, mood, and safety behaviour) are drawn out by the therapist separately to the webapp. In addition to a consistent focus on 'in the moment' processing of threat, SlowMo therapy contextualises fear of harm from others within the broader context of the person's life and social relationships. In sessions 3 – 8 people are supported to try out ways to slow down for a moment, e.g. by considering the impact of mood and past experiences on concerns and by looking for safer alternative explanations.

Behavioural work is completed as an adjunct to webapp guided aspects of the therapy, with the aim of facilitating learning and includes: a) 'In vivo' assessment in relevant social contexts (early sessions) b) testing out use of SlowMo principles around slowing down for a moment in specified situation c) testing out specific mobile app functions (slowing down bubbles, use of tips and messages) and d) behavioural experiments testing out predictions about feared outcomes and/or outcome of using alternative strategies in situ (shifting focus of attention from threat, dropping safety behaviours etc).

©2021 Garety P et al. *JAMA Psychiatry*.

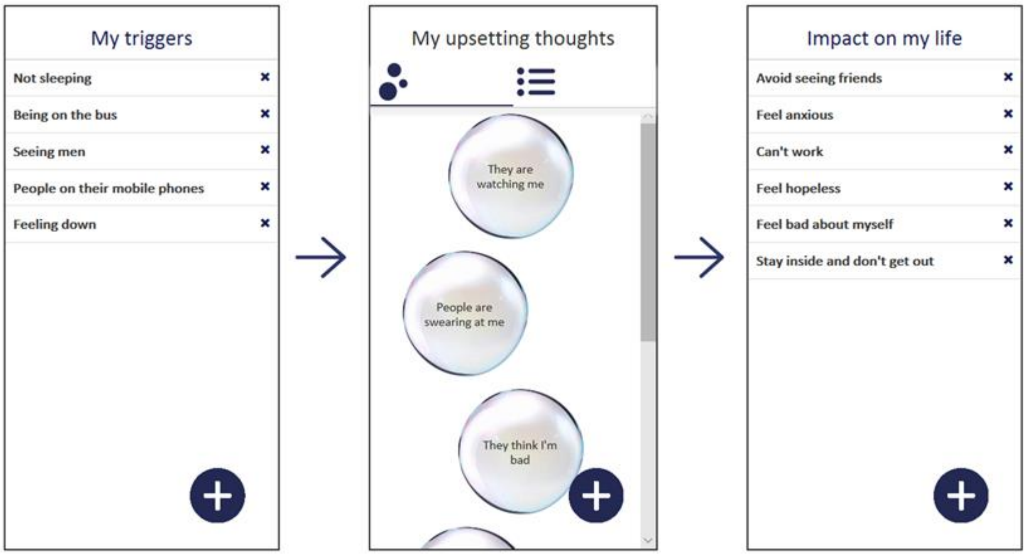

eFigure 1. SlowMo Webapp Formulation

eMethods 2. SlowMo Mobile App Adherence Criterion

Adherent use for the mobile app was operationalised as at least one homescreen interaction following a minimum of 3 of the therapy sessions. Mobile app analytics data were only collected for 7 of the 8 sessions as the app disconnected from analytics database at session 8, to ensure participants' privacy of usage after the end of therapy. Homescreen interactions were selected for the adherent use criterion as engagement with this screen is indicative of viewing content to support slowing down (i.e. personalised bubbles with participants' worries and ways of feeling safer) as well as being the screen through which other functions that assist slowing down are accessed (e.g. slowing down a specific thought, reviewing slowing down tips, personalised therapy messages, relaxation and distraction exercises). Our definition of mobile app adherence was based on a theoretical rationale, in line with best practice guidance.[1] The mobile app aims to support people to learn the skill of slowing down, and therefore we did not assume that frequent and prolonged use was necessary.[2,3] Instead, sufficient adherence was operationalised as use following some, but not all, of the sessions, with reductions in use hypothesised to potentially reflect e-attainment. A minimum of one interaction between sessions was set based on clinical experience that establishing any autonomous application of therapy to the real-world represents a significant challenge, but where this occurs it can facilitate clinically meaningful changes (e.g. reviewing slowing down skills to support participation in a personally valued activity).

eMethods 3. Data Quality and Interrater Reliability

Data quality was assessed by auditing the main outcome measure baseline entry for accuracy. An error rate of no more than 5% was deemed acceptable a priori. [4] Data quality was confirmed as good, with an observed error rate of 0.03%. Interrater reliability analysis was conducted on the main observer-rated measure of paranoia, the PSYRATS, and both observer-rated Belief flexibility Items (PM and AE) for 45 of the baseline assessments selected randomly (15 per site) from assessments conducted after an initial training and consensus period. The Intraclass Correlation (ICC) for the PSYRATS (absolute-agreement, 2-way mixed-effects model, single measures) was .98 (95% C.I= .96-.99) indicating excellent agreement; for AE Kappa was .96 (95% CI= .87-1.00 in the 'almost perfect' range; for PM Kappa was .65 (95%= CI- .45-.86) between the moderate and substantial agreement, ranges according to Landis & Koch[5].

eMethods 4. Data Completeness and Timing of Assessments

Data were available on over 90% of the sample at each follow up point (328, 91% at 12 weeks and 332, 92% at 24 weeks). The 12-week assessments were conducted at a mean of 13.5 weeks (range: 8.6 to 19.6) and the 24 week assessments with a mean of 25.2 weeks (range: 12.9 to 38.3). Data on unblinding by site is provided in eTable 1.

eTable 1. Unblinding by Site

(Data are n (%). GPTS (Green Paranoid Thoughts Scale[6]) is the primary outcome)

	Participants where unblinding occurred	12W some data collected unblinded	12W GPTS collected unblinded	24W some data collected unblinded	24W GTPS collected unblinded
London	15	9	5	9	4
Sussex	18	8	4	3	2
Oxford	15	5	3	7	5
Total (%)	48 (13.3%)	22 (6.7%)	12 (3.6%)	19 (5.7%)	11 (3.3%)

Note: 12W= 12 Week Follow-up; 24W= 24 Week Follow-up; GPTS= Green Paranoid Thought Scale

eTable 2. Number (Percentage) Above Threshold for a Potential Persecutory Delusion

(criteria from Freeman et al 2019[13]) by timepoint and randomised arm.

% refers to number of observed outcomes at each timepoint

Time	Group	GPTS Part B Persecution		R-GPTS Part B	
		<35	≥35	<18	≥18
Baseline	TAU	10 (5.6)	170 (94.4)	45 (25.0)	135 (75.0)
	SlowMo	11 (6.1)	169 (93.9)	54 (30.0)	126 (70.0)
12 Weeks	TAU	45 (27.6)	118 (72.4)	68 (41.7)	95 (58.3)
	SlowMo	57 (34.3)	109 (65.7)	87 (52.4)	79 (47.6)
24 Weeks	TAU	54 (31.6)	117 (68.4)	85 (50.3)	84 (49.7)
	SlowMo	62 (38.5)	99 (61.5)	91 (56.5)	70 (43.5)

©2021 Garety P et al. *JAMA Psychiatry*.

eFigure 2. Forest Plot Binary Outcomes

Supplementary eFigure 1 shows a forest plot for the binary outcomes: JTC (85:15 and 60:40; extreme responding) and Belief Flexibility (Possibility of being mistaken and presence if Alternative Explanations; both scored yes/no).

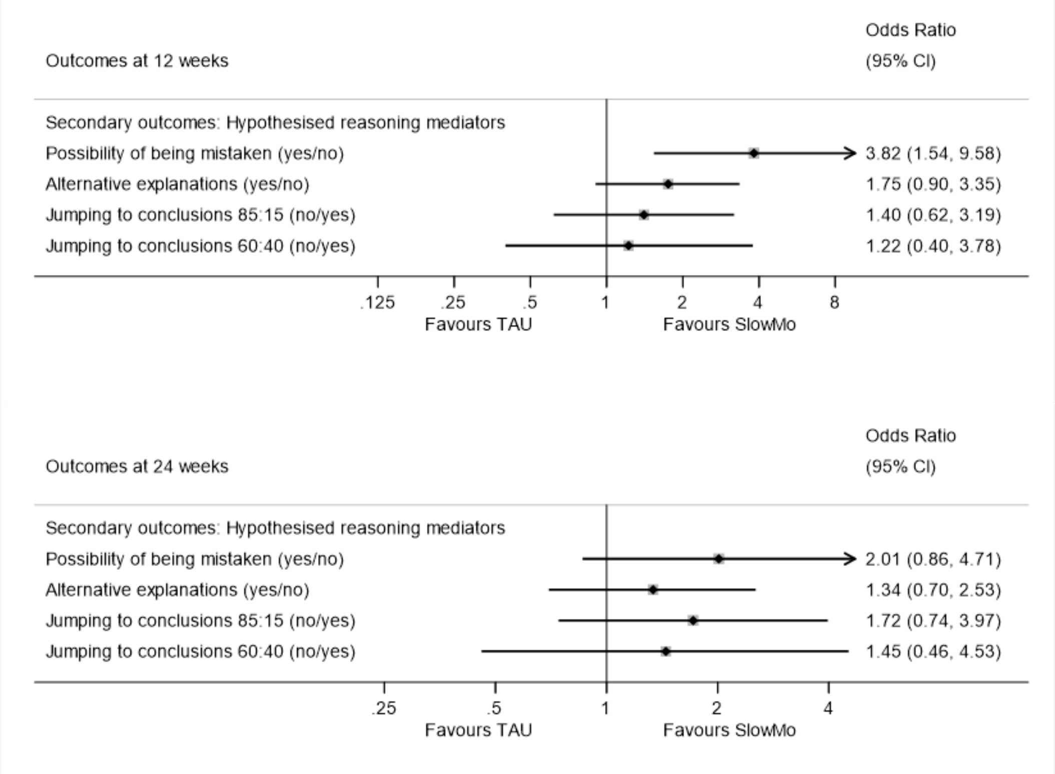

eTable 3. Baseline Characteristics by Site

Baseline characteristics		London (n=130)	Oxford (n=99)	Sussex (n=132)	Overall (N=361)
**Age†**		44.5 (11.0)	42.8 (10.9)	40.7 (12.4)	42.6 (11.6)
**Sex**	*Male*	82 (63.1)	71 (71.7)	99 (75.0)	252 (69.8)
	Female	48 (36.9)	28 (28.3)	33 (25.0)	109 (30.2)
**Marital Status**	*Single*	106 (81.5)	74 (74.7)	102 (77.3)	282 (78.1)
	Cohabiting	1 (0.8)	3 (3.0)	8 (6.1)	12 (3.3)
	Married or Civil Partnership	12 (9.2)	18 (18.2)	16 (12.1)	46 (12.7)
	Divorced	10 (7.7)	3 (3.0)	4 (3.0)	17 (4.7)
	Widowed	1 (0.8)	1 (1.0)	2 (1.5)	4 (1.1)
**Self-defined Ethnicity**	*White*	49 (37.7)	85 (85.9)	115 (87.1)	249 (69.0)
	Black Caribbean	15 (11.5)	2 (2.0)	1 (0.8)	18 (5.0)
	Black African	17 (13.1)	2 (2.0)	3 (2.3)	22 (6.1)
	Black Other	25 (19.2)	1 (1.0)	2 (1.5)	28 (7.8)
	Indian	2 (1.5)	1 (1.0)	0 (0.0)	3 (0.8)
	Pakistani	3 (2.3)	2 (2.0)	3 (2.3)	8 (2.2)
	Chinese	0 (0.0)	0 (0.0)	1 (0.8)	1 (0.3)
	Other	19 (14.6)	6 (6.1)	6 (4.5)	31 (8.6)

©2021 Garety P et al. *JAMA Psychiatry*.

Baseline characteristics		London (n=130)	Oxford (n=99)	Sussex (n=132)	Overall (N=361)
	Missing	0 (0.0)	0 (0.0)	1 (0.8)	1 (0.3)
**Highest level of schooling**	*Primary school*	7 (5.4)	0 (0.0)	0 (0.0)	7 (1.9)
	Secondary, no exams or qualifications	20 (15.4)	21 (21.2)	23 (17.4)	64 (17.7)
	Secondary O/CSE equivalent	33 (25.4)	32 (32.3)	36 (27.3)	101 (28.0)
	Secondary A-level equivalent	14 (10.8)	18 (18.2)	7 (5.3)	39 (10.8)
	Vocational education/college	35 (26.9)	13 (13.1)	39 (29.5)	87 (24.1)
	University degree/professional qualification	19 (14.6)	15 (15.2)	27 (20.5)	61 (16.9)
	Missing	2 (1.5)	0 (0.0)	0 (0.0)	2 (0.6)
**Current working status**	*Unemployed*	113 (86.9)	73 (73.7)	105 (79.5)	291 (80.6)
	Employed full-time	3 (2.3)	7 (7.1)	6 (4.5)	16 (4.4)
	Employed part-time	8 (6.2)	9 (9.1)	12 (9.1)	29 (8.0)
	Self-employed	1 (0.8)	4 (4.0)	1 (0.8)	6 (1.7)
	Retired	3 (2.3)	4 (4.0)	5 (3.8)	12 (3.3)
	Student	2 (1.5)	1 (1.0)	1 (0.8)	4 (1.1)
	Housewife/husband	0 (0.0)	1 (1.0)	2 (1.5)	3 (0.8)
**Normal living situation**	*Living alone*	94 (72.3)	50 (50.5)	67 (50.8)	211 (58.4)
	Living with partner	10 (7.7)	17 (17.2)	20 (15.2)	47 (13.0)
	Living with parents	12 (9.2)	20 (20.2)	23 (17.4)	55 (15.2)
	living with other relatives	6 (4.6)	0 (0.0)	2 (1.5)	8 (2.2)
	Living with others	8 (6.2)	12 (12.1)	20 (15.2)	40 (11.1)
**GPTS Part B (stratification factor)**	Below 62	78 (60.0)	61 (61.6)	80 (60.6)	219 (60.7)
	62 and over	52 (40.0)	38 (38.4)	52 (39.4)	142 (39.3)
**Diagnosis**					
	Schizophrenia	79 (60.8)	65 (65.7)	81 (61.4)	225 (62.3)
	Schizoaffective	28 (21.5)	20 (20.2)	16 (12.1)	64 (17.7)
	Delusional Disorder	1 (1)	3 (3.0)	2 (1.5)	6 (1.7)
	Psychosis (other)	22 (16.9)	11 (11.1)	33 (25.0)	66 (18.2)
**Time in contact with services**	<1 year	2 (1.5)	1 (1.0)	10 (7.6)	13 (3.6)
	1-5 years	16 (12.3)	15 (15.2)	24 (18.2)	55 (15.2)
	6-10	23 (17.7)	12 (12.1)	49 (37.1)	84 (23.3)
	11-20	46 (35.4)	49 (49.5)	45 (34.1)	140 (38.8)
	20+	43 (33.1)	22 (22.2)	4 (3.0)	69 (19.1)
**Chlorpromazine-equivalent dose of antipsychotic drug (mg/day) †**		417.1 (329.1)	643.6 (513.3)	436.6 (365.3)	486.4 (410.5)

Data are n(%) or †mean (SD)

©2021 Garety P et al. *JAMA Psychiatry*.

eMethods 5. Moderation Analysis: Further Detail

The following measures of baseline clinical and cognitive characteristics were potential moderators of treatment effects: Brief Negative Symptom Scale;[7] Letter Number Sequencing Test (assessing working memory)[8]; Scales for Assessment of Positive Symptoms;[9] Beliefs about Problems Questionnaire, (assessing illness and treatment perceptions);[10] Trail Making Test[11] (assessing visual attention, psychomotor speed, and shifting cognitive set); and Perception of Carer Criticism, single self-reported item.[12] We also examined demographic variables as moderators: age, sex and self-defined ethnicity. Following a request from a reviewer, we included site as an additional posthoc moderator.

We investigated the specific hypothesis that poorer working memory (Letter number sequencing test), and more severe negative symptoms (Brief Negative Symptoms Scale, BNSS), will negatively moderate treatment effects on GPTS, R-GPTS and PSYRATS. The moderation analyses also investigated whether the effect of the SlowMo intervention on GPTS, R-GPTS and PSYRATS was moderated by: baseline measure of the outcomes; reasoning: belief flexibility (Possibility of being mistaken and Alternative Explanations) and jumping to conclusions (beads task 85:15, more than two beads drawn yes/no); and beliefs about problems and treatment (BAPQ), set-shifting (Trail making task (B-A)), presence of a carer (yes/no), perceived criticism of carer (if present). We also tested for moderation by age (<35, 35-50, >50), gender (male/female),ethnicity (white/black/Asian and other) and site (London/Oxford/Sussex).

For a continuous moderator, the difference in treatment effect between unit levels of the moderator can be interpreted as the difference in the estimated treatment effect between a participant with a moderator value at baseline of $a+1$ and a participant with a moderator value at baseline of a. For a binary moderator (e.g. presence of a carer), the difference in treatment effect can be interpreted as the difference in the estimated treatment effect between participant with a carer and those participants without a carer. Baseline clinical and cognitive characteristics of potential moderators are provided in Supplementary eTable 4.

The moderation analysis (Supplementary eTable 5) found no differential effects on paranoia as measured by the GPTS or R-GPTS.[13] There were two moderation effects (on PSYRATS), at $p<0.05$. However, given the number of tests, this finding may have occurred by chance. These results indicate that treatment effects were not moderated by clinical or demographic variables, indicating benefits regardless of cognitive capacity, including working memory, symptoms including negative symptoms, or family relationships. At the request of a reviewer, we conducted a posthoc test of moderation by clinical site, and found no significant differential effects.

eTable 4. Baseline Clinical and Cognitive Characteristics as Potential Moderators of the Intention-to-Treat Population

Data are mean (SD); number of observations or number of observations (%)

	SlowMo (n=181)	TAU (n=180)	Overall (n=361)
BNSS total	7.0 (8.4); 179	5.8 (8.1); 179	6.4 (8.2); 358
BAPQ total	47.4 (6.4); 179	48.0 (5.5); 177	47.7 (6.0); 356
LNS raw score	7.6 (2.9); 176	8.2 (3.0); 171	7.9 (3.0); 347
Trail making task (B-A)	69.7 (47.4); 157	63.3 (44.8); 160	66.5 (46.1); 317
Trail making part A	40.9 (16.9); 165	41.7 (20.2); 163	41.3 (18.6); 328
Trail making part B	110.6 (54.5); 165	105.0 (52.6); 163	107.8 (53.5); 328
Carer N (%)			
No	75 (41.9)	72 (40.2)	147 (41.1)
Yes	104 (58.1)	107 (59.8)	211 (58.9)
How critical is your carer N (%)			
0. Not at all	37 (35.6)	30 (28.8)	67 (32.2)
1.	11 (10.6)	12 (11.5)	23 (11.1)
2.	18 (17.3)	17 (16.3)	35 (16.8)
3.	19 (18.3)	20 (19.2)	39 (18.8)
4.	10 (9.6)	18 (17.3)	28 (13.5)
5. Extremely	9 (8.7)	7 (6.7)	16 (7.7)

Abbreviations: BNSS= Brief Negative Symptoms Scale[7]; BAPQ = Beliefs about Problems questionnaire[10]; LNS= Letter Number Sequencing Test[8]

©2021 Garety P et al. *JAMA Psychiatry*.

eTable 5. Moderation Analysis Results

Moderator	Time	Outcome		
		GPTS	R-GPTS	PSYRATS
**Baseline outcome**				
	Week 12	-0.08 (-0.28, 0.13); 0.469	-0.06 (-0.27, 0.15); 0.568	-0.31 (-0.62, -0.01); 0.045
	Week 24	-0.12 (-0.32, 0.09); 0.263	-0.12 (-0.33, 0.09); 0.271	-0.24 (-0.54, 0.07); 0.129
**BNSS**				
	Week 12	0.27 (-0.41, 0.95); 0.439	0.02 (-0.37, 0.42); 0.910	-0.01 (-0.13, 0.11); 0.922
	Week 24	-0.10 (-0.78, 0.58); 0.777	-0.07 (-0.47, 0.32); 0.708	0.06 (-0.06, 0.18); 0.317
**BAPQ**				
	Week 12	0.06 (-0.89, 1.02); 0.896	0.08 (-0.46, 0.62); 0.775	0.07 (-0.09, 0.24); 0.383
	Week 24	0.10 (-0.85, 1.06); 0.833	0.02 (-0.52, 0.56); 0.934	0.06 (-0.10, 0.23); 0.452
**Letter-Number raw score**				
	Week 12	-0.44 (-2.31, 1.42); 0.641	-0.21 (-1.26, 0.85); 0.697	-0.21 (-0.54, 0.12); 0.210
	Week 24	-0.21 (-2.06, 1.63); 0.822	-0.09 (-1.14, 0.96); 0.867	-0.30 (-0.62, 0.03); 0.075
**Trail making task (B-A)**				
	Week 12	0.05 (-0.08, 0.18); 0.436	0.03 (-0.04, 0.10); 0.448	0.00 (-0.02, 0.02); 0.889
	Week 24	0.04 (-0.09, 0.16); 0.574	0.01 (-0.06, 0.09); 0.701	0.02 (-0.00, 0.04); 0.069
**Presence of a carer (yes vs no)**				
	Week 12	7.71 (-3.56, 18.98); 0.180	3.70 (-2.67, 10.07); 0.255	1.12 (-0.85, 3.08); 0.265
	Week 24	1.23 (-10.01, 12.46); 0.831	0.84 (-5.54, 7.22); 0.796	0.48 (-1.47, 2.44); 0.628
**Criticism of carer (only if carer present, n=208)**				
	Week 12	-0.28 (-4.73, 4.17); 0.903	-0.14 (-2.65, 2.38); 0.915	-0.62 (-1.41, 0.17); 0.122
	Week 24	2.48 (-1.93, 6.88); 0.270	1.32 (-1.16, 3.81); 0.297	0.14 (-0.64, 0.92); 0.716
**Alternative explanations (yes vs no)**				
	Week 12	2.62 (-8.50, 13.75); 0.644	1.99 (-4.29, 8.27); 0.534	1.15 (-0.79, 3.09); 0.245
	Week 24	4.39 (-6.69, 15.46); 0.438	2.29 (-3.98, 8.57); 0.474	-0.12 (-2.05, 1.81); 0.905
**Possibility of being mistaken (y/n)**				
	Week 12	3.88 (-7.49, 15.24); 0.504	3.07 (-3.36, 9.51); 0.349	0.79 (-1.17, 2.76); 0.429
	Week 24	2.71 (-8.60, 14.02); 0.639	1.67 (-4.76, 8.10); 0.610	0.16 (-1.79, 2.12); 0.871
**Jumping to conclusions (yes vs no)**				
	Week 12	-0.72 (-11.97, 10.52); 0.90	-0.75 (-7.10, 5.60); 0.817	-0.33 (-2.29, 1.63); 0.744
	Week 24	7.63 (-3.59, 18.85); 0.183	5.76 (-0.60, 12.11); 0.076	1.12 (-0.84, 3.07); 0.264
**Age (<35 vs 35-50)**				

©2021 Garety P et al. *JAMA Psychiatry*.

Moderator	Time	Outcome		
		GPTS	R-GPTS	PSYRATS
	Week 12	13.30 (-0.15, 26.76); 0.053	7.43 (-0.15, 15.00); 0.055	1.37 (-0.98, 3.71); 0.253
	Week 24	3.58 (-9.79, 16.95); 0.600	1.43 (-6.12, 8.98); 0.710	0.16 (-2.16, 2.49); 0.890
**Age (< 35 vs >50)**				
	Week 12	-4.51 (-18.89, 9.88); 0.539	-3.53 (-11.67, 4.60); 0.395	-0.72 (-3.24, 1.81); 0.578
	Week 24	1.49 (-12.81, 15.79); 0.838	-0.08 (-8.19, 8.02); 0.984	0.15 (-2.35, 2.64); 0.908
**Gender (male vs female)**				
	Week 12	-6.68 (-18.83, 5.47); 0.281	-2.79 (-9.64, 4.06); 0.425	-1.84 (-3.97, 0.29); 0.09
	Week 24	-4.73 (-16.94, 7.48); 0.447	-2.65 (-9.59, 4.28); 0.453	-0.69 (-2.83, 1.45); 0.526
**Ethnicity (white vs black)**				
	Week 12	-8.02 (-22.66, 6.63); 0.283	-4.52 (-12.78, 3.74); 0.284	-2.64 (-5.20, -0.09); 0.043
	Week 24	-1.16 (-15.75, 13.44); 0.876	2.53 (-5.71, 10.76); 0.547	-1.31 (-3.85, 1.23); 0.310
**Ethnicity (white vs Asian/other)**				
	Week 12	5.91 (-11.55, 23.37); 0.507	3.93 (-5.89, 13.74); 0.432	-0.00 (-3.04, 3.03); 0.998
	Week 24	4.67 (-12.78, 22.12); 0.600	3.80 (-6.09, 13.69); 0.452	-1.83 (-4.88, 1.21); 0.238
**Site (London vs Oxford)**				
	Week 12	-2.57 (-16.63, 11.48); 0.719	-1.32 (-9.22, 6.57); 0.743	-0.22 (-2.65, 2.22); 0.862
	Week 24	-2.80 (-16.86, 11.26); 0.696	-3.39 (-11.34, 4.56); 0.404	0.31 (-2.13, 2.75); 0.803
**Site (London vs Sussex)**				
	Week 12	-2.82 (-16.00, 10.36); 0.675	-1.53 (-8.99, 5.93); 0.688	0.73 (-1.57, 3.02); 0.534
	Week 24	-1.76 (-14.84, 11.33); 0.793	-1.95 (-9.36, 5.46); 0.607	1.81 (-0.46, 4.09); 0.118

Note: Data show difference in treatment effect between unit levels of the moderator (95% CI); p-value. Abbreviations: GPTS= Green Paranoid Thoughts Scale[6]; R-GPTS= Revised Green Paranoid Thoughts Scale[13]; PSYRATS= The Psychotic Symptom Rating Scales[14]; dis= distress; conv= conviction; SAPS= Scale for the Assessment of Positive Symptoms[9]; BNSS= Brief Negative Symptoms Scale[7]; BAPQ = Beliefs about Problems questionnaire[10]

©2021 Garety P et al. *JAMA Psychiatry*.

eMethods 6. Mediation Analysis: Further Detail

Mediation analyses examined potential mechanisms underlying the effect of SlowMo compared to TAU on clinical paranoia outcomes, GPTS, R-GPTS and PSYRATS. We examined the specific hypotheses:
Reductions in fast thinking (belief flexibility and Jumping to conclusions) will mediate improvement in paranoia severity; and worry will not mediate reductions in paranoia severity, Jumping to Conclusions, belief flexibility (Possibility of being mistaken and Alternative Explanations) and worry at 12 weeks were individually considered as mediators of the effect on the outcomes at 12 and 24 weeks separately. The analysis used causal mediation analysis based on parametric regression models.[15] For each mediator separately, this involved estimating a linear model for each mediator with random assignment, baseline outcome, baseline mediator, site and paranoia cut-off at baseline as covariates, and separately estimating a linear model for each outcome with the mediator, group assignment, baseline outcome, baseline mediator, site and paranoia cut-off as covariates. The effect of group assignment on the mediator is multiplied by the effect of mediator on outcome to estimate the indirect effect, and the effect of SlowMo on outcome in the model including mediator is an estimate of the direct effect. The indirect and direct effects sum to the total effect, and bootstrapping with 500 replications was used to obtain valid standard errors for the causal effects. 95% confidence intervals are based on the percentile of the bootstrap distribution. The proportion mediated is the indirect effect divided by the total effect.
The results of the mediation analysis on the GPTS, PSYRATS, and R-GPTS at 12 and 24 weeks are shown in Supplementary eTables 6-8). Only possibility of being mistaken (PM[16]) and worry[17] mediated the effects of the treatment on all paranoia outcomes at 12 and 24 weeks. Approximately 40% of the total effect was mediated through each mediator at 12 weeks and 56% at 24 weeks.

eTable 6. Mediation Effects of SlowMo on GPTS: Mediator Variables at 12 Weeks and GPTS at 12 and 24 Weeks

Effects show: causal mediation effect (bootstrap SE); 95% confidence interval

Mediator (12wks)	Time	Total effect	Direct effect	Indirect effect	Proportion mediated
Alternative explanations					
	12	-7.44 (2.98); -13.32, -1.14	-7.01 (2.94); -12.81, -0.67	-0.43 (0.44); -1.46, 0.15	5.8
	24	-4.86 (2.90); -10.74, 0.92	-4.55 (2.84); -10.12, 1.03	-0.31 (0.38); -1.33, 0.26	6.4
JTC - 85:15 task					
	12	-7.24 (3.09); -13.03, -0.64	-6.89 (3.04); -12.80, -0.83	-0.34 (0.49); -1.27, 0.58	4.7
	24	-4.02 (2.94); -9.69, 1.87	-3.76 (2.92); -9.31 (2.06)	-0.26 (0.41); -1.14, 0.63	6.5
JTC - 60:40 task					
	12	-7.63 (3.05); -13.61, -0.98	-7.55 (3.04); -13.70, -1.00	-0.09 (0.44); -0.99, 0.80	1.1
	24	-4.60 (2.91); -10.14, 1.29	-4.57 (2.90); -10.12, 1.42	-0.03 (0.22); -0.48, 0.44	0.7
Possibility of being mistaken (yes/no)					
	12	-8.35 (2.99); -14.13, -2.07	-6.00 (2.93); -11.86, 0.05	-2.35 (1.08); -4.71, -0.59	28.1
	24	-5.26 (2.92); -11.14, 0.53	-3.55 (2.78); -8.67, 1.96	-1.71 (0.92); -3.93, -0.39	32.5
Possibility of being mistaken (1-100)					
	12	-7.58 (2.98); -13.44, -1.01	-4.86 (2.83); -10.21, 0.97	-2.72 (1.07); -5.04, -0.91	35.9
	24	-4.89 (2.89); -10.30, 1.12	-2.13 (2.69); -7.51, 3.39	-2.76 (1.02); -4.75, -0.75	56.4
Worry					
	12	-7.78 (3.00); -13.63, -1.17	-4.74 (2.96); -10.44, 1.74	-3.04 (1.10); -5.52, -1.09	39.1
	24	-4.46 (2.90); -10.42, 1.12	-1.95 (2.91); -7.48, 4.02	-2.51 (1.11); -5.13, -0.97	56.3

Abbreviation: JTC= Jumping to Conclusion[18]

©2021 Garety P et al. *JAMA Psychiatry*.

eTable 7. Mediation Effects of SlowMo: Mediator Variables at 12 Weeks and PSYRATS at 12 and 24 Weeks

Effects show: causal mediation effect (bootstrap SE); 95% confidence interval.

Mediator (12wks)	Time	Total effect	Direct effect	Indirect effect	Proportion mediated
Alternative explanations	12	-1.52 (0.49); -2.49, -0.62	-1.47 (0.48); -2.40, -0.56	-0.04 (0.06); -0.19, 0.05	2.6
	24	-4.12 (1.69); -7.39, -0.44	-3.82 (1.71); -7.12, 0.04	-0.30 (0.28); -0.99, 0.09	7.3
JTC - 85:15 task	12	-1.45 (0.50); -2.42, -0.50	-1.40 (0.48); -2.32, -0.48	-0.05 (0.08); -0.24, 0.08	3.4
	24	-3.77 (1.72); -6.81, 0.11	-3.61 (1.73); -6.66, 0.21	-0.17 (0.24); -0.64, 0.38	4.5
JTC - 60:40 task	12	-1.50 (0.50); -2.49, -0.51	-1.49 (0.49); -2.47, -0.49	-0.01 (0.05); -0.12, 0.09	0.7
	24	-4.14 (1.69); -7.32, -0.61	-4.12 (1.71); -7.42, -0.56	-0.03 (0.16); -0.35, 0.39	0.7
Possibility of being mistaken (yes/no)	12	-1.64 (0.49); -2.56, -0.65	-1.34 (0.47); -2.21, -0.43	-0.30 (0.14); -0.60, -0.07	18.3
	24	-4.49 (1.67); -7.64, -0.98	-3.57 (1.65); -6.77, -0.35	-0.92 (0.52); -2.24, -0.16	20.5
Possibility of being mistaken (1-100)	12	-1.52 (0.48); -2.43, -0.59	-1.08 (0.45); -1.98, -0.22	-0.44 (0.17); -0.81, -0.12	28.9
	24	-4.04 (1.68); -7.21, -0.30	-2.69 (1.67); -5.96, 0.71	-1.35 (0.58); -2.64, -0.34	33.4
Worry	12	-1.55 (0.50); -2.49, -0.63	-1.10 (0.48); -1.97, -0.18	-0.45 (0.16); -0.83, -0.16	29.0
	24	-3.77 (1.65); -6.97, -0.40	-2.57 (1.66); -5.80, 0.85	-1.20 (0.52); -2.35, -0.32	31.8

©2021 Garety P et al. *JAMA Psychiatry*.

eTable 8. Mediation Effects of SlowMo: Mediator Variables at 12 Weeks and R-GPTS at 12 and 24 Weeks

Effects show: causal mediation effect (bootstrap SE); 95% confidence interval.

Mediator (12wks)	Time	Total effect	Direct effect	Indirect effect	Proportion mediated
Alternative explanations	12	-4.63 (1.68); -8.10, -1.48	-4.38 (1.67); -7.65, -1.19	-0.25 (0.25); -0.86, 0.09	5.4%
	24	-3.42 (1.71); -6.61, -0.10	-3.18 (1.70); -6.40, 0.13	-0.25 (0.25); -0.83, 0.11	7.3
JTC - 85:15 task	12	-4.61 (1.73); -7.91, -1.31	-4.44 (1.72); -7.72, -1.17	-0.17 (0.28); -0.83, 0.32	3.7%
	24	-3.01 (1.75); -6.07, 0.51	-2.85 (1.75); -5.97, 0.79	-0.15 (0.25); -0.63, 0.39	5.0
JTC - 60:40 task	12	-4.72 (1.72); -7.98, -1.44	-4.70 (1.68); -7.88, -1.50	-0.03 (0.25); -0.57, 0.43	0.6%
	24	-3.32 (1.72); -6.74, 0.17	-3.31 (1.73); -6.71, 0.08	-0.01 (0.15); -0.31, 0.38	0.3
Possibility of being mistaken (yes/no)	12	-5.14 (1.70); -8.48, -1.88	-3.80 (1.65); -7.17, -0.58	-1.34 (0.60); -2.73, -0.36	26.1%
	24	-3.65 (1.71); -6.80, -0.26	-2.63 (1.68); -5.84, 0.70	-1.02 (0.54); -2.37, -0.26	27.9
Possibility of being mistaken (1-100)	12	-4.76 (1.67); -8.03, -1.62	-3.20 (1.57); -6.28, -0.37	-1.55 (0.62); -2.86, -0.39	32.6%
	24	-3.28 (1.70); -6.50, 0.16	-1.70 (1.61); -4.80, 1.46	-1.58 (0.61); -2.78, -0.43	48.2
Worry	12	-4.87 (1.63); -8.10, -1.84	-3.17 (1.60); -6.18, 0.01	-1.71 (0.62); -2.95, -0.58	35.1%
	24	-3.14 (1.67); -6.39, 0.10	-1.75 (1.66); -4.74, 1.46	-1.39 (0.57); -2.64, -0.52	44.3

eMethods 7. Adverse Events

Adverse events were actively monitored for the duration of the study, up to 24 week follow-up. These included hospital admissions (due to physical or mental health deterioration), crisis team involvement, self-harming behaviour and suicide attempts, and violent incidents necessitating police involvement. A standard reporting method categorised events by severity (five grades, A–E). Any relatedness to trial participation was also recorded. All adverse events and associated ratings were reviewed by the chairperson of the Data Monitoring and Ethics Committee (DMEC) and subsequently by the DMEC.

Fifty-four adverse events were reported during the trial, of which 51 were serious, occurring in 19 people in the SlowMo group and 21 in the control group (eTable 9). No deaths were recorded. One serious adverse event in the control arm was rated as 'definitely related' to trial involvement: it involved a complaint when the research team shared information with the clinical team under a duty of care (confirmed by independent ethical review). (The participant subsequently requested to withdraw data and is therefore a 'post-randomisation exclusion' in the analysis.)

eTable 9. Adverse Events and Trial-Related Adverse Events

Data are N (%)

Adverse events	SlowMo	TAU
Serious events (people)		
Yes	25 (19)	26* (21)
No	3 (3)	0 (0)
Adverse event type events (people)		
Physical	8 (8)	2 (2)
Self Harm	1 (1)	0 (0)
Serious violent incidents (victim)	0 (0)	1 (1)
Serious violent incidents (accused)	1 (1)	2 (2)
Referrals to crisis care	5 (5)	2 (2)
Admission to psychiatric hospital during follow-up	8 (8)	14 (10)
Deaths	0 (0)	0 (0)
Other	5 (5)	5 (4)
Intensity of events (%)		
Mild	2 (7.1)	0 (0.0)
Moderate	11 (39.3)	10 (38.5)
Severe	15 (53.6)	16 (61.5)
Relationship to trial participation (Serious events)		
Definitely related	0 (0.0)	1 (3.8)
Probably related	0 (0.0)	0 (0.0)
Possibly related	1 (4.0)	0 (0.0)
Unlikely related	1 (4.0)	0 (0.0)
Not related	23 (92.0)	25 (96.2)

**included one event from an individual who subsequently requested to be withdrawn from data analysis (i.e. post-randomisation exclusion)*

eMethods 8. Compliance Analysis

In order to examine the following research question we conducted a compliance-adjusted analysis: Does outcome differ by adherence to the intervention and is adherence predicted by the participants' beliefs about their illness and about the intervention?

To account for departures from random allocation in the SlowMo group who received therapy, we performed two compliance-adjusted analyses for a binary compliance measure (attending at least 1 session of SlowMo therapy) and a continuous measure of number of sessions received. Both analyses used a two stage instrumental variables approach that involved in the first stage regressing the treatment receipt measure on randomisation, baseline GPTS[6], site and paranoia and saving the predicted value of the treatment receipt measure. In the second stage, this predicted value is included in the analysis models in place of the randomisation variable. Both models were estimated in a single bootstrap procedure to produce valid standard errors for the effect of treatment received, with resampling at the level of participant.

The first measure of compliance indicates anyone who receives at least one session of therapy. The treatment effect is interpreted as the Complier Average Causal Effect, where complier is defined as those participants randomised to SlowMo who received at least 1 session of therapy and those participants randomised to TAU who would have received at least 1 session of therapy had they been randomised to SlowMo (a counterfactual, based on predictions from a model). The treatment effect is the adjusted mean difference between randomised arms within this subgroup of compliers.
The second measure of compliance is the number of sessions of therapy attended. This is observed for all participants in the SlowMo arm (ranging from 0 to 8) and is fixed by design at 0 in the TAU group. The treatment effect is the effect of one additional session of therapy on the outcome, assuming a linear effect e.g. going from s sessions to $s+1$ sessions for any s between 0 and 8. Details of the statistical approach for mediation analysis and departures from random allocation are outlined in Dunn et al.[19]

The results of the compliance adjusted analysis on the GPTS, R-GPTS and PSYRATS are shown in Supplementary eTable 10. Since there is no access to SlowMo therapy from the TAU group, the Complier Average Causal Effect is an adjustment to the ITT effect for each outcome divided by the predicted proportion of those in

©2021 Garety P et al. *JAMA Psychiatry*.

the SlowMo arm who were observed to attend at least 1 session of therapy. The results show significant treatment effects of SlowMo therapy compared to TAU in the compliers at all time points. The dose-response effect shows the treatment effect increases as the number of SlowMo sessions increases.

Is adherence predicted by the participants' beliefs about their illness and about the intervention?

We also tested the hypothesis that compliance would be predicted by participants' belief about their illness and the intervention (as measured by the Beliefs about Problems questionnaire): we found that BAPQ is not a significant predictor of either attending any therapy (p=0.370) or the number of sessions (p=0.589).

eTable 10. Analysis of Treatment Received

Outcome	Time	Compliance measure	
		Any sessions (≥1)	Number of sessions
GPTS	12	-8.73 (2.52); 0.001<br>-13.68, -3.79	-1.19 (0.32); <0.001<br>-1.83, -0.56
	24	-5.64 (2.47); 0.022<br>-10.47, -0.81	-0.77 (0.34); 0.024<br>-1.44, -0.10
R-GPTS	12	-5.57 (1.40); <0.001<br>-8.32, -2.83	-0.76 (0.19); <0.001<br>-1.14, -0.38
	24	-3.79 (1.41); 0.007<br>-6.56, -1.02	-0.52 (0.20); 0.010<br>-0.91, -0.12
PSYRATS	12	-1.64 (0.39); <0.001<br>-2.41, -0.87	-0.22 (0.05); <0.001<br>-0.33, -0.12
	24	-1.79 (0.42); <0.001<br>-2.61, -0.96	-0.24 (0.06); <0.001<br>-0.37, -0.12

Note: Data shows treatment effect (bootstrap SE); p-value and 95% confidence interval. Low score indicates better outcomes; negative effects indicate benefit of SlowMo compared to TAU. Abbreviations: GPTS= Green Paranoid Thoughts Scale[6]; R-GPTS= Revised Green Paranoid Thoughts Scale[13]; PSYRATS= The Psychotic Symptom Rating Scale[14]

eMethods 9. Concomitant Therapy and Medication and Service Use Data

Concomitant treatments (psychosocial, psychological therapy and medications; reported in Supplementary Table 11) and services (days in crisis care and hospital admission; reported in Supplementary Table 12) provided as usual care were monitored from case notes using a modified version of the Client Service Receipt Inventory CSRI.

eTable 11. Concomitant Therapy and Medication

Data show number of events (people). Sessions shows mean number of sessions (SD)

	SlowMo	TAU
1:1 CBT (paranoia focus)	7 (7)	12 (11)
Sessions	7.6 (10.0)	7.0 (5.4)
1:1 psychology (non-paranoia focus)	8 (8)	26 (25)
Sessions	4.7 (3.5)	9.1 (6.2)
Psychology groups (paranoia)	2 (2)	2 (2)
Sessions	3.5 (2.1)	1.0 (0.0)
Psychology group (non-paranoia)	6 (6)	7 (7)
Sessions	7.0 (8.6)	7.3 (7.9)
Family intervention	2 (2)	4 (4)
Sessions	2.5 (2.1)	6.5 (3.4)
Health and well-being groups	3 (3)	8 (6)
Sessions	7.7 (10.7)	4.5 (2.1)
1:1 therapy (non-psychology)	1 (1)	2 (2)
Sessions	3.0 (.)	14.0 (4.2)
Art Therapy	1 (1)	2 (2)
Sessions	11.0 (.)	21.5 (0.7)
Other	1 (1)	7 (7)

©2021 Garety P et al. *JAMA Psychiatry*.

	SlowMo	TAU
Sessions	6.0 (.)	8.4 (10.0)
Typical Antipsychotic	29 (20)	25 (22)
Atypical Antipsychotic	225 (142)	198 (141)
Clozapine	27 (23)	39 (31)
Mood stabiliser	26 (23)	26 (23)
Antidepressant	74 (67)	82 (70)
Antianxiety/hypnotic	24 (20)	28 (26)
Other psychiatric	34 (28)	21 (15)
Reported non-psychiatric	9 (4)	18 (10)

©2021 Garety P et al. *JAMA Psychiatry*.

eTable 12. Service Use Over Preceding 6 Months at Baseline and Follow-up

Data show N (%) or Total number of hospital admissions (in days).

	Baseline			Follow-up		
	**SlowMo N (%)**	**TAU N (%)**	**Overall N (%)**	**SlowMo N (%)**	**TAU N (%)**	**Overall N (%)**
Home treatment (days)						
0	150 (82.9)	149 (83.7)	299 (83.3)	151 (89.3)	162 (92.0)	313 (90.7)
1	6 (3.3)	3 (1.7)	9 (2.5)	7 (4.1)	1 (0.6)	8 (2.3)
2	13 (7.2)	19 (10.7)	32 (8.9)	11 (6.5)	11 (6.2)	22 (6.4)
3	3 (1.7)	2 (1.1)	5 (1.4)	0 (0.0)	1 (0.6)	1 (0.3)
4	7 (3.9)	4 (2.2)	11 (3.1)	0 (0.0)	1 (0.6)	1 (0.3)
6	1 (0.6)	0 (0.0)	1 (0.3)	0 (0.0)	0 (0.0)	0 (0.0)
7	0 (0.0)	1 (0.6)	1 (0.3)	0 (0.0)	0 (0.0)	0 (0.0)
8	1 (0.6)	0 (0.0)	1 (0.3)	0 (0.0)	0 (0.0)	0 (0.0)
Total days	83	70	153	29	30	59
Hospital Admission (days)						
0 -10	162 (89.5)	155 (87.1)	317 (88.3)	159 (94.1)	166 (94.3)	325 (94.2)
Total days	47	14	61	32	10	42
11-50	11 (6.1)	17 (9.6)	28 (7.8)	5 (3.0)	3 (1.7)	8 (2.3)
Total days	294	424	718	133	45	178
51-100	3 (1.7)	3 (1.7)	6 (1.7)	2 (1.2)	4 (2.3)	6 (1.7)
Total days	225	233	458	132	236	368
>100	5 (2.8)	3 (1.7)	8 (2.2)	3 (1.8)	3 (1.7)	6 (1.7)
Total days	856	414	1270	502	385	887
Total days over all categories	1422	1085	2507	799	676	1475

References

1. Ng MM, Firth J, Minen M, Torous J. User engagement in mental health apps: a review of measurement, reporting, and validity. *Psychiatric Services.* 2019;70(7):538-544.
2. Sanatkar S, Baldwin PA, Huckvale K, et al. Using cluster analysis to explore engagement and e-attainment as emergent behavior in electronic mental health. *Journal of Medical Internet Research.* 2019;21.
3. Sieverink F, Kelders SM, van Gemert-Pijnen JE. Clarifying the Concept of Adherence to eHealth Technology: Systematic Review on When Usage Becomes Adherence. *Journal of medical Internet research.* 2017;19:e402.
4. Garety PA, Ward T, Freeman D, et al. SlowMo, a digital therapy targeting reasoning in paranoia, versus treatment as usual in the treatment of people who fear harm from others: study protocol for a randomised controlled trial. *Trials.* 2017;18(1):510.
5. Landis JR, Koch GG. The measurement of observer agreement for categorical data. *Biometrics.* 1977;33(1):159-174.
6. Green CE, Freeman D, Kuipers E, et al. Measuring ideas of persecution and social reference: the Green et al. Paranoid Thought Scales (GPTS). *Psychol Med.* 2008;38(1):101-111.
7. Kirkpatrick B, Strauss GP, Linh N, et al. The Brief Negative Symptom Scale: Psychometric Properties. *Schizophr Bull.* 2011;37(2):300-305.
8. Wechsler DW. Adult Intelligence Scale – Third Edition (WAIS III). San Antonio: Psychological Corporation; 1997.
9. Andreasen NC. *The Scale of the Assessment of Positive Symptoms (SAPS).* Iowa City, IA: University of Iowa; 1984.
10. Marcus E, Garety P, Weinman J, et al. A pilot validation of a modified Illness Perceptions Questionnaire designed to predict response to cognitive therapy for psychosis. *J Behav Ther Exp Psy.* 2014;45(4):459-466.
11. Lezak MD, Howieson DB, Loring DW, Fischer JS. *Neuropsychological assessment.* Oxford University Press, USA; 2004.
12. Hooley JM, Teasdale JD. Predictors of Relapse in Unipolar Depressives - Expressed Emotion, Marital Distress, and Perceived Criticism. *J Abnorm Psychol.* 1989;98(3):229-235.
13. Freeman D, Loe BS, Kingdon D, et al. The revised Green et al., Paranoid Thoughts Scale (R-GPTS): psychometric properties, severity ranges, and clinical cut-offs. *Psychol Med.* 2019:1-10.
14. Haddock G, McCarron J, Tarrier N, Faragher EB. Scales to measure dimensions of hallucinations and delusions: the psychotic symptom rating scales (PSYRATS). *Psychol Med.* 1999;29(4):879-889.
15. Landau S, Emsley R, Dunn G. Beyond total treatment effects in randomised controlled trials: Baseline measurement of intermediate outcomes needed to reduce confounding in mediation investigations. *Clinical Trials.* 2018;15(3):247-256.
16. Wessely S, Buchanan A, Reed A, et al. Acting on delusions. I: Prevalence. *Brit J Psychiat.* 1993;163(1):69-76.
17. Meyer TJ, Miller ML, Metzger RL, Borkovec TD. Development and Validation of the Penn State Worry Questionnaire. *Behav Res Ther.* 1990;28(6):487-495.
18. Garety PA, Freeman D, Jolley S, et al. Reasoning, emotions, and delusional conviction in psychosis. *J Abnorm Psychol.* 2005;114(3):373-384.
19. Dunn G, Emsley R, Liu HH, et al. Evaluation and validation of social and psychological markers in randomised trials of complex interventions in mental health: a methodological research programme. *Health Technol Asses.* 2015;19(93).

©2021 Garety P et al. *JAMA Psychiatry*.
