# Supplementary material for: Effects of SlowMo, a Blended Digital Therapy Targeting Reasoning, on Paranoia Among People With Psychosis: A Randomized Clinical Trial
Source: JAMA Psychiatry. 2021 Apr 7;78(7):1–13. doi: 10.1001/jamapsychiatry.2021.0326 (PMC8027943; doi:10.1001/jamapsychiatry.2021.0326)
Supplement: Supplement 3. — Data Sharing Statement [file jamapsychiatry-e210326-s003.pdf]

Data Sharing Statement

Garety. Effects of SlowMo, a Blended Digital Therapy Targeting Reasoning, on Paranoia Among People With Psychosis. *JAMA Psychiatry*. Published April 07, 2021. doi:10.1001/jamapsychiatry.2021.0326

Data
Data available: Yes
Data types: Deidentified participant data
How to access data: The datasets generated and/or analysed during the current study will be available in anonymised form from the corresponding author (PAG) on reasonable request, subject to review, following the publication of results.
When available: With publication

Supporting Documents
Document types: Other (please specify)
Additional Information: The following will be provided in the Supplemental material: Supplement 1: SlowMo Trial Protocol & Statistical Analysis Plan (SAP) Supplement 2: Contents 1) SlowMo therapy description 2) SlowMo mobile app adherence criterion 3) Data quality and inter-rater reliability 4) Supplementary eTable 1: Unblinding by site 5) Data completeness, timing of assessments and unmasking 6) Supplementary eTable 2: Number (%) above threshold for a potential persecutory delusion 7) Supplementary eFigure 1: Forest plot binary outcomes 8) Supplementary eTable 3: Baseline characteristics by site 9) Moderation analysis - further detail 10) Supplementary eTable 4: Baseline clinical and cognitive characteristics as potential moderators of the Intention-to-treat population 11) Supplementary eTable 5: Moderation analysis results. 12) Mediation analysis - further detail 13) Supplementary eTable 6: Mediation effects of SlowMo on GPTS. Mediator variables at 12 weeks and GPTS at 12 and 24 weeks. 14) Supplementary eTable 7: Mediation effects of SlowMo. Mediator variables at 12 weeks and PSYRATS at 12 and 24 weeks 15) Supplementary eTable 8: Mediation effects of SlowMo. Mediator variables at 12 weeks and R-GPTS at 12 and 24 weeks 16) Adverse events 17) Supplementary eTable 9: Adverse events and trial-related adverse events 18) Compliance analysis 19) Supplementary eTable 10: Analysis of treatment received 20) Concomitant therapy and medication and Service use data 21) Supplementary eTable 11 - Concomitant therapy and medication 22) Supplementary eTable 12 - Service use over preceding 6 months at baseline and follow-up

How to access documents: These are submitted with the paper as an online supplement
When available: With publication

Additional Information
Who can access the data: The data will be made available only to researchers whose proposed use of the data has been approved
Types of analyses: The data will be made available for scientific research specified in a proposal subject to review and approval by the study team
Mechanisms of data availability: By request to the corresponding author, and subject to review and approval of a proposal.
Any additional restrictions: None.
